# Protective effects of intrathecal injection of AAV9-RabGGTB-GFP^+^ in SOD1^G93A^ mice

**DOI:** 10.3389/fnagi.2023.1092607

**Published:** 2023-03-14

**Authors:** Tianchu Gao, Jia Huo, Cheng Xin, Jing Yang, Qi Liu, Hui Dong, Rui Li, Yaling Liu

**Affiliations:** ^1^Department of Neurology, The Second Hospital of Hebei Medical University, Shijiazhuang, Hebei, China; ^2^The Key Laboratory of Neurology, Hebei Medical University, Ministry of Education, Shijiazhuang, Hebei, China; ^3^Neurological Laboratory of Hebei Province, Shijiazhuang, Hebei, China

**Keywords:** amyotrophic lateral sclerosis, SOD1^G93A^, intrathecal injection of AAV, motoneuron, RabGGTB, SOD1 aggregation

## Abstract

**Introduction:**

Amyotrophic lateral sclerosis (ALS) is a neurodegenerative disease that widely affects motor neurons of the CNS. About 20% of patients with ALS have familial ALS (fALS). One of the classic models of ALS are SOD1^G93A^ mice. Misfolded SOD1 protein can be overexpressed in motor neurons, which results in progressive paralysis of the limbs of mice. There is still no effective treatment for ALS. In recent years, the treatment of ALS by regulating autophagy has become a research hotspot. Autophagy obstacles have been confirmed to be one of the early pathological events of ALS. Rab7 is a member of the Ras superfamily and plays a key role in the late stage of autophagy. In our previous studies, we found that prenoylation of Rab7 was inhibited in the ALS model. Prenylation is a post-translational modification in which farnesyl or geranylgeranyl groups are covalently linked to target proteins. Based on these findings, we proposed the novel idea that the regulation of RabGGTB (the β-subunit of RabGGTase) mediated prenylation modification of Rab7, and that this can be used as a prevention and treatment of ALS associated with abnormal protein accumulation.

**Methods:**

In the present study, RabGGTB was overexpressed in mouse spinal cord motoneurons by using adeno-associated virus as vector. Then immunofluorescence quantitative analysis was used for pathological study. The body weight, footprint analysis, the accelerating rotarod test, and neurological deficits score were used to evaluate animal behavior.

**Results:**

Our results show that the protein level of RabGGTB was significantly increased in the lumbar and thoracic regions of spinal cord motoneurons of injected mice. Furthermore, the onset time and survival time of SOD1^G93A^ mice injected with AAV9-RabGGTB-GFP^+^ were delayed compared with those of mice without overexpression. At the same time, we also observed a decrease in SOD1 misfolded and glial overactivation in the lumbar spinal cord of these SOD1^G93A^ mice.

**Conclusion:**

The findings reported here show that RabGGTB plays a significant role in the pathogenesis of SOD1^G93A^ mice and with great therapeutic potential for reducing abnormal aggregation of SOD1 in ALS.

## 1. Introduction

Motor neuron disease is a group of chronic progressive neurodegenerative diseases that selectively invade anterior horn cells of the spinal cord, motor neurons of the brainstem, cortical pyramidal cells, and the pyramidal tract. Amyotrophic lateral sclerosis (ALS) is the most common motor neuron disease in adults, and is characterized by progressive skeletal muscle weakness, atrophy, and medulla oblongata paralysis. Typically, death caused by respiratory paralysis happens in 3–5 years (Taylor et al., 2016). At present, the pathogenesis of ALS remains unclear and there is no effective treatment (Brown and Al-Chalabi, 2017).

More than 90% of ALS cases are sporadic ALS (sALS), and 5–10% are familial ALS (fALS; Rosen, 1993; Turner et al., 2017). Studies have shown that several genetic mutations, such as those of superoxide dismutase 1 (SOD1), transactivating response element DNA-binding protein-43 (TDP-43), chromosome 9 open reading frame 72 (C9orf72), and fused in sarcoma/translocated in liposarcoma (FUS/TLS), can cause accumulation of insoluble proteins and impaired intracellular protein homeostasis, which leads to ALS (Amado and Davidson, 2021). About 20% of fALS is known to be associated with SOD1 gene mutations, which were also the first ALS-related gene mutations to be discovered2. SOD1 gene mutation is a classical pathogenic model of ALS (Matsumoto et al., 2006; Kaur et al., 2016; van Zundert and Brown, 2017), and SOD1^G93A^ mice are the most commonly used mouse model of ALS. The phenotype of this model has the characteristics of early onset, fast disease progression and low variability, so it has become the workhorse for testing therapeutics aimed at ALS (De Giorgio et al., 2019).

Although sALS and fALS have heterogeneity in their clinical phenotype and pathogenesis, a common pathological feature is the abnormal aggregation of proteins inside or outside neurons to form inclusion bodies, which suggests that abnormal protein degradation plays an important role in the pathogenesis of ALS (Prudencio et al., 2009; Gois et al., 2020). Autophagy is an effective neuroprotective mechanism that actively promotes the clearance of pathogenic proteins, but in some cases, autophagy can become a target for the toxicity of these proteins (Zhao and Zhang, 2019; Assoni et al., 2021; Li et al., 2021). Studies have also shown that autophagy plays an important role in the pathogenesis of ALS (Maurel et al., 2018; Chua et al., 2022).

Rab small GTPases are the largest subfamily of Ras superfamily, and they play pivotal role in various membrane transport steps (Wennerberg et al., 2005; Kuchitsu and Fukuda, 2018). As an important member of Ras superfamily, Rab7 has been proved to play a key role in the fusion of autophagosomes and lysosomes, maintaining the balance of autophagy flux (Guerra and Bucci, 2016; Stroupe, 2018). In our previous work, we investigated Rab7 localization to membrane *via* immunofluorescence, and find that the amount of Rab7-positive structures of SOD1^G^93^A^ mice groups was less than WT (wild-type) mice groups significantly, but the level of total Rab7 protein was not significantly different among the groups. This finding suggests that Rab7 activation may be abnormal in SOD1^G^93^A^ mice (Bai et al., 2021).

In eukaryotic cells, the 15-carbon farnesyl lipid and the 20-carbon geranylgeranyl lipid which derived from cholesterol biosynthesis pathway intermediates are used to make post-translational modifications to C-terminal cysteine of target proteins, often referred to as prenylation. Protein prenylation is believed to be involved in fundamental cellular functions, such as membrane trafficking and signal transduction (Pereira-Leal et al., 2001). Rab7 needs to be activated by prenylation as well. Mevalonate (MVA) pathway is a critical pathway for the synthesis of isoprene compounds. The products of this pathway, such as farnesyl pyrophosphate (FPP) and geranyl pyrophosphate (GGPP), are necessary sources of protein prenylation. Some studies have suggested that blocking the MVA pathway can affect the prenylation of proteins, and then affect autophagy (Miettinen and Björklund, 2015; Tricarico et al., 2017). As inhibitors of HMG-CoA reductase, statins can significantly inhibit the production of MVA and its downstream metabolites (such as GGTP and FPP). In our previous work, simvastatin was employed to treat SOD1G93A mice (Bai et al., 2021). We found that simvastatin blocked autophagic flux and aggravated the abnormal accumulation of SOD1, which ultimately led to massive neuronal death. However, the increase of GGPP did not improve the autophagy flux disorders of SOD1G93A motor neurons (Qi et al., 2019). So we think about that the problem lies in the GGTaseII (namely RabGGTase), which is the prenoyltransferase of Rab GTPases. Therefore, we hypothesized that up-regulation of RabGGTase expression in the spinal anterior horn motor neurons of SOD1^G93A^ mice would improve the autophagy disorders in the motor neurons and protect neurons.

RabGGTase is formed by RabGGTA (α-subunit) and RabGGTB (β-subunit). In recent years, the role of prenylation of Rab protein in diseases has been extensively studied. For instance, Taheri et al. (2018) compared the expression of genes coding for the different subunits of proteins implicated in protein prenylation between patients with multiple sclerosis and healthy subjects, and found that RabGGTB was significantly downregulated in patients with total multiple sclerosis (Taheri et al., 2018). As an inhibitor of Rab-prenylation, psoromic acid has been found to be effective in the treatment of osteoporosis (Deraeve et al., 2012). Finally, no obvious correlation was found between expression of Rab prenylation pathway genes and the disease progression in choroideremia (Fry et al., 2021). However, to the best of our knowledge, there has been no research on the relationship between ALS and RabGGTase and its related treatment.

In the current study, we first demonstrated that RabGGTB expression was reduced in lumbar spinal motor neurons of SOD1^G93A^ mice, without finding data on RabGGTA reduction. Then a new method for the clinical treatment of ALS was developed. The onset of SOD1^G93A^ mice was significantly delayed and their survival time was prolonged by intrathecal injection of adeno-associated virus 9 (AAV9) carrying the human single chain RabGGTB gene. The overexpressed RabGGTB of spinal motor neurons in mice could protect motor neurons.

## 2. Materials and methods

### 2.1. Animals

SOD1^G93A^ mice (B6SJL-Tg [SOD1G93A]1Gur/J) were originally obtained from Jackson Laboratory (Bar Harbor, ME, United States). The right ear of the mice was marked with a metal ear tag. DNA was extracted from the left ear of hemizygous mice and genotyped using PCR. The mice were raised in constant temperature (22–24°C), constant humidity (40–60%), and 12 h light/dark alternating cycle, and fed with sterilized water and granular aseptic feed. All studies were performed in accordance with the guidelines for the Management of experimental animals formulated by the Ministry of Science and Technology of the China, and The Animal Ethics Committee of the Second Hospital of Hebei Medical University also approved the experimental procedures (Approval No. 2022-AE149). Throughout the course of the experiment, we tried our best to reduce the suffering of all animals.

SOD1-KO mice: Packaged AAV9-SaCas9-sgRNAs was injected into SOD1^G93A^ mice by intracerebroventricular (ICV) injection. Adeno-associated viruses carrying cas9 and sgRNA effectively delay the death of motor neurons by cutting out the SOD1 gene locus. The detail on SOD1-KO mice had been described in the study by Duan et al. (2020).

### 2.2. Adeno-associated virus production

Recombinant AAV particles were produced using the AAV Helper-Free System. First, the exogenous gene was cloned into a vector containing inverted terminal repeat/multiple cloning site. Inverted terminal repeat sequences in these vectors provide all the cis-acting elements necessary for AAV replication and packaging. Second, the recombinant expression plasmid was cotransfected into AAV-293 cells with pHelper (carrying adenovirus-derived genes) and pAAV-RC (carrying AAV replication and capsid genes), which provided trans-acting factors for AAV replication and packaging. A total of 2 or 3 days after transfection, the recombinant AAV was assembled in 293 T cells. Third, when collecting AAV particles from infected AAV-293 cells, AAV particles will generally be enriched in packaging cells, so most AAV particles can be recovered by collecting cells and then cleavage to release AAV particles into the supernatant. At the same time, the virus in the supernatant was concentrated and retained. Fourth, we concentrated and purified the virus supernatant of the third step. Finally, the titer of the virus was determined by quantitative analysis. The purified viral particles were stored at −80°C.

### 2.3. Injection

Intrathecal injection was performed by lumbar puncture. The intervention group was injected with AAV9-RabGGTB-GFP^+^ (10 μL/mice, 3.35 × 10^13^ vg/mL) and the other group was injected with AAV9-GFP^+^ (10 μL/mice, 3.56 × 10^13^ vg/mL). All mice were injected at postnatal day 40 (“P40”). During the operation, the head and upper limbs of the mice were covered with gauze by the researcher’s left hand, the skin of the lower spine of the mice was disinfected with alcohol cotton balls by the researcher’s right hand, and the central depression of the bilateral iliac spine line as the puncture point was marked with a marker. When the mice had been injected with 25 μL Hamilton injection syringe with a 27 g needle, the obvious tail flick showed that the needle tip had broken through the dura mater and the solution was injected slowly according to the 1 μL/min injection rate. After all the liquid had been injected, the needle was stopped for 2 min and then pulled out.

### 2.4. Assessment of behavior

In this experiment, body weight, footprint analysis, the accelerating rotarod test, and neurological deficits score were used to evaluate the motor function of mice. All SOD1^G93A^ mice were assessed from P80 to the end-stage. Body weight was measured twice a week between 10: 00 and 14: 00. The footprint analysis experiment was carried out in a runway 50 cm long and 5 cm wide. White paper was laid in the runway in advance, and the hind limbs of mice were stained with inks. We placed mice at one end of the runway, drove them to crawl forward, measured three consecutive footprints, and recorded the average stride length. The accelerating rotarod test was performed twice a week, and the experimental mice completed the learning and experimental stages 3 days before the start of the experiment. During the measurements, mice were placed on the rotating rod meter, starting at 2 rpm and reaching 30 rpm at 180 s. Each mouse was tested three times, each time at an interval of 20 min, and we recorded the longest time that the mouse stayed on the rotating rod instrument. The neurological deficits scoring criteria were as follows: 5 points for a healthy status without any symptoms of paralysis, 4 points for mild hindlimb paralysis and gait imbalance, 3 points for obvious gait imbalance and paralysis, 2 points for forelimb crawling or complete hindlimb paralysis, 1 point for complete hindlimb paralysis, mice obviously lying on their side, mice unable to straighten out after backbending, or mice had lost 20% of their body weight. The onset of the disease was defined as the point at which the full score was not achieved for the accelerating rotarod test or neurological function test, and the final time was defined as the loss of the righting reflex, whereby the mouse could not correct its body position within 30 s when placed sideways.

### 2.5. Histology

In our experiments, mice were typically sacrificed at 120 days. Mice were euthanized with pentobarbital sodium (150 mg/kg, i.p.). Once no response to pain stimulation was observed, mice were fixed on the operating table in the supine position and transcardially perfused by phosphate buffered saline (PBS). When the liver was bloodless, it was perfused with 4% frozen paraformaldehyde for 25 min. The spinal cord was removed and soaked in 4% paraformaldehyde overnight, and then equilibrated in 30% sucrose/PBS at 4°C. The spinal cord tissue was embedded in Tissue-Tek O.C.T., frozen in liquid nitrogen, and preserved at −80°C after O.C.T. solidification. We cut the spinal cord tissue using a microtome with a thickness of 15 μM.

### 2.6. Immunofluorescence staining

Frozen sections of lumbar spinal cords (L3-L5) of 120-day-old mice were stained using immunofluorescence. The Leica VT1000S vibratome was used to slice the embedded lumbar spinal cord; we then put the section in a moisturizing box and dried the slices away from light at room temperature for 30–45 min. Once slices were firmly attached to the slides, we put the moisturizing box into the ventilation cupboard, added 1 ml of 4% paraformaldehyde to each slice, fixed it for 10 min, then poured out the paraformaldehyde and washed it with PBS three times for 10 min each time. The sections were washed with 0.3% Triton X-100 PBS for 20 min and blocked with 10% goat serum for 1 h, then incubated with the following primary antibodies: GFP (1: 500 Abcam, ab13970), NeuN (1: 500,CST), GFAP (1: 500, CST, #3670 s), Iba1 (1: 500, Abcam, ab178847), Rab7 (1: 200, Abcam, ab137029), and SOD1 (1: 100, Abcam). The primary antibodies were incubated overnight at 4°C. Sections were washed in PBS and incubated with Alexa Fluor 488-conjugated Goat anti-Rabbit secondary antibody (1: 1000, Thermo Fisher, #A-11034), Alexa Fluor 594-conjugated Goat anti-Mouse secondary antibody (1: 1000, Thermo Fisher, #A-11032), and Alexa Fluor 647-conjugated Donkey anti-Goat secondary antibody (1: 1000, Thermo Fisher, #A211447) for 1 h at room temperature. Then, sections were washed in PBS again and the nucleus was stained with DAPI Fluoromount-G (Southern Biotech). The slides were observed using a fluorescence confocal microscope (LSM900, ZEISS, Germany). The imaging setup parameters remained constant throughout the process. The mean fluorescence intensity of RabGGTB, SOD1, GFAP, and IBA1 were analyzed using ImageJ. The number of NeuN+ cells and neuromuscular junctions (NMJs) were counted by a researcher blinded to the groups.

### 2.7. Statistical analyses

All data are reported as the mean ± SEM, and data analysis and cartography was performed using graph padprism8 Software. Kaplan–Meier survival analysis was used to compare the onset time and survival time between the SOD1^G93A^ mice injected with AAV9-RabGGTB-GFP^+^ group，injected with AAV9-GFP^+^ group and WT group. Unpaired *t*-tests were used to examine the differences between two groups. Comparisons between multiple groups were analyzed by one-way ANOVA. *p<0.05, **p<0.01, ***p<0.001; *p*<0.001, #*p*<0.05, ##*p*<0.01, ###*p*<0.001 were considered statistically significant.

## 3. Results

### 3.1. The reduction of RabGGTB expression in motor neurons of the lumbar spinal cord of SOD1^G93A^ mice

**Figure 1:**
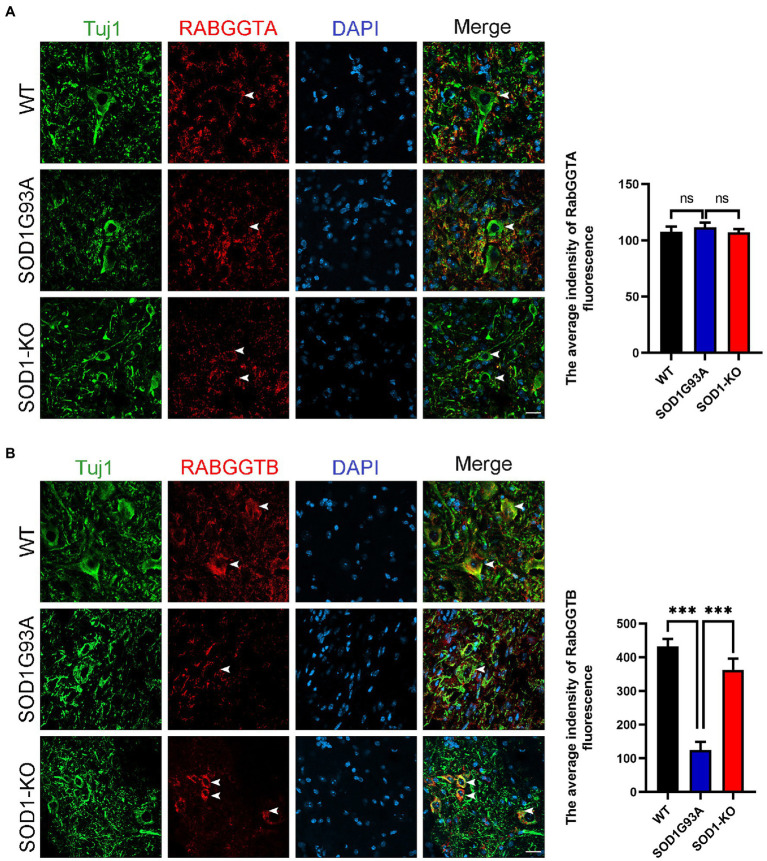
Expression of RabGGTA and RabGGTB in motor neurons of the lumbar spinal cord in different mice. **(A)** Immunofluorescence labeling of RabGGTA (red) in Tuj1-positive motor neurons (green) at 120 days (scale bars, 25 μM). Quantitative analysis of fluorescence intensity of RabGGTA staining. Compared with the SOD1^G93A^ group, neither the WT group nor SOD1-KO group, the fluorescence intensity of RabGGTA had statistical significance. **(B)** Immunofluorescence labeling of RabGGTB (red) in Tuj1-positive motor neurons (green) of the lumbar spinal cord at 120 days (scale bar, 25 μM). Quantitative analysis of fluorescence intensity of RabGGTB staining. Compared with the SOD1^G93A^ group, both the WT group and SOD1-KO group, the fluorescence intensity of RabGGTB had statistical significance. Data represent the mean ± SEM, *n* = 5 mice per group; *p*-values were determined using one-way ANOVA. Error bars denote the SEM. *p<0.05, **p<0.01, ***p<0.001. ns: no significance.

We first measured the expression of RabGGTA and RabGGTB in spinal cord anterior horn cells of WT and SOD1^G93A^ mice at 120 days. Five mice were observed in each group. RabGGTase (also called GGTaseII) consists of two subunits, RabGGTA and RabGGTB, which is one of the three prenyltransferases identified in mammals. RabGGTase exclusively modifies members of small GTPase, Rab family protains (Kuchay et al., 2019). The immunofluorescence results revealed that, in the anterior horn motor neurons of mice, RabGGTB was expressed at higher levels in WT mice and SOD1-KO mice than SOD1^G93A^ mice (Figure 1A). The SOD1-KO mice were SOD1^G93A^ mice modified with a mutant SOD1 gene by the AAV-SaCas9-sgRNA system, whereby the SOD1 gene was deleted and lifespan was improved, as confirmed in previous work (Duan et al., 2020). However, there was no significant difference in RabGGTA expression among the three genotypes of mice (Figure 1B). These results indicated that SOD1 gene mutation affected the expression of RabGGTB rather than RabGGTA in spinal motor neuron of SOD1^G93A^ mice.

### 3.2. Efficient overexpression RabGGTB in motor neurons through intrathecal injection of AAV9-RabGGTB-GFP^+^

**Figure 2:**
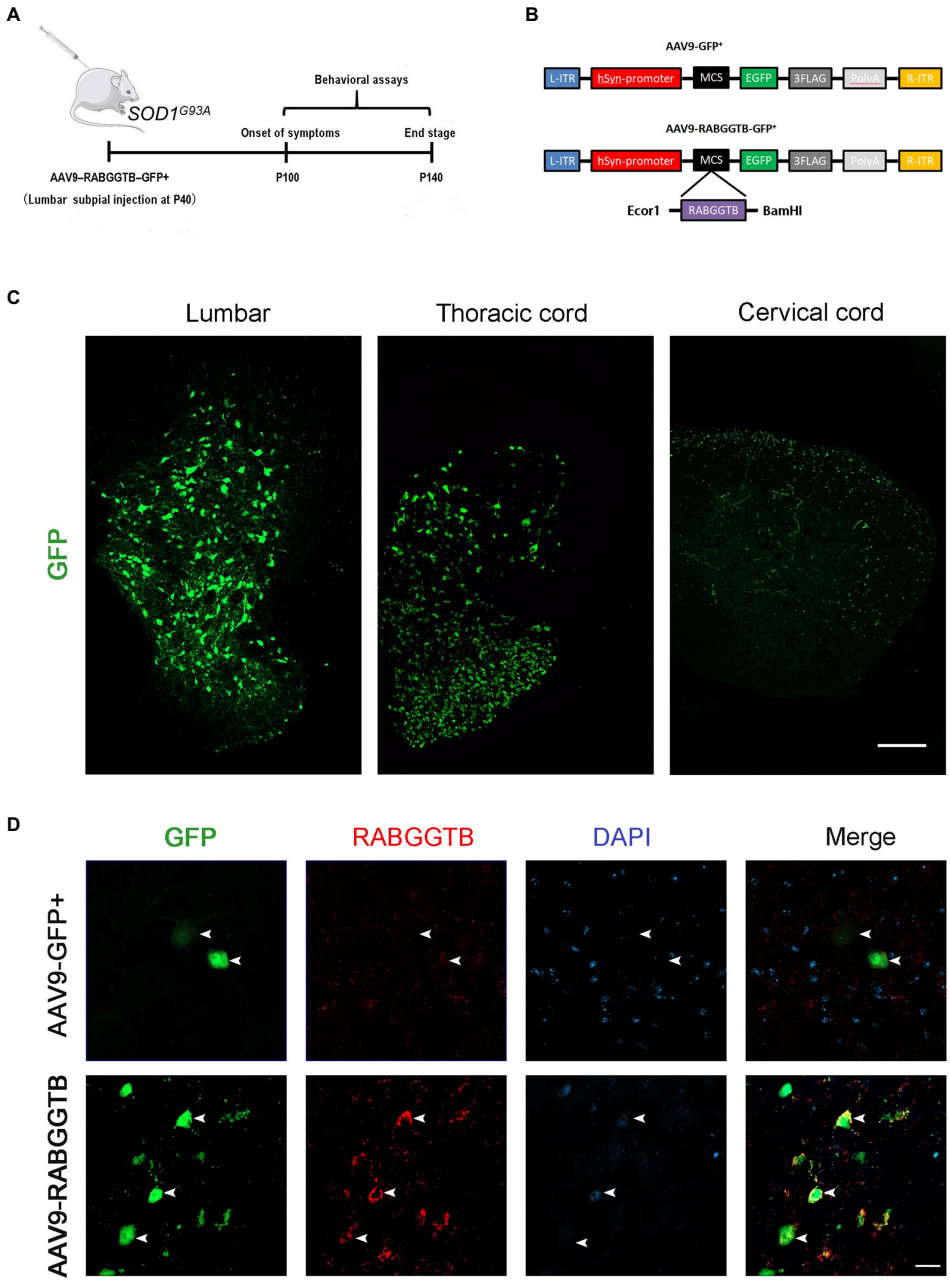
Efficient and specific GFP expression in motor neurons and efficient RabGGTB overexpression. **(A)** Schematic diagram of the experimental design. **(B)** The AAV9-RabGGTB-GFP^+^ and AAV9-GFP^+^ vector design is shown. **(C)** Representative immunofluorescence labeling of GFP (green) in the lumbar, thoracic and cervical regions of spinal cord (scale bar: 150 μM). **(D)** Immunofluorescence labeling of RabGGTB (red) in GFP-positive motor neurons (green) at 3 weeks after intrathecal injection with the virus (scale bars, 50 μM).

It has been shown that intrathecal injection of AAV was an effective method for CNS transfection, which had been confirmed in several studies (Bey et al., 2017; Leyton-Jaimes et al., 2019; Bravo-Hernandez et al., 2020). To test the ability of AAV9-RabGGTB-GFP^+^ to infect neurons in the spinal cord and evaluate its treatment effect, SOD1^G93A^ mice were injected intraspinally at P40 and observed until their sacrifice (Figure 2A). The vectors that carry the hSyn-promoter confine transgene expression specifically to neurons (Figure 2B). A subset of the mice (*n* = 5) were sacrificed 3 weeks after injection to assess the expression pattern of GFP and RabGGTB. We detected GFP-positive cells with strong signals in the lumbar and thoracic regions of spinal cord, but few in the cervical regions of spinal cord (Figure 2C). Next, we assessed the expression of RabGGTB in SOD1^G93A^ mice by intrathecal injection of AAV9-RabGGTB-GFP^+^ and AV9-GFP^+^. As expected, AAV9-RabGGTB-GFP^+^-infected neurons expressed RabGGTB more strongly than AAV9-GFP^+^-infected neurons (Figure 2D). Taken together, these results showed that intrathecal injection of AAV9-GFP^+^ and AAV9-RabGGTB-GFP^+^ resulted in massive infection in lumbar regions of spinal cord, and highly efficient overexpression of RabGGTB was obtained in the neurons of spinal cord.

### 3.3. Overexpression of RabGGTB increased recruitment of Rab7 to membranes and reduced the aggregation of SOD1 protein in the lumbar spinal cord of SOD1^G93A^ mice

**Figure 3:**
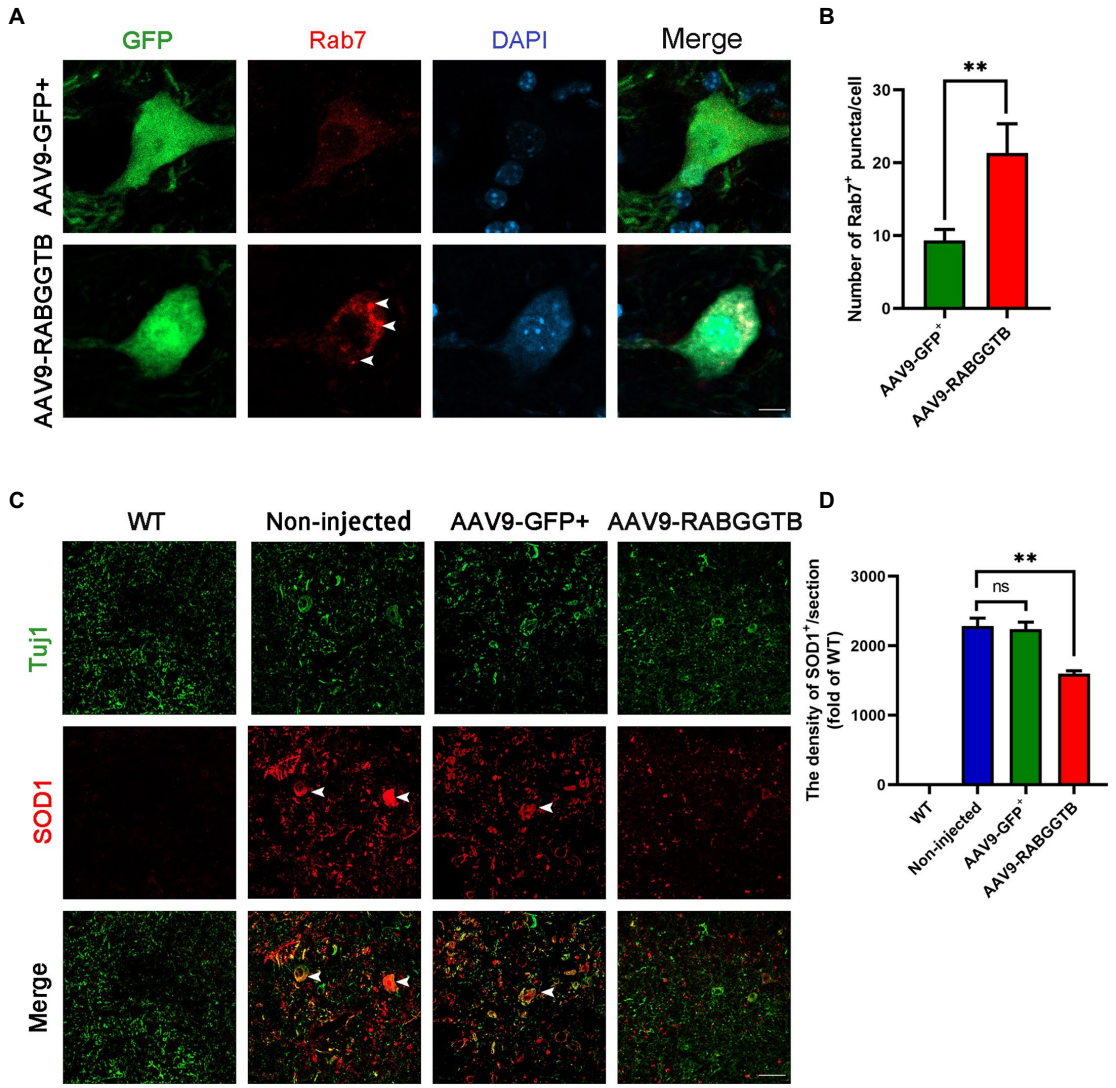
Overexpression of RabGGTB can increase the recruitment of Rab7 to membranes and reduced the abnormal aggregation of SOD1 protein. **(A)** Immunofluorescence labeling of Rab7 (red) in GFP-positive motor neurons (green) at 120 days (Scale bars, 10 μM). **(B)** Quantitative analysis of Rab7-positive puncta. **(C)** Representative micrographs of lumbar spinal cord sections with WT, non-injected, and AAV9-GFP^+^-treated SOD1^G93A^ mice, and AAV9-RabGGTB-GFP^+^-treated SOD1^G93A^ mice. Immunofluorescence staining of SOD1 in the lumbar spinal cord at 120 days (scale bars, 50 μM). **(D)** Quantification of relative fluorescence intensity of SOD1 in the lumbar medulla of WT mice, non-injected SOD1^G93A^ mice, SOD1^G93A^ mice injected with AAV9-GFP^+^, and SOD1^G93A^ mice injected with AAV9-RabGGTB-GFP^+^. Data represent the mean ± SEM, *n* = 5 mice per group; *p*-values were determined using one-way ANOVA. Error bars denote the SEM. *p<0.05, **p<0.01, ***p<0.001. ns: no significance.

It is generally believed that Rab7 plays an important role in late autophagy and is an important regulator of membrane transport. So we detected the localization of Rab7 on the membrane by immunofluorescence. We found that the amount of Rab7-positive structures increased significantly in MNs after injected with AAV9-RabGGTB-GFP^+^ (Figures 3A,B). As we all know, abnormal accumulation of SOD1 protein can produce cytotoxicity and toxic effects on motor neurons, and that this accumulation is involved in the pathological process of ALS (Abu-Hamad et al., 2017). Our previous research showed that, in SOD1^G93A^ mice, the membrane localization process of Rab7 is inhibited, and the aggregation of misfolded SOD1 proteins is increased21. In this study, we observed the expression of SOD1 in the lumbar spinal cord of each group of mice at 120 days. Five mice were observed in every group. Immunofluorescence showed that the abnormal aggregation of SOD1 in the lumbar spinal cord of mice injected intrathecally with AAV9-RabGGTB-GFP^+^ was reduced compared with the group injected with AAV9-GFP^+^ (Figures 3C,D). Our results suggest that overexpression of RabGGTB in the lumbar spinal cord of SOD1^G93A^ mice can increase the localization of Rab7 on the membranes and reduce the abnormal aggregation of SOD1.

### 3.4. Overxpression of RabGGTB protected motor neurons and reduced activation of glia cells in spinal cord of SOD1^G93A^ mice

**Figure 4:**
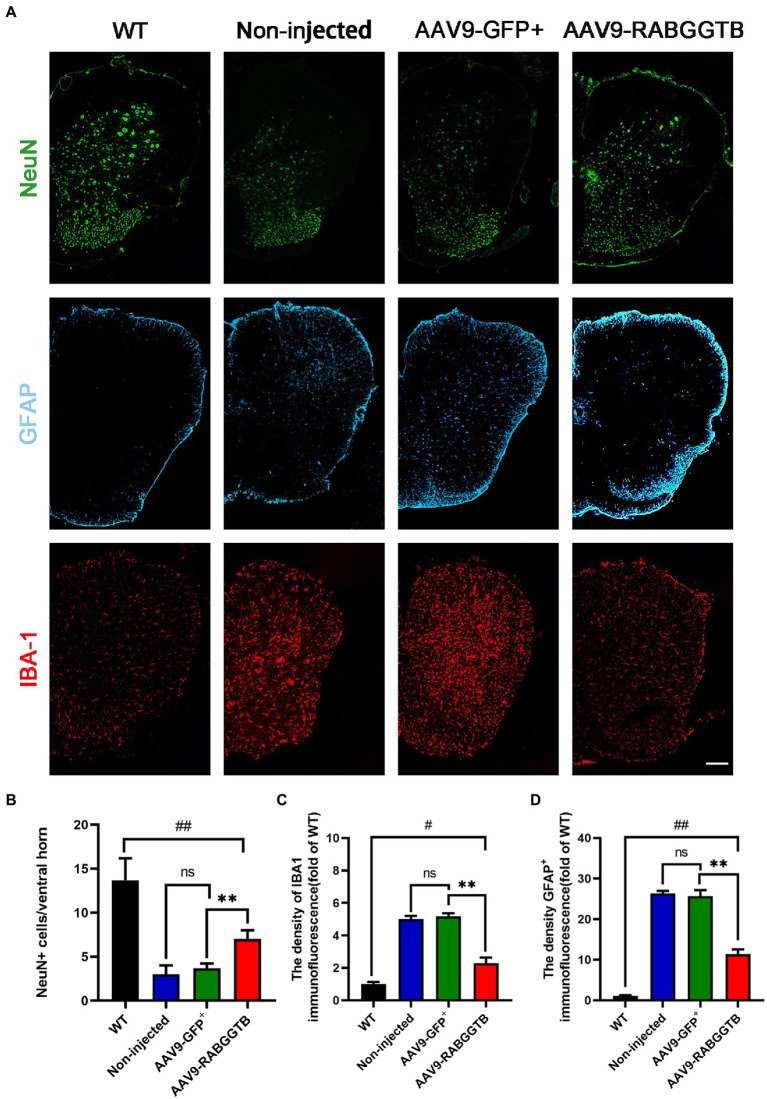
Expression of RabGGTB in the lumbar spinal cord protected motor neurons and had an effect on the overactivation of astrocytes and microglia in SOD1^G93A^ mice. **(A)** Immunofluorescence staining of Neun, GFAP, and Iba1 in the lumbar spinal cord at 120 days (scale bars, 150 μM). **(B)** At day 120, Neun-positive motor neurons in the anterior horn of the spinal cord were counted in each group (*n* = 3 mice per group). **(C)** Quantification the intensity of GFAP-positive cells in the lumbar spinal cord (*n* = 3 mice per group). **(D)** Quantification of the intensity of IBA1-positive cells in the lumbar spinal cord. Data represent the mean ± SEM, *n* = 3 mice per group; *p*-values were determined using one-way ANOVA. Error bars denote the SEM. *p<0.05, **p<0.01, ***p<0.001, #*p*<0.05, ##*p*<0.01, ###*p*<0.001. ns: no significance.

The main pathological changes of the spinal cord in ALS are degeneration and loss of motor neurons, as well as the proliferation of associated glial cells. We quantified the number of motor neurons in lumbar spinal cord sections from each group (*n* = 3) using immunofluorescence staining at 120 days. Motor neurons were defined as NeuN-positive cells with a diameter of more than 25 μm, with visible nucleoli and located in the anterior horn of the spinal cord. Immunofluorescence staining results revealed that, compared with SOD1^G93A^ mice that overexpressed RabGGTB, untreated mice showed massive motor neuron loss in the anterior horn of the spinal cord (Figures 4A,B). We next observed the activation of glial cells (*n* = 3 for each group). We found that both GFAP-positive astrocytes and Iba1-positive microglia in the lumbar spinal cord were reduced in SOD1^G93A^ mice injected with AAV9-RabGGTB-GFP^+^ at day 120 (Figures 4A,C,D). However there was no significant difference in non-injected mice and AAV9-GFP^+^ group.

### 3.5. Overexpression of RabGGTB rescued defect of neuromuscular junctions

**Figure 5:**
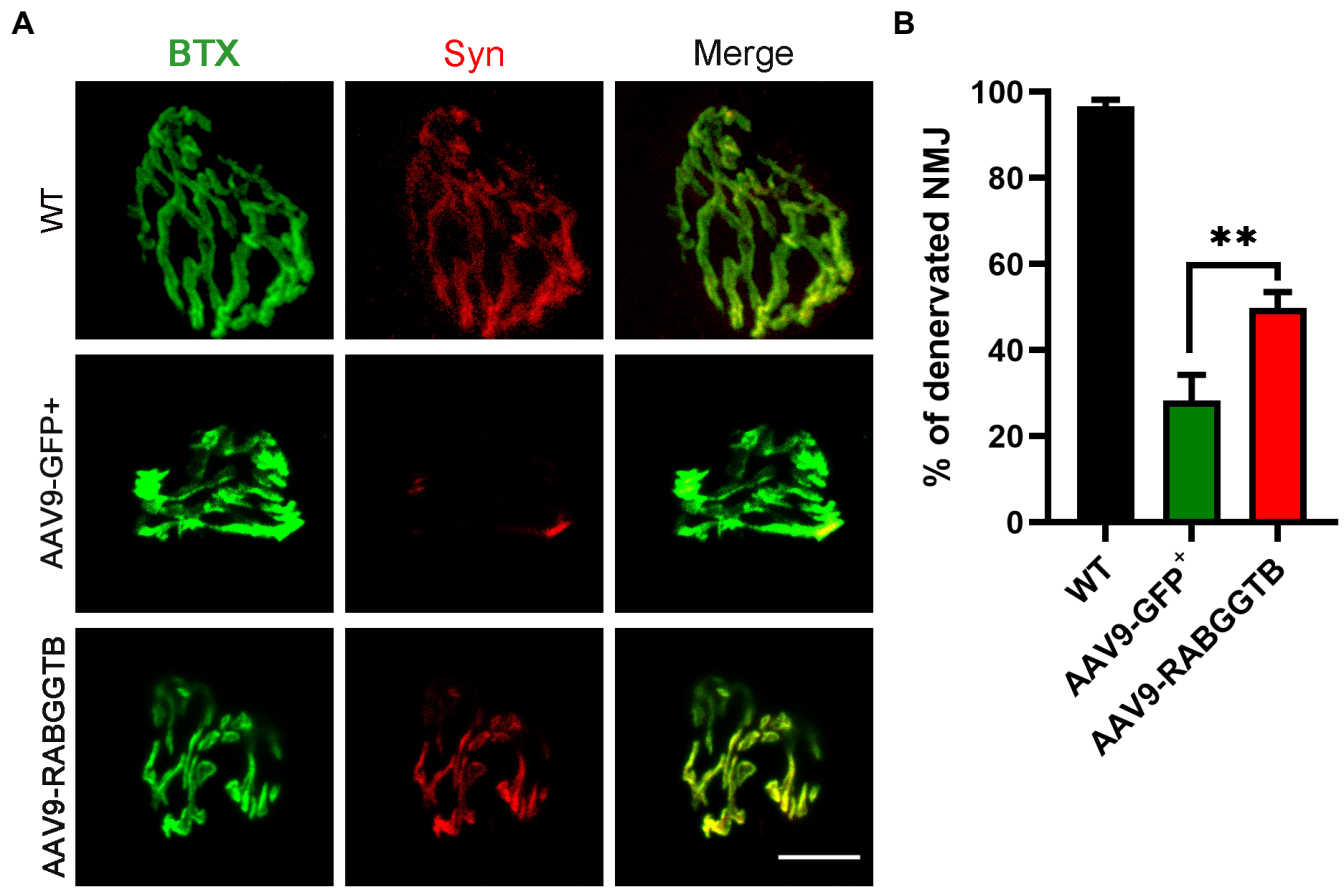
Overexpression of RabGGTB rescues neuromuscular junctions of the gastrocnemius muscle in SOD1^G93A^ mice. **(A)** Immunofluorescence staining the NMJ of the gastrocnemius muscle in P120 SOD1^G93A^ mice labeled with α-cyclopentotoxin (red) and synaptophysin-positive presynaptic endings (green; scale bars, 5 μM). **(B)** Quantification of the percentage of total innervated NMJ in the gastrocnemius muscle of mice at P120. The gastrocnemius section was 15 μM deep, and each section counted three fields of view with total of 60 α-bungarotoxin staining endplates. According to the co-staining with the Syn marker, endplates were identified as denervated or not. Data represent the mean ± SEM, *n* = 3 mice per group; *p*-values were determined using *t*-tests. Error bars denote the SEM. *p<0.05, **p<0.01, ***p<0.001. NMJ: neuromuscular junction.

ALS is a fatal neuromuscular disease characterized by progressive motor neuron loss and skeletal muscle atrophy. Denervation is an important pathological event leading to paralysis in ALS. We analyzed denervation of the gastrocnemius neuromuscular junction in SOD1^G93A^ mice and compared this with the different groups. We stained the acetylcholine receptor of the motor endplates with alpha-bungarotoxin, and colocalization with syntrophin-1 was used to quantify NMJ occupancy (Figure 5A), 3 mice were observed in both group. The results showed that motor endplate innervation in SOD1^G93A^ mice injected with AAV-GFP^+^ (28 ± 6%) was significantly less than that in WT mice and also less than that in mice injected with AAV9-RabGGTB-GFP^+^ (49 ± 3%; Figure 5B). These results indicate that RabGGTB overexpression in the spinal cord has a certain protective effect on NMJs.

### 3.6. Intrathecal injection of AAV9-RabGGTB-GFP^+^ in SOD1^G93A^ mice prolonged disease phenotype onset

**Figure 6:**
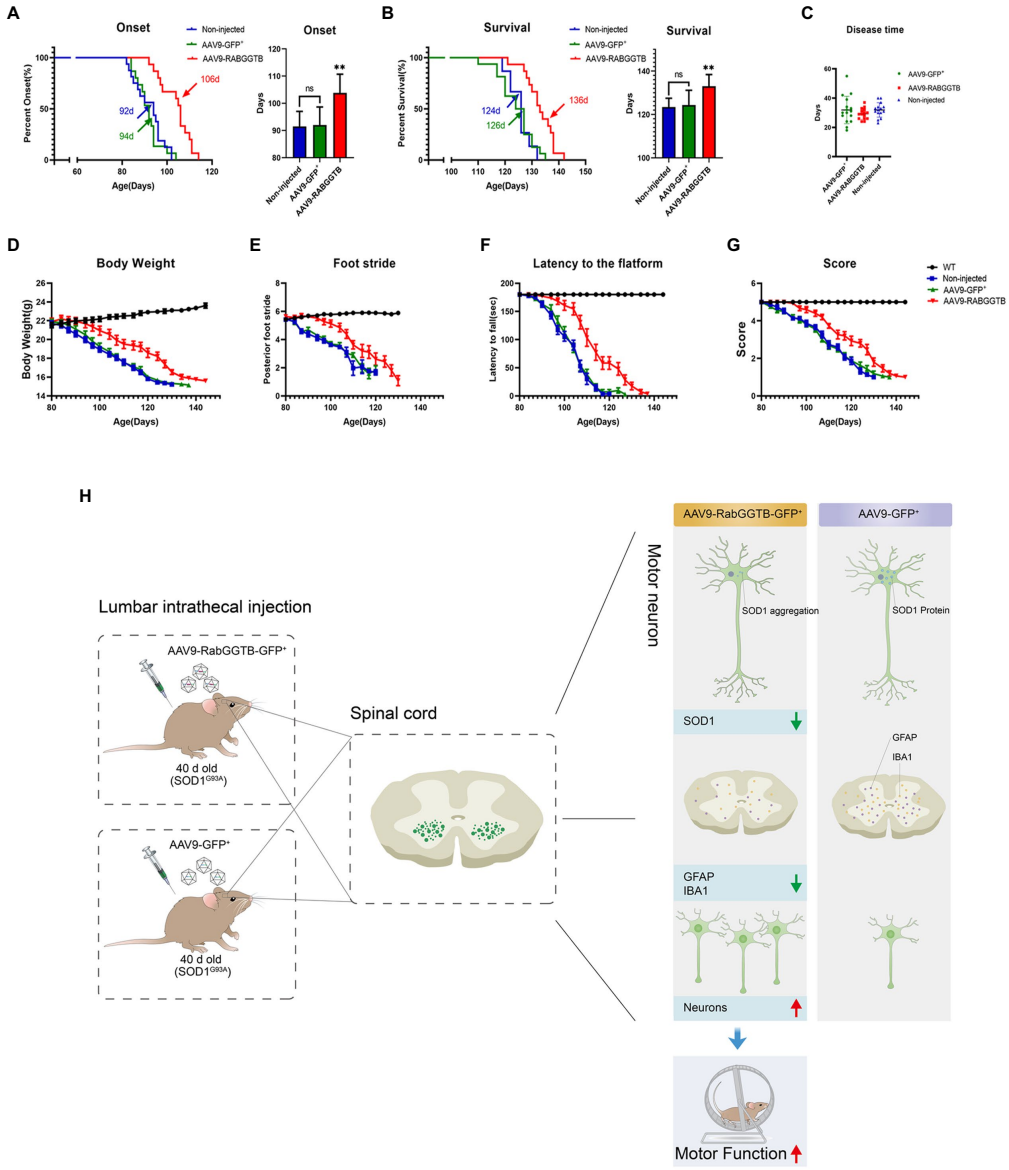
Overexpression of RabGGTB in the spinal cord of mice delayed disease onset, extended lifespan, and improved the disease phenotype. Mice were given intrathecal injection of AAV on P40, and behavioral changes were observed on P80. **(A)** The median disease onset of the treated mice was significantly delayed (non-injected: 92 days; AAV9-GFP^+^: 94 days; AAV9-RabGGTB-GFP^+^: 106 days; *p* < 0.01). **(B)** The median survival time of the treated mice was also significantly prolonged (non-injected: 124 days; AAV9-GFP^+^: 126 days; AAV9-RABGGTB-GFP^+^: 136 days; *p* < 0.01). **(C)** However, overexpression of RabGGTB did not prolong the course of disease in mice (non-injected: 32 days; AAV9-GFP^+^: 31 days; AAV9-RabGGTB-GFP^+^: 30 days; *p* > 0.05). **(D)** The body weight curves of WT and SOD1^G93A^ mice non-injected or injected with AAV9-GFP^+^ or AAV9-RabGGTB-GFP^+^. RabGGTB overexpression delayed the decrease of body weight in mice. **(E–G)** The SOD1^G93A^ mice overexpressing RabGGTB performed better on rotation tests, gait analysis tests, and neurological function evaluation compared with their untreated littermates. **(H)** Schematic diagrams of the results. The onset time was measured using the rotarod test. The end-stage was defined as the loss of the righting reflex, whereby a mouse cannot correct its body position within 30 s when placed on its side. Data represent the mean ± SEM, *n* = 15 or 16 mice per group; *p*-values were determined using one-way ANOVA, Error bars denote the SEM. *p<0.05, **p<0.01, ***p<0.001. ns: no significance.

Next, we assessed the effects of overexpression of RabGGTB on suivival of SOD1^G93A^ mice. SOD1^G93A^ mice injected with AAV9-RabGGTB-GFP^+^ (*n* = 15) and SOD1^G93A^ mice injected with AAV9-GFP^+^ (*n* = 16) were injected intrathecally at day 40 after birth, and their disease phenotype was observed at the same time as those of non-injected mice (*n* = 15) and WT mice (*n* = 16) until the end stage of their life. Disease onset (measured by the accelerated stick test or the neurological function score) was significantly delayed by injection with AAV-RabGGTB-GFP^+^, compared with the mice injected with AAV9-GFP^+^ and non-injected (non-injected group: 92 days; injected with AAV9-GFP^+^ group: 94 days; injected with AAV9-RabGGTB-GFP^+^ group: 106 days; *p* < 0.01; Figure 6A). Correspondingly, RabGGTB overexpression also significantly prolonged the life span of SOD1^G93A^ mice (non-injected group: 124 days; injected with AAV9-GFP^+^ group: 126 days; injected with AAV9-RabGGTB-GFP^+^ group: 136 days; *p* < 0.01; Figure 6B), but did not prolong the disease course (non-injected group: 32 days; injected with AAV9-GFP^+^ group: 31 days; injected with AAV9-RabGGTB-GFP^+^ group: 30 days; *p* > 0.05; Figure 6C). As one of the most important symptoms used to assess the progression of ALS, the weight loss of mice injected with AAV9-RabGGTB-GFP^+^ was also significantly delayed (Figure 6D). Furthermore, compared with the mice injected with AAV9-GFP^+^ littermates, the mice injected with AAV9-RabGGTB-GFP^+^ performed better on the rotation tests, gait analysis tests, and neurological function scores (Figures 6E–G). These results indicated that the decline of motor function was delayed in the group injected with AAV9-RabGGTB-GFP^+^ compared with the AAV9-GFP^+^ and the non-injected group. But there was no difference in disease onset, life span and behavior phenotype between the AAV9-GFP^+^ and non-injected group. This also showed that intrathecal injection did not damage the motor function of mice; our injection was safe and reliable in the animals.

## 4. Discussion

The first gene mutation found to be associated with ALS disease is that of the SOD1 gene (Cleveland and Liu, 2000). So far, 146 mutations in SOD1 have been identified in patients with fALS (Brown et al., 2021). Abnormally expressed SOD1 can produce toxicity that causes oxidative stress and oxidative damage, and can affect the structure and function of neurons. However, the specific pathological process and toxic effects on motor neurons remain to be further explored. It is generally believed that abnormal aggregation of the SOD1 protein is one of the primary causes of motor neuron death in SOD1-related ALS cases. In fact, in more than 20% of fALS and 4% of sALS cases, it is possible to detect point mutations and small fragment deletions of the SOD1 gene (Turner et al., 2013; Marangi and Traynor, 2015).

Therefore, regulating the abnormal expression of SOD1 mutant protein or increasing its degradation rate has become the goal of many therapeutic ALS studies (van Zundert and Brown, 2017; Bravo-Hernandez et al., 2020; Duan et al., 2020). Autophagy is a biological process in which cells phagocytose and degrade their proteins and organelles (Xiang et al., 2019; Zhao and Zhang, 2019). Recent studies have shown that autophagy is one of the early pathological events of motor neuron degeneration and death, and may be involved in the pathogenesis of ALS (Rudnick et al., 2017). Based on our previous research, we hypothesized that up-regulation of RabGGTase expression in the spinal anterior horn motor neurons of SOD1^G93A^ mice would improve the autophagy disorders in the motor neurons of SOD1^G93A^ mice and protect the neurons.

We used AAV as a vector for gene regulation. AAV is one of the most commonly used vectors for gene therapy. It is a parvovirus with a single-stranded DNA genome, which has the characteristics of high safety, low immunogenicity, long expression time, and stable transfection. It is the safest viral vector approved by FDA and can be directly used in human gene therapy (Hinderer et al., 2018; Huang et al., 2021). AAV has been widely used in a number of basic research studies and clinical applications associated with ALS (Yamashita et al., 2013; Thomsen et al., 2017; Leyton-Jaimes et al., 2019; Bravo-Hernandez et al., 2020). Among them, the choice of intervention mode and the type of virus are two important factors that determine the effect of the treatment. Mariana et al., used intrathecal injection of AAV9 shRNA silencing mutant SOD1 before (P120) and after disease onset (P384), respectively, compared with the non-injected mice, so that the survival rate of SOD1^G37R^ mice was significantly improved (Bravo-Hernandez et al., 2020). Marcel et al., overexpressed macrophage migration inhibitory factor (MIF) in the spinal cord of mice by intrathecal injection of AAV vector on P10 to inhibit the accumulation of misfolded SOD1 protein and prolong the survival time of mutant SOD1 mice (Leyton-Jaimes et al., 2019). Recent studies have shown that satisfactory transfection results have been achieved by injecting AAV into the lateral ventricle of the pups. Bey et al. (2017) suggested that the transfection effect of intrathecal injection was better than that of intracerebroventricular injection in adult mice.

When the protein is translated, isoprene modification begins and cannot be reversed (McTaggart, 2006). Prenylation of proteins is catalyzed by FTase and GGTase (GGTaseI and GGTase II). Both FTase and GGTase are heterodimeric enzyme complexes composed of α and β subunits (Benetka et al., 2006). FTase and GGTaseI recognize proteins with a C-terminal CaaX motif, where C stands for cysteine, A for aliphatic amino acids, and X for arbitrary amino acids (Guo et al., 2008). The X residue determines whether the protein is farniated or geranylgeranylated. Another prenyltransferase, GGTase II, which is formed by RabGGTA and RabGGTB, is mainly involved in the modification process of isoprene of Rab GTP protein. However, we know very little about how these enzymes work (Konstantinopoulos et al., 2007; Berndt et al., 2011; Kuchay et al., 2019). In the present experiment, we first verified the difference in the expression of two subunits of RabGGTase in the motor neurons of the lumbar spinal cord of SOD1^G93A^ mice; compared with WT mice, the expression of β subunits in motor neurons decreased rather than that of α subunits. However, the molecular details of prenylation remain unclear, the reason for the reduction of the β subunit needs to be further explored.

Our result show that in the mice injected with AAV9-RabGGTB-GFP^+^, compared with the mice injected with AAV9-GFP^+^ and non-injected, the SOD1 protein in motor neurons degraded significantly, the number of motor neuron cells increased, the gliois of spinal cord decreased, which improved the impair motor function (Figure 6H). And more importantly, our result also show that the expression of RabGGTB *in vivo* could delay the disease onset and prolong the life span of the SOD1 mouse model, but did not prolong the course of the disease in mice. This may be because RabGGTB spreads only within a few millimeters of the injection site in the nerve tissue of AAV, so that there is almost no overexpression of RabGGTB in the motor neurons of the medulla oblongata and cervical spinal cord innervating the respiratory muscle. At the same time, the absence of prolonged disease may also be related to the rapid loss of anterior horn motoneurons after the onset of SOD1^G93A^ mice, accompanied by progressive limb weakness and eventual rapid death. The behavioral changes in mice were accompanied by a decrease in the accumulation of mutant SOD1 in the spinal cord and a reduction in overactivation of microglia and astrocytes.

In conclusion, overexpression of RabGGTase β subunit-RabGGTB in the spinal cord of SOD1^G93A^ mice inhibited the aggregation of mutant SOD1 protein，which could be caused by Rab7-mediated activation of autophagy, thereby protecting neurons and improving disease phenotype. Enhanced autophagy could be achieved by activated Rab7 to promote the fusion of autophagosomes and lysosomes. In the next, we will focus on the alteration of autophagy and its related mechanism in motor neurons of ALS mouse model. In conclusion, we believe that upregulation of RabGGTase in the motor neurons of spinal cord may have important therapeutic significance for ALS.

## Data availability statement

The original contributions presented in the study are included in the article/Supplementary material, further inquiries can be directed to the corresponding authors.

## Ethics statement

The animal study was reviewed and approved by the Ministry of Science and Technology of the People’s Republic of China and the Animal Ethics Committee of the Second Hospital of Hebei Medical University also approved the experimental procedures (Approval No. 2022-AE149).

## Author contributions

RL and YL conceptualized and designed the experiments, and reviewed and edited the manuscript. TG, JH, CX, JY, QL, and HD performed all the experiments. TG, RL, and YL analyzed and interpreted the data. TG wrote the manuscript. All authors contributed to the article and approved the submitted version.

## Funding

This work was supported by the Natural Science Foundation of Hebei Province H2021206310 and the Key Project of Technical Health Research and Achievement Transformation of Hebei Provincial Department of Health zh2018004 and Training Project for Professional Leaders of Hebei Provincial Department of Finance.

## Conflict of interest

The authors declare that the research was conducted in the absence of any commercial or financial relationships that could be construed as a potential conflict of interest.

## Publisher’s note

All claims expressed in this article are solely those of the authors and do not necessarily represent those of their affiliated organizations, or those of the publisher, the editors and the reviewers. Any product that may be evaluated in this article, or claim that may be made by its manufacturer, is not guaranteed or endorsed by the publisher.
