# Supplementary figures and images for: Protective effects of intrathecal injection of AAV9-RabGGTB-GFP+ in SOD1G93A mice
Source: Front Aging Neurosci. 2023 Mar 14;15:1092607. doi: 10.3389/fnagi.2023.1092607 (PMC10036913; doi:10.3389/fnagi.2023.1092607)

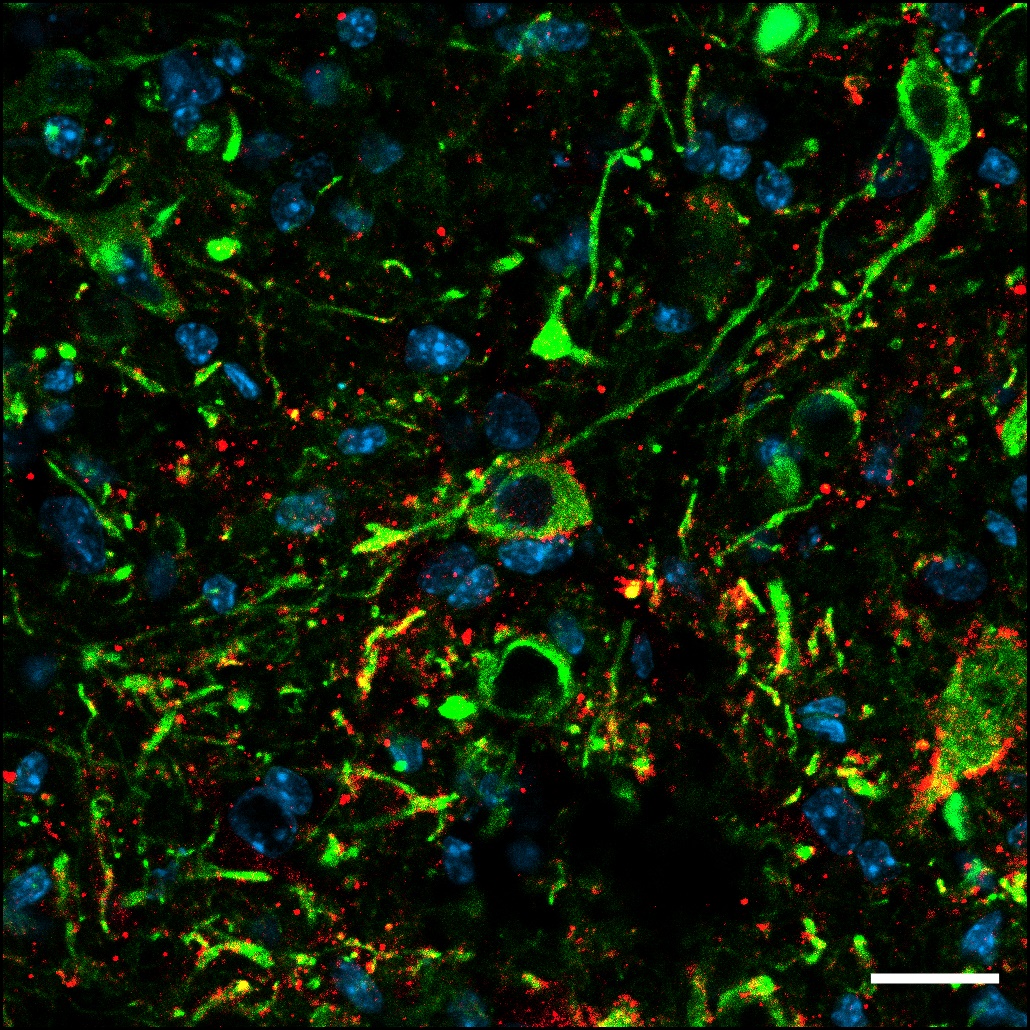

Supplement: Supplementary file 1 [file Data_Sheet_2.ZIP › Fig.1/the expression of RabGGTA in SOD1 mice 1.jpg]

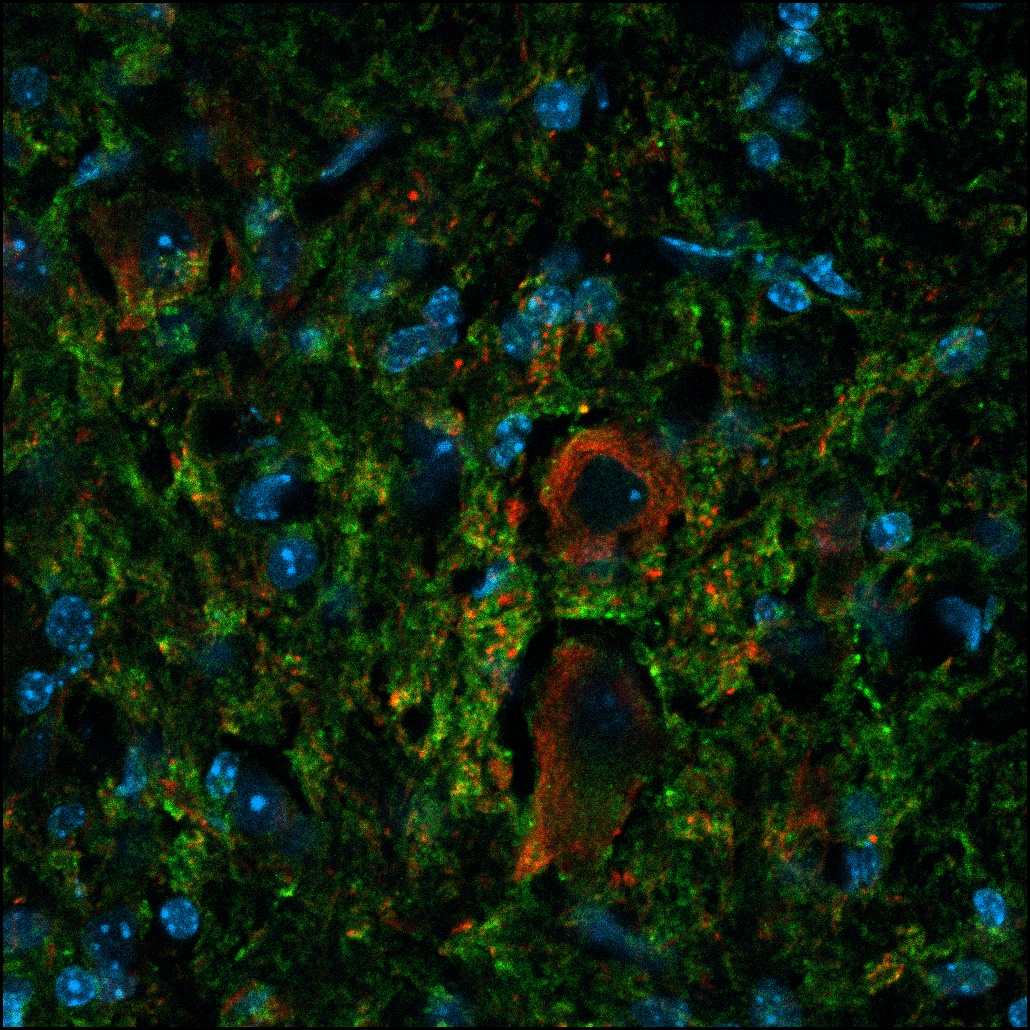

Supplement: Supplementary file 1 [file Data_Sheet_2.ZIP › Fig.1/the expression of RabGGTA in SOD1 mice.jpg]

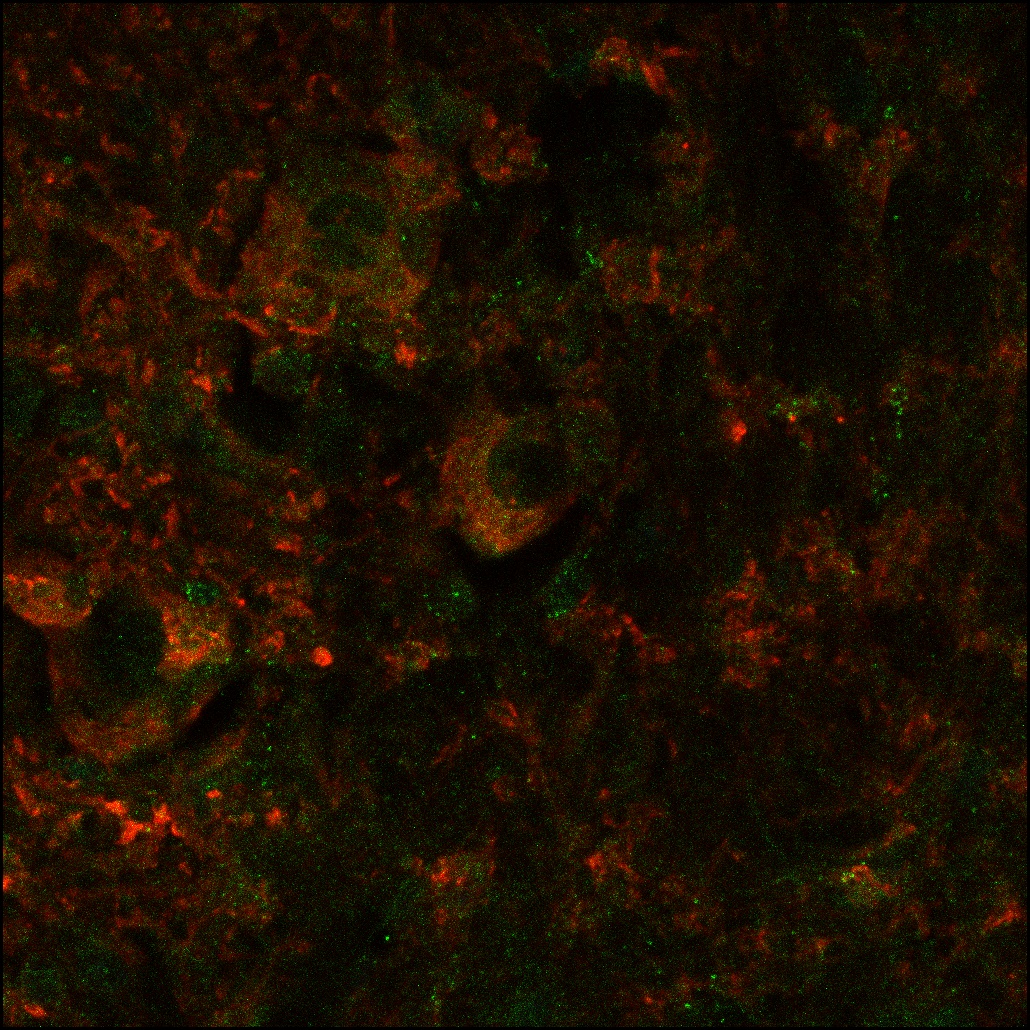

Supplement: Supplementary file 1 [file Data_Sheet_2.ZIP › Fig.1/the expression of RabGGTA in SOD1-KO mice.jpg]

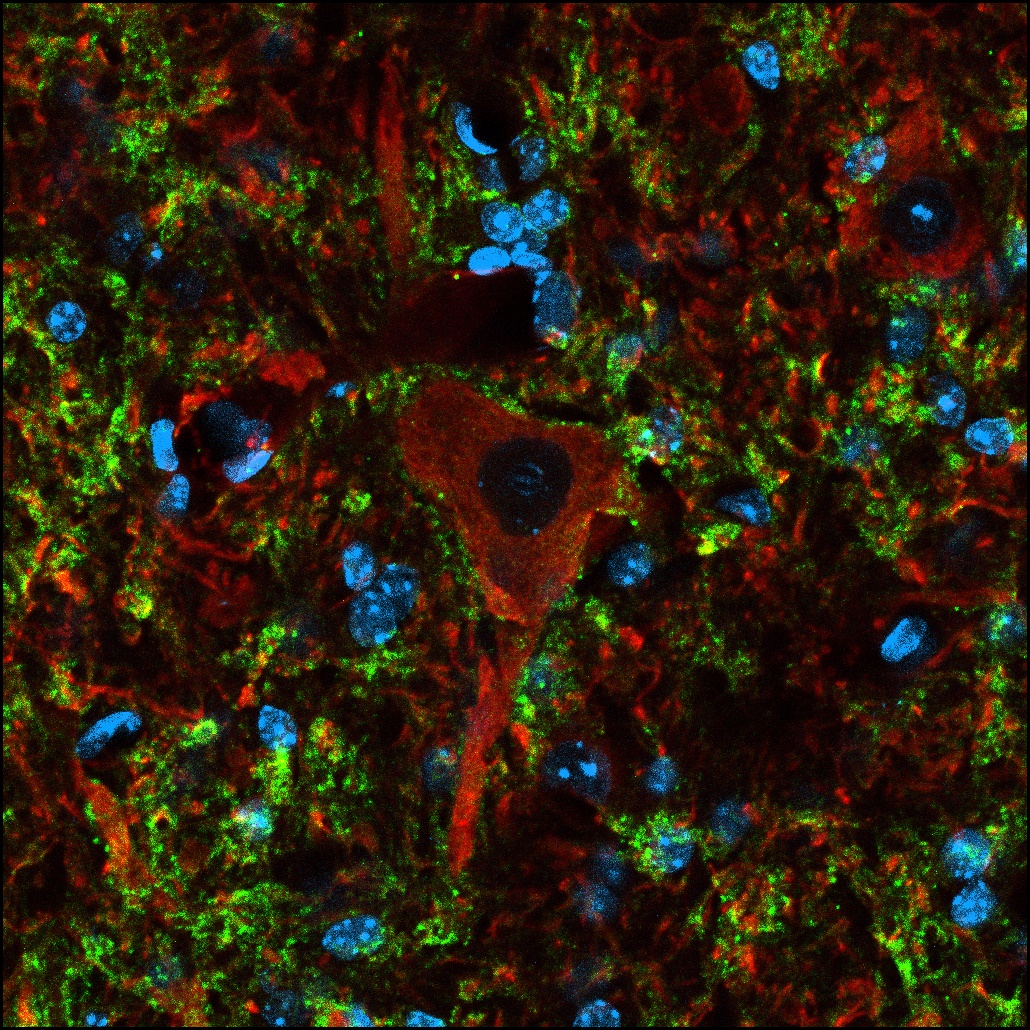

Supplement: Supplementary file 1 [file Data_Sheet_2.ZIP › Fig.1/the expression of RabGGTA in WT mice.jpg]

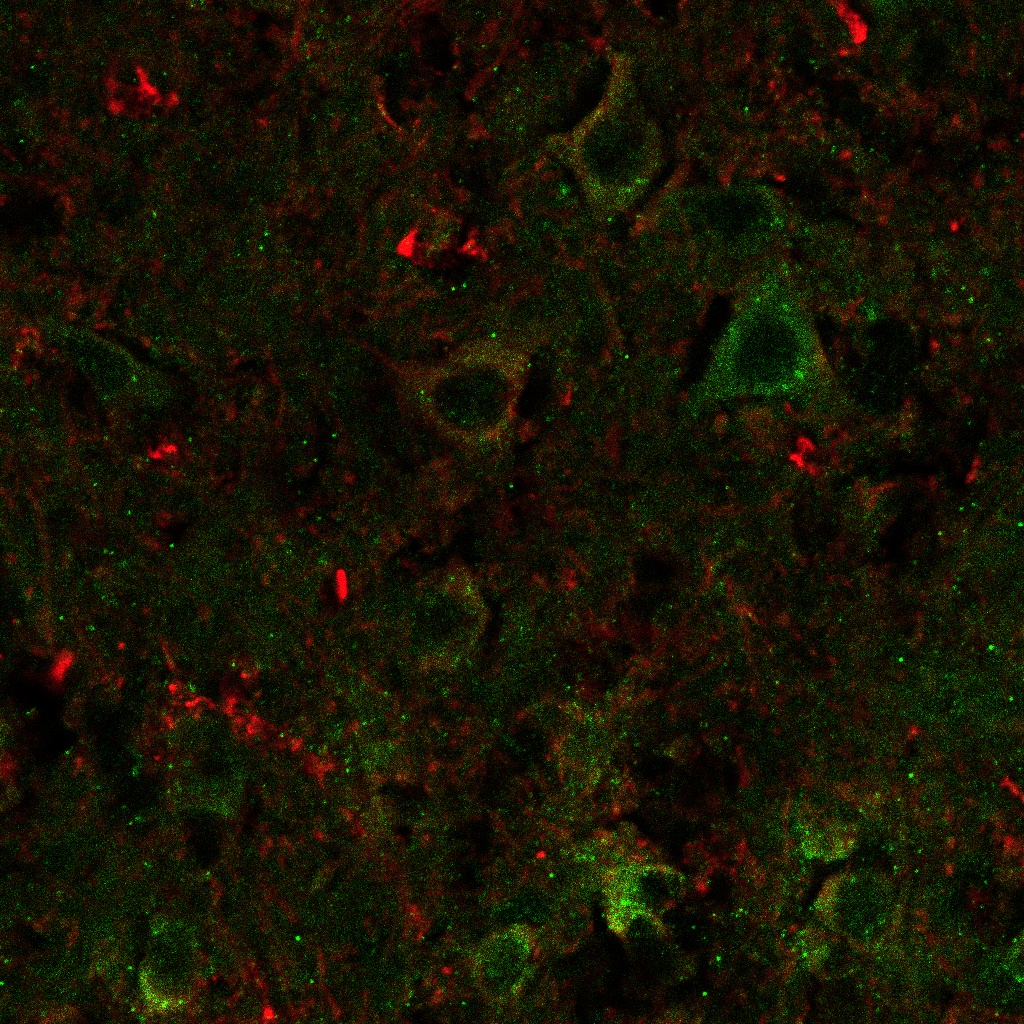

Supplement: Supplementary file 1 [file Data_Sheet_2.ZIP › Fig.1/the expression of RabGGTB in SOD1 mice 1.jpg]

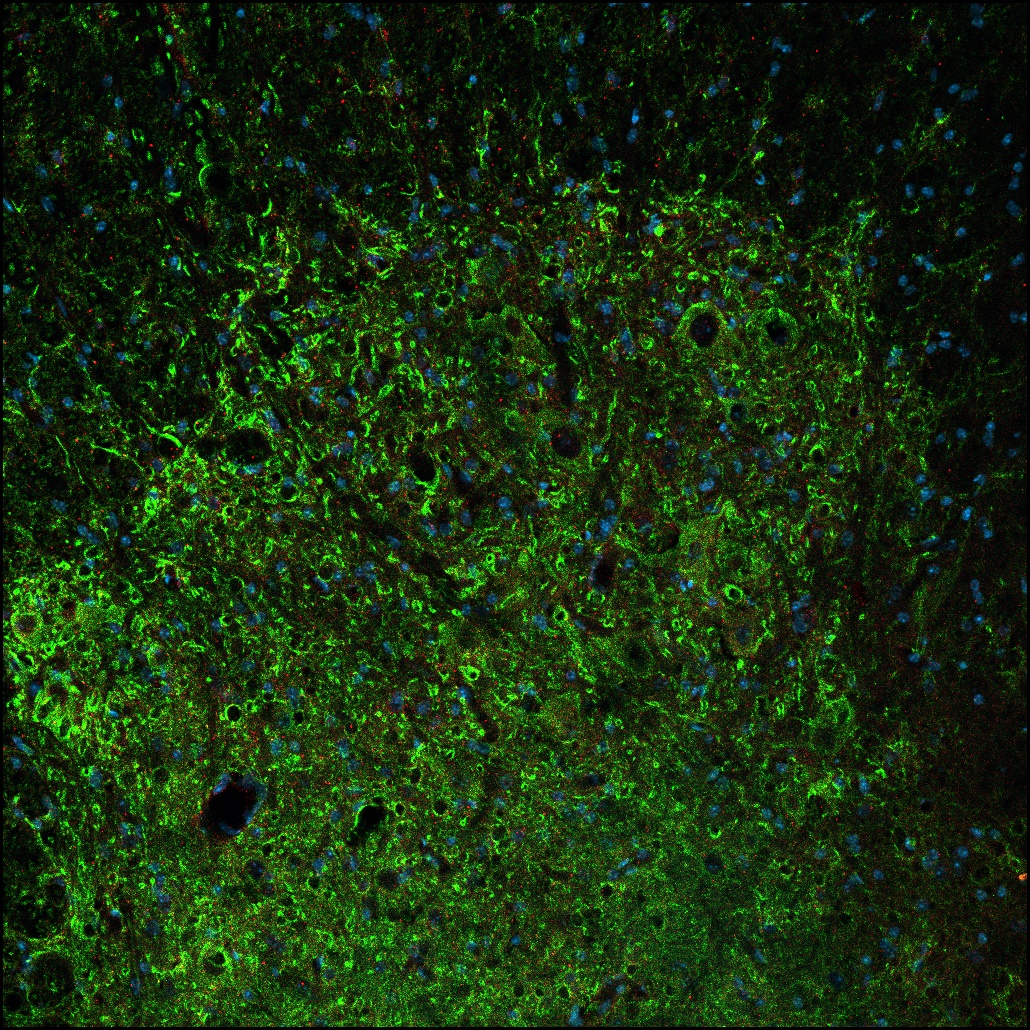

Supplement: Supplementary file 1 [file Data_Sheet_2.ZIP › Fig.1/the expression of RabGGTB in SOD1 mice 2.jpg]

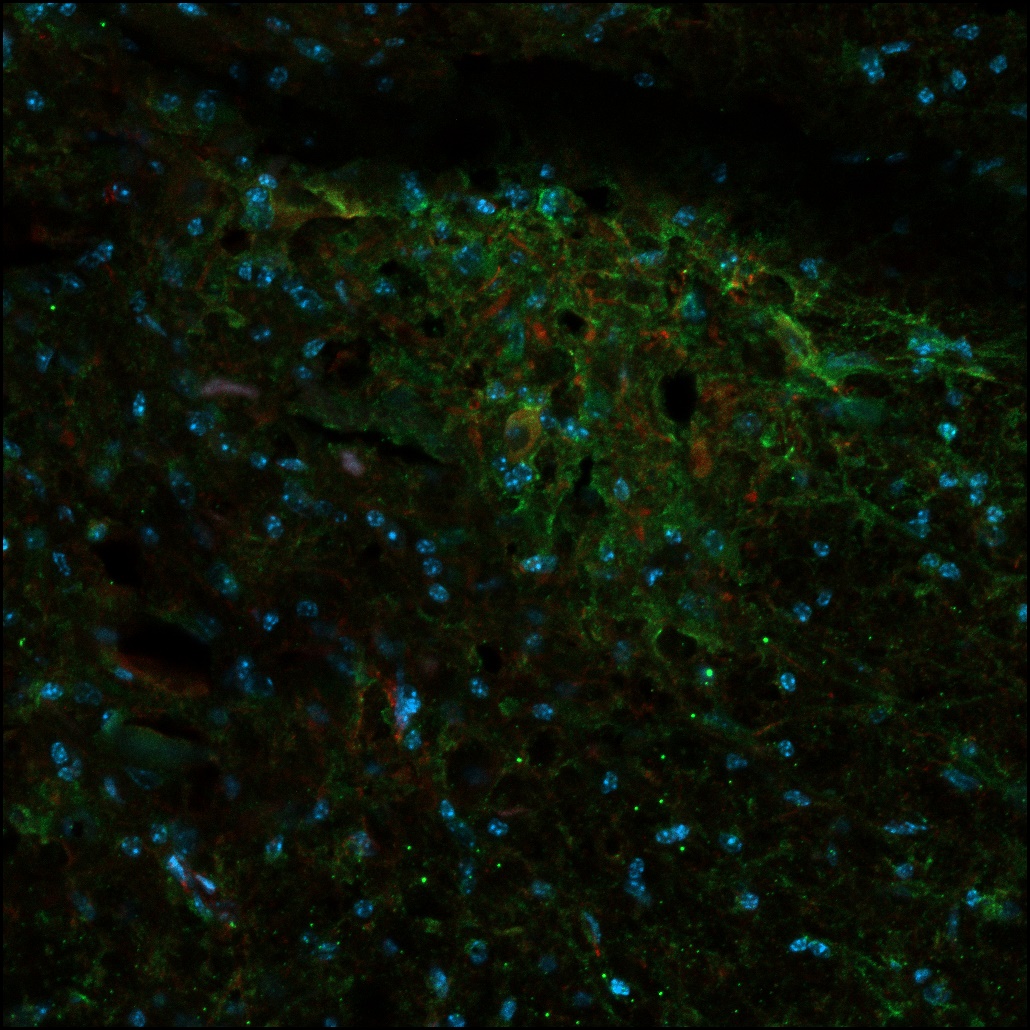

Supplement: Supplementary file 1 [file Data_Sheet_2.ZIP › Fig.1/the expression of RabGGTB in SOD1 mice 3.jpg]

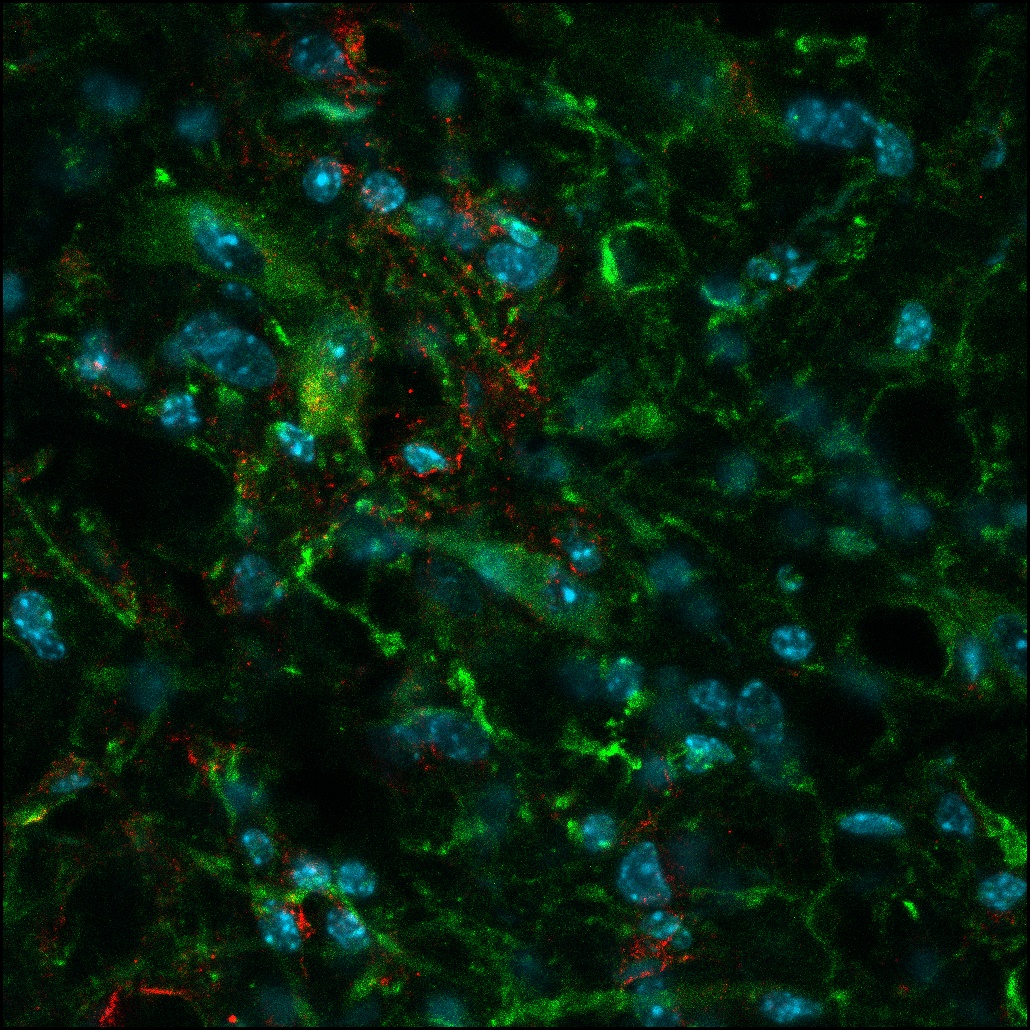

Supplement: Supplementary file 1 [file Data_Sheet_2.ZIP › Fig.1/the expression of RabGGTB in SOD1 mice 4.jpg]

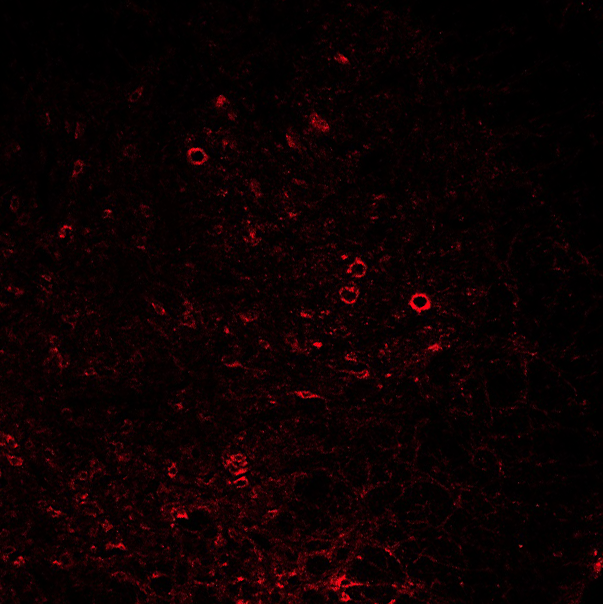

Supplement: Supplementary file 1 [file Data_Sheet_2.ZIP › Fig.1/the expression of RabGGTB in SOD1 mice 5.tif]

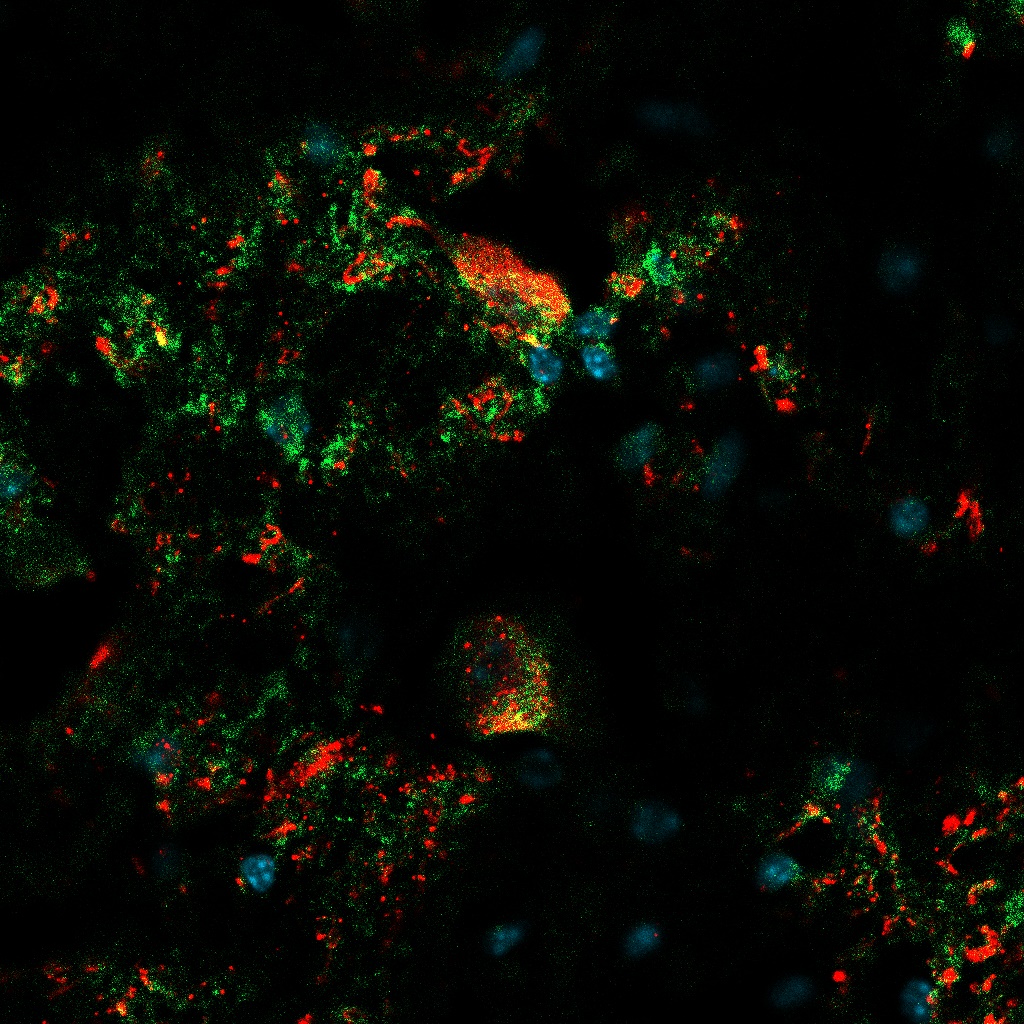

Supplement: Supplementary file 1 [file Data_Sheet_2.ZIP › Fig.1/the expression of RabGGTB in SOD1-KO mice 1.jpg]

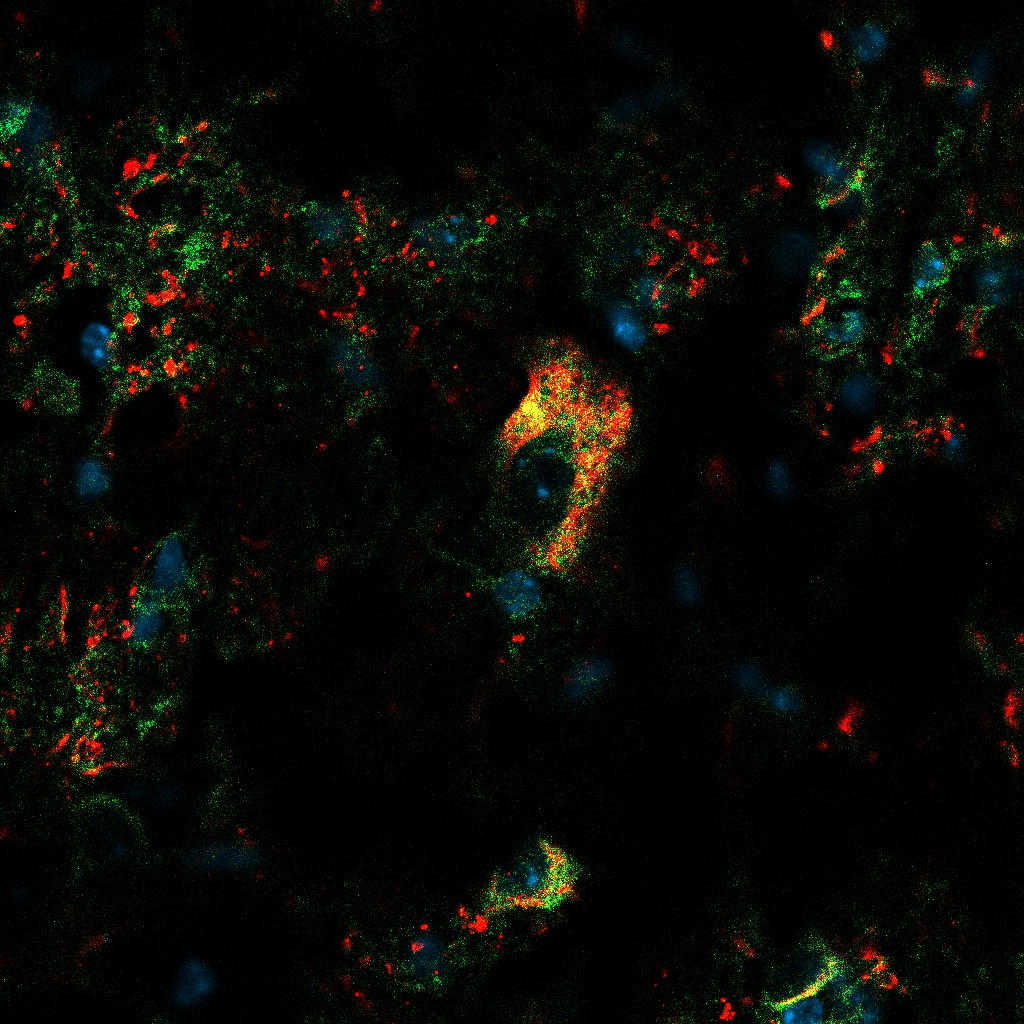

Supplement: Supplementary file 1 [file Data_Sheet_2.ZIP › Fig.1/the expression of RabGGTB in SOD1-KO mice 2.jpg]

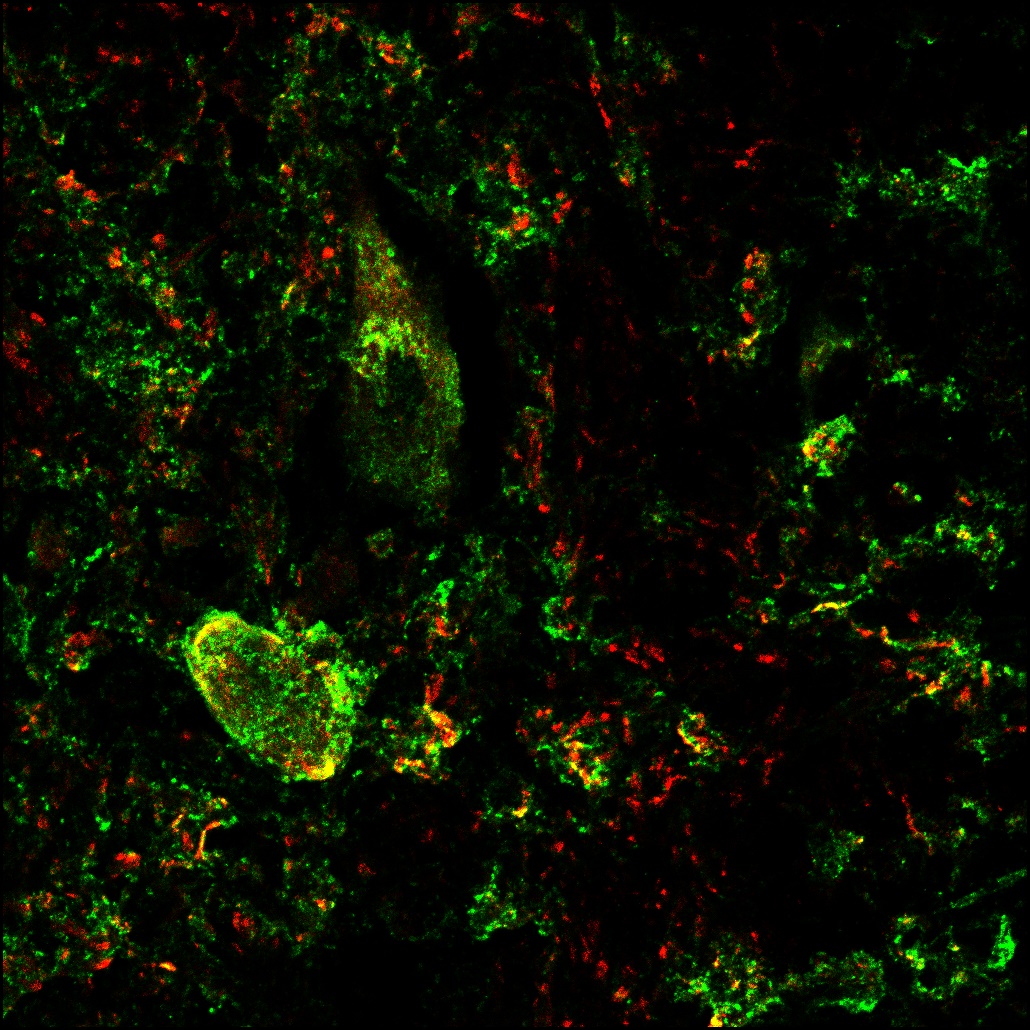

Supplement: Supplementary file 1 [file Data_Sheet_2.ZIP › Fig.1/the expression of RabGGTB in SOD1-KO mice 3.jpg]

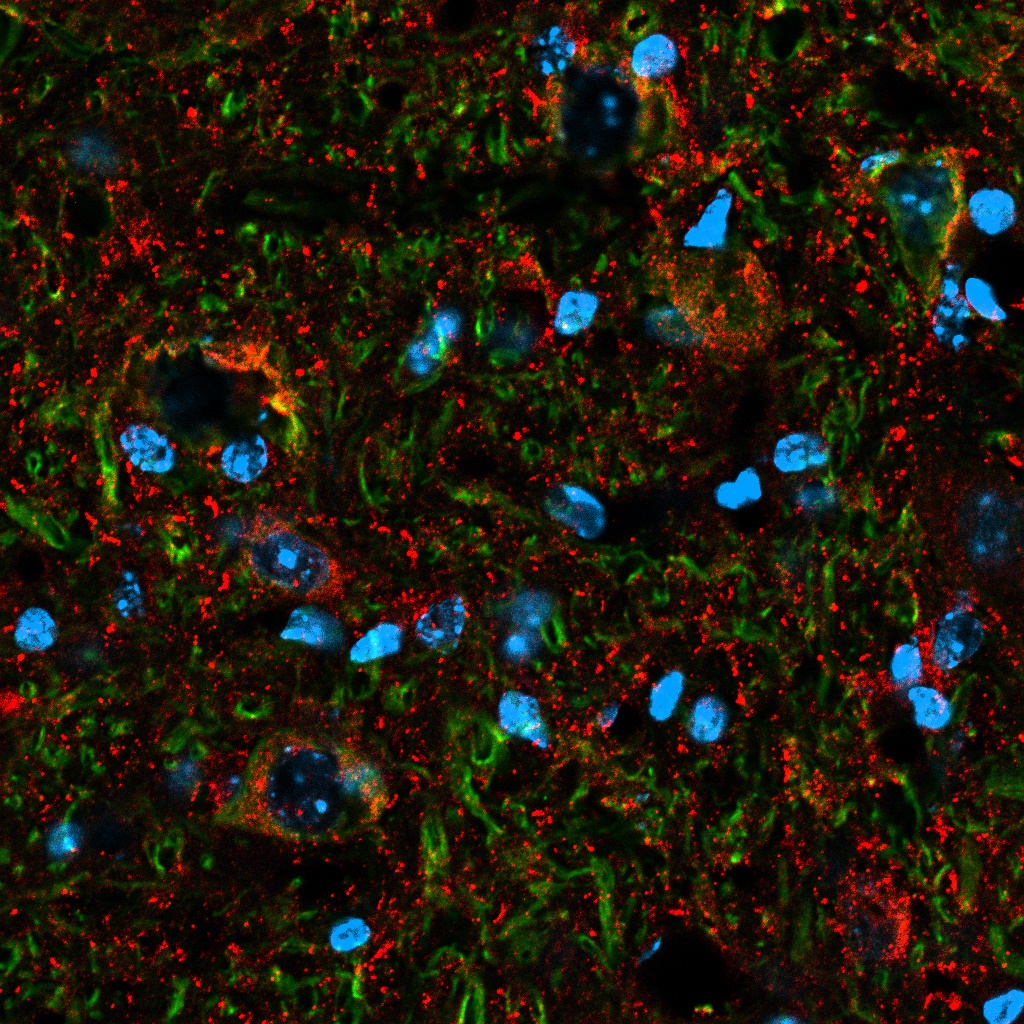

Supplement: Supplementary file 1 [file Data_Sheet_2.ZIP › Fig.1/the expression of RabGGTB in SOD1-KO mice 4.jpg]

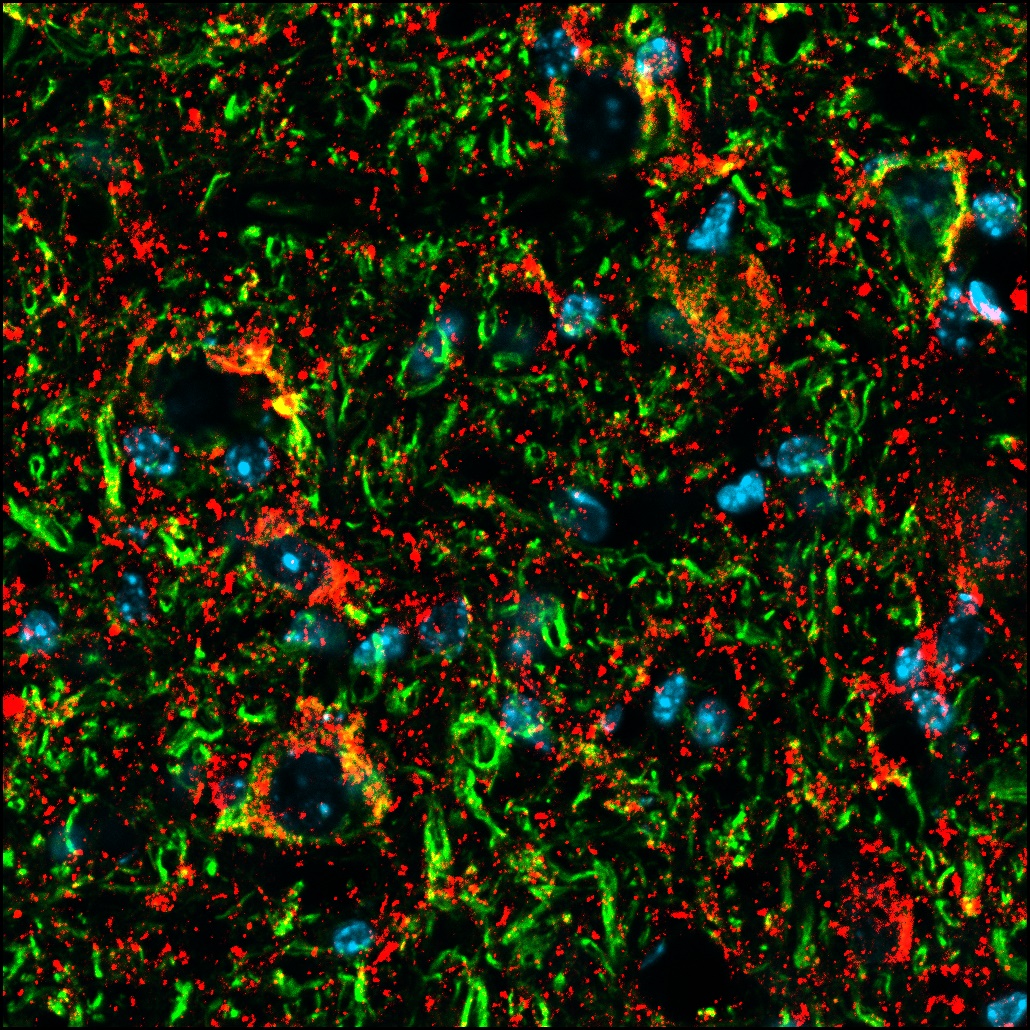

Supplement: Supplementary file 1 [file Data_Sheet_2.ZIP › Fig.1/the expression of RabGGTB in WT mice 1.jpg]

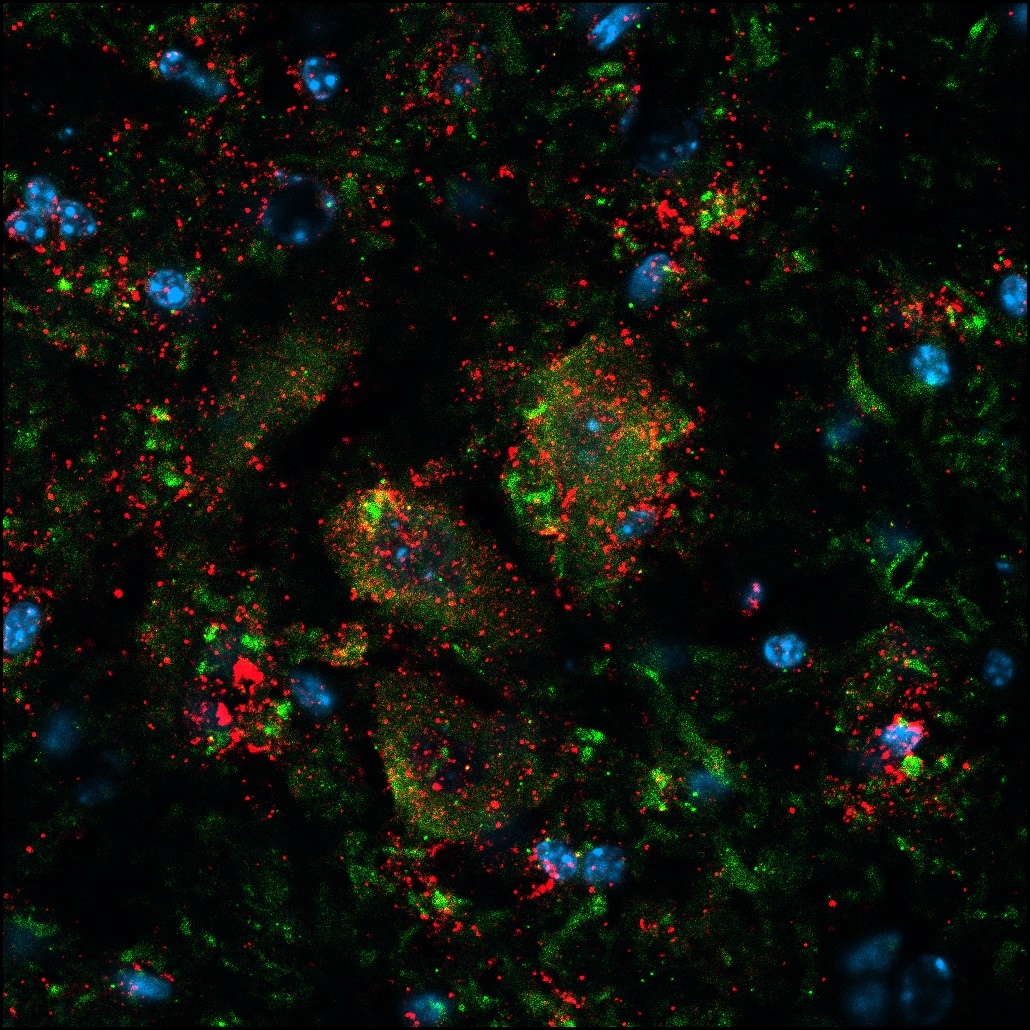

Supplement: Supplementary file 1 [file Data_Sheet_2.ZIP › Fig.1/the expression of RabGGTB in WT mice 2.jpg]

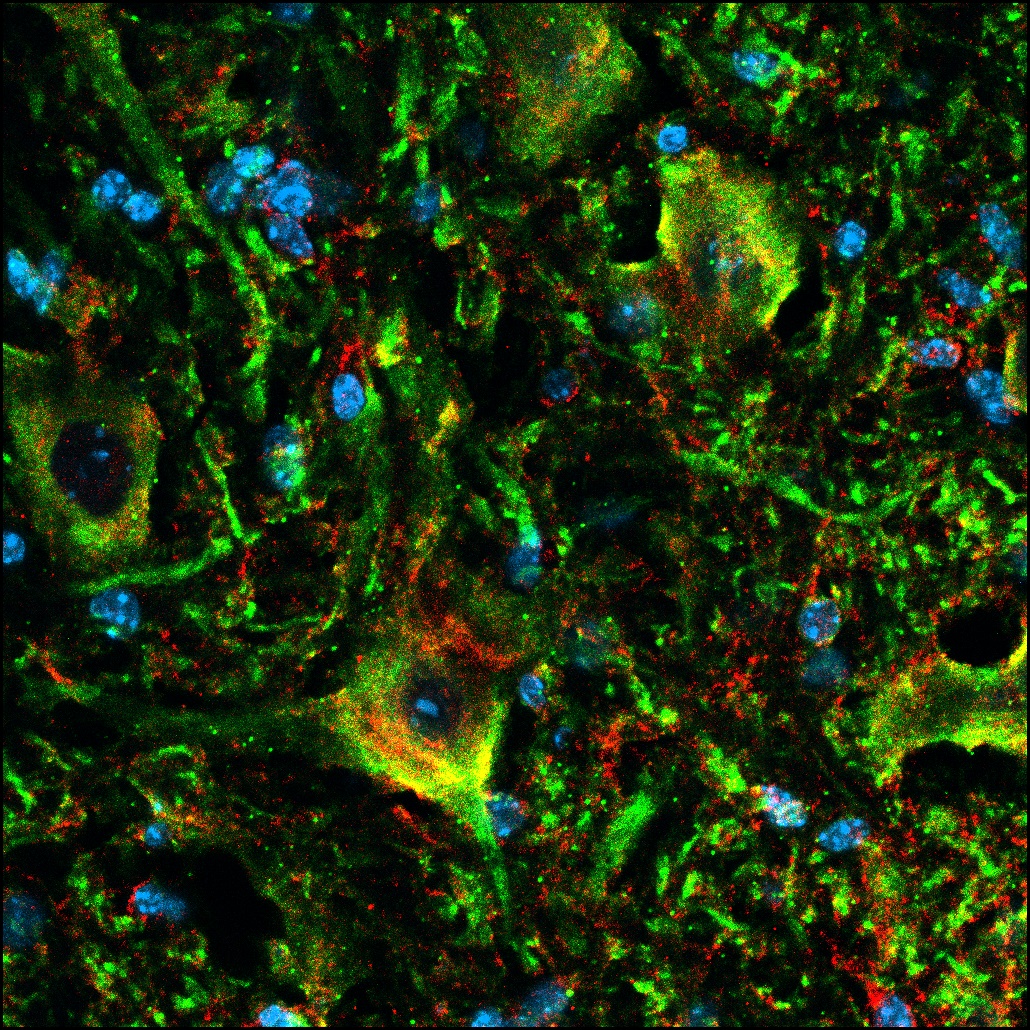

Supplement: Supplementary file 1 [file Data_Sheet_2.ZIP › Fig.1/the expression of RabGGTB in WT mice 3.jpg]

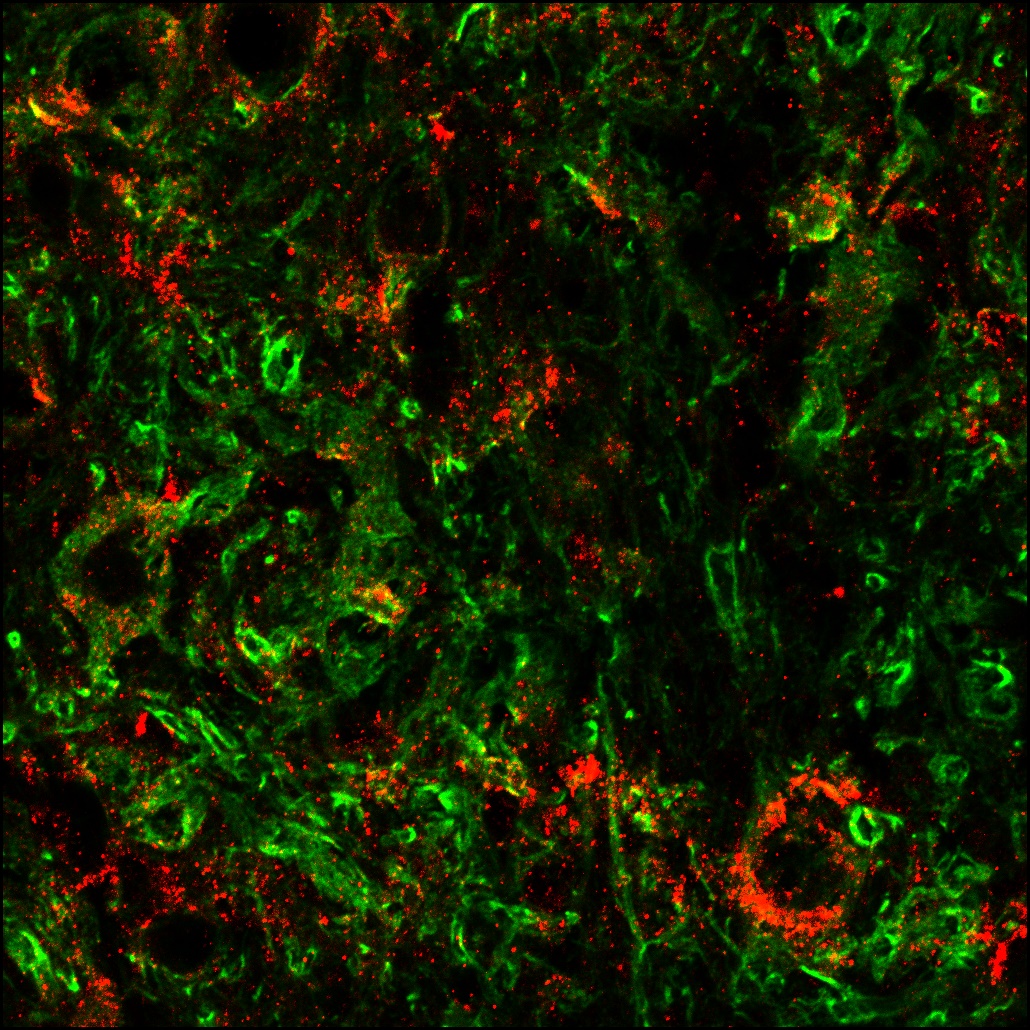

Supplement: Supplementary file 1 [file Data_Sheet_2.ZIP › Fig.1/the expression of RabGGTB in WT mice 4.jpg]

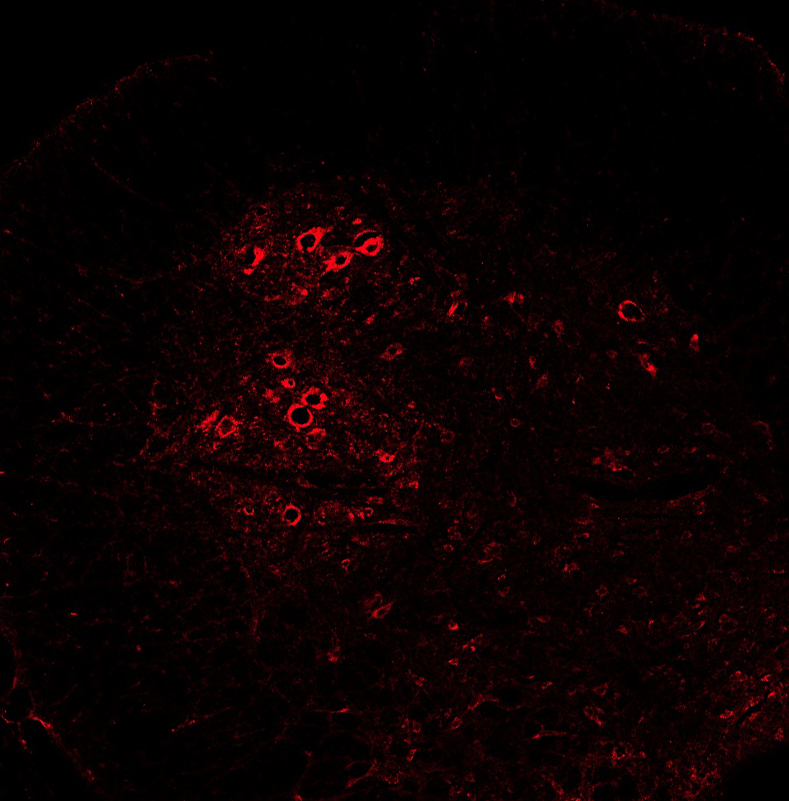

Supplement: Supplementary file 1 [file Data_Sheet_2.ZIP › Fig.1/the expression of RabGGTB in WT mice 5.tif]

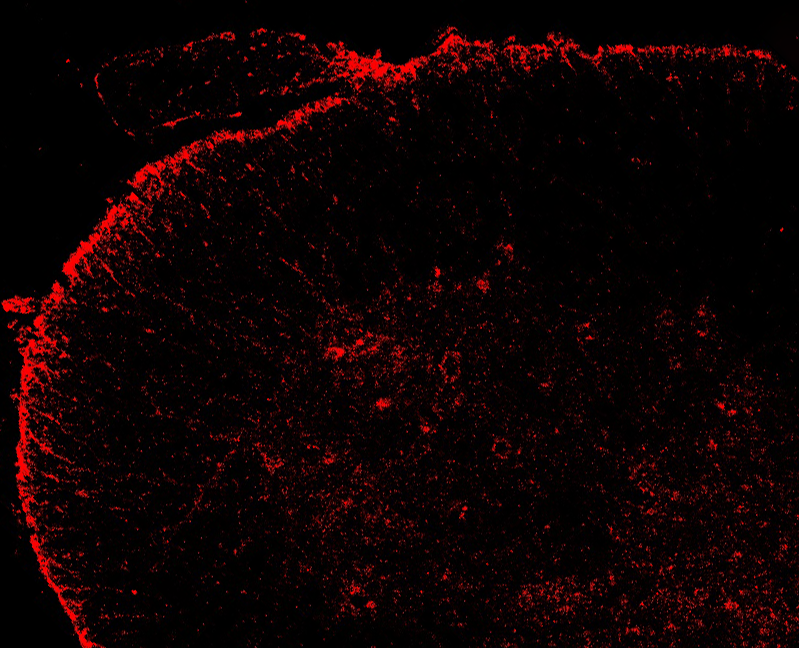

Supplement: Supplementary file 2 [file Data_Sheet_3.ZIP › Fig.2/218-ggtb647-neun594-gfp-5x-pin-z_c1+2+3+4.tif]

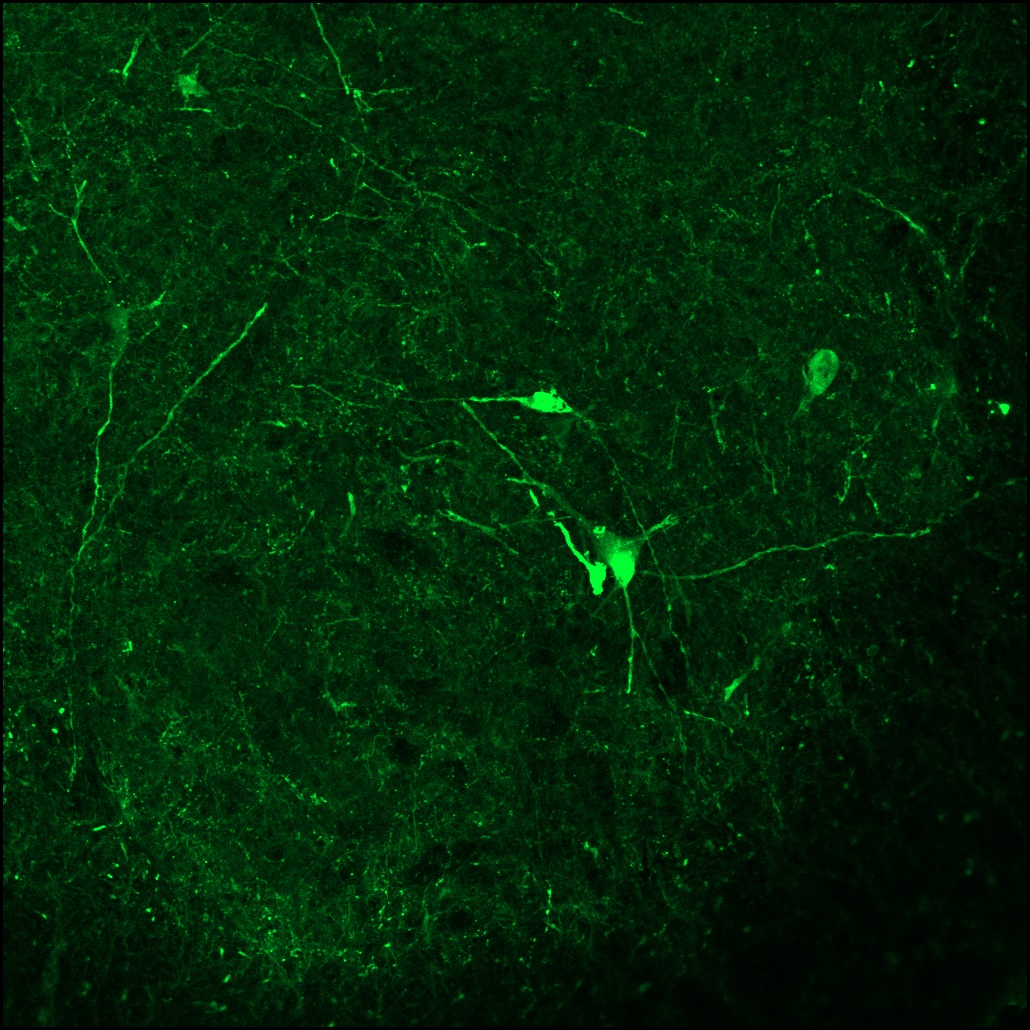

Supplement: Supplementary file 2 [file Data_Sheet_3.ZIP › Fig.2/The GFP expression in C.jpg]

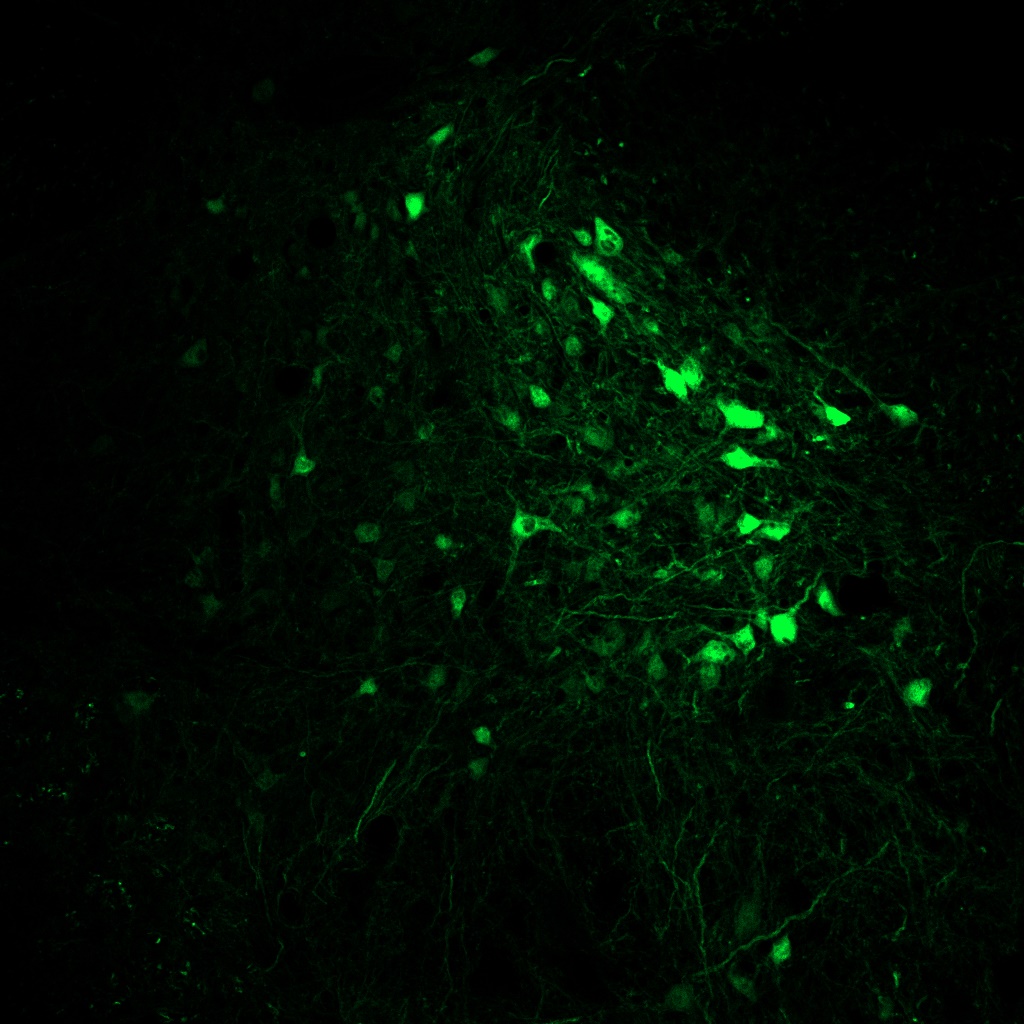

Supplement: Supplementary file 2 [file Data_Sheet_3.ZIP › Fig.2/The GFP expression in Thorax 1.jpg]

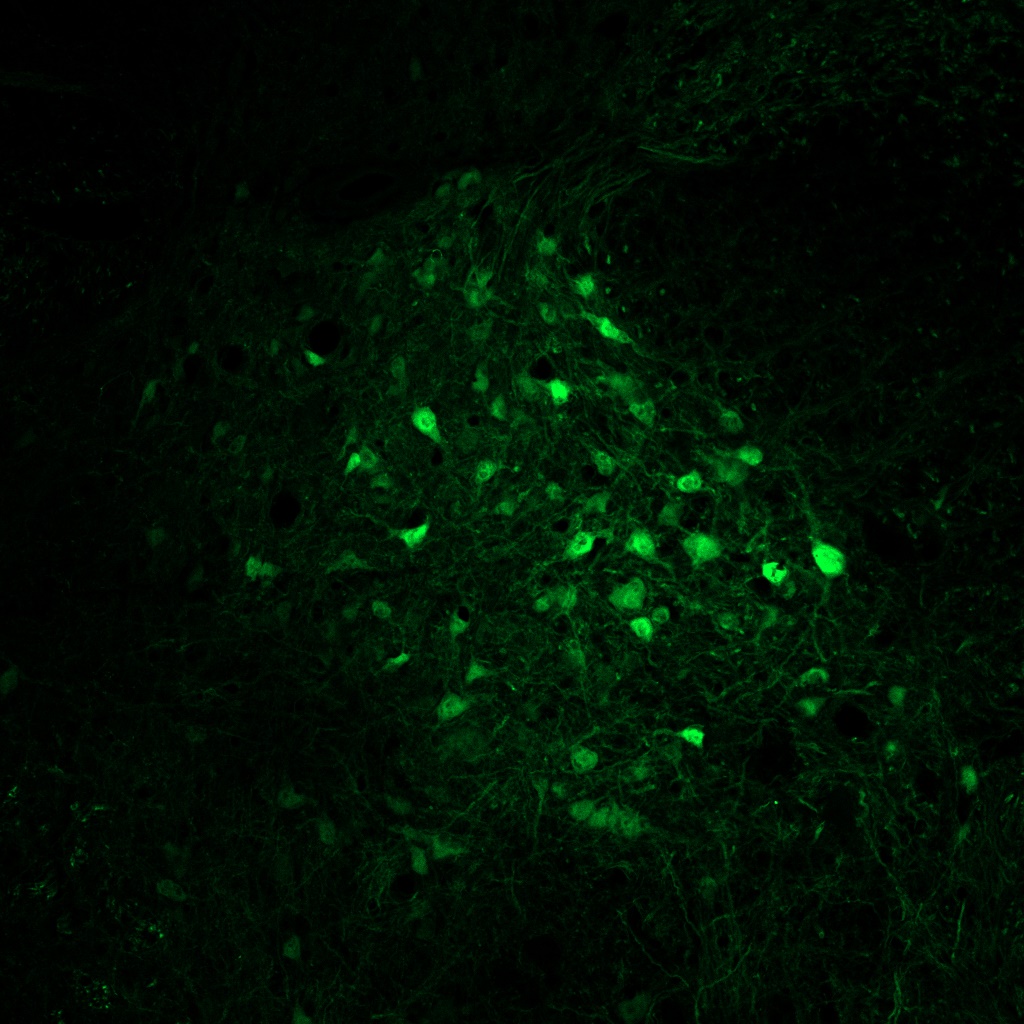

Supplement: Supplementary file 2 [file Data_Sheet_3.ZIP › Fig.2/The GFP expression in Thorax 2.jpg]

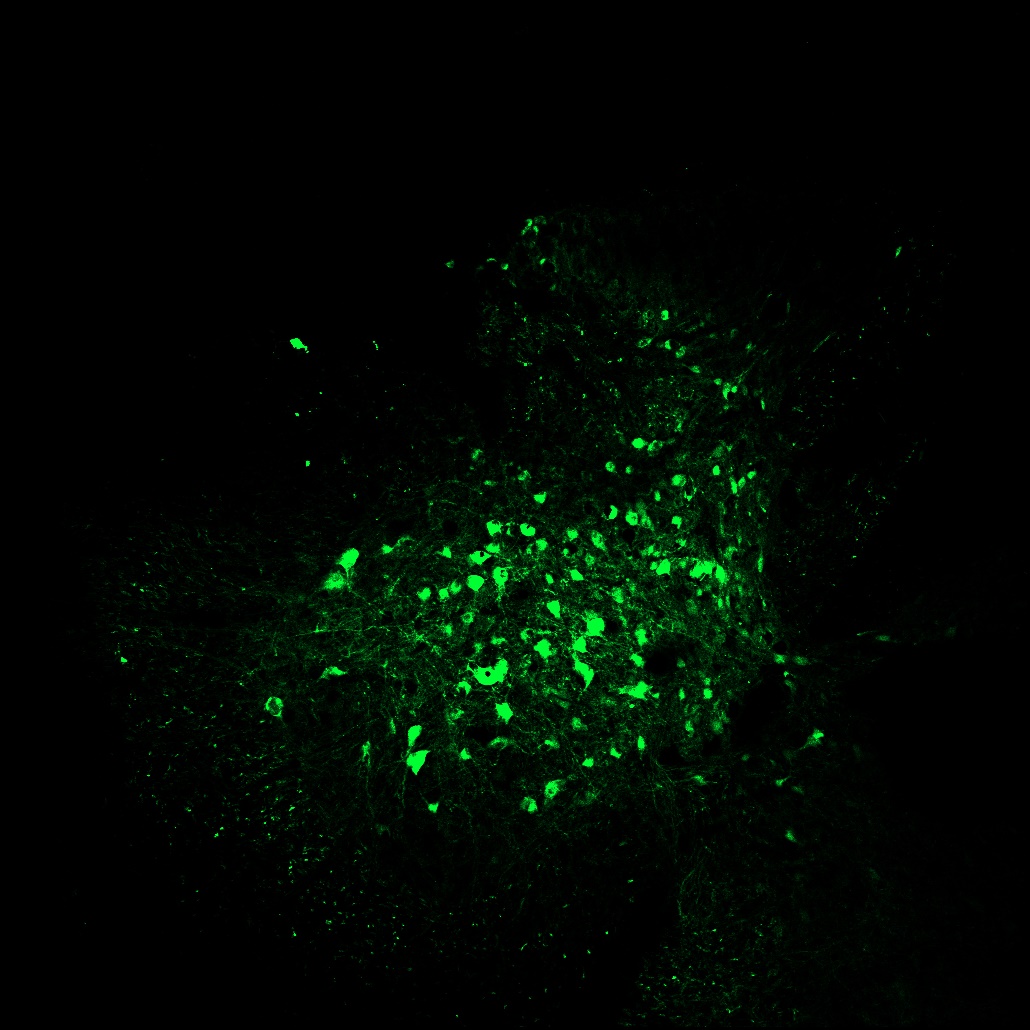

Supplement: Supplementary file 2 [file Data_Sheet_3.ZIP › Fig.2/The GFP expression in Thorax 3.jpg]

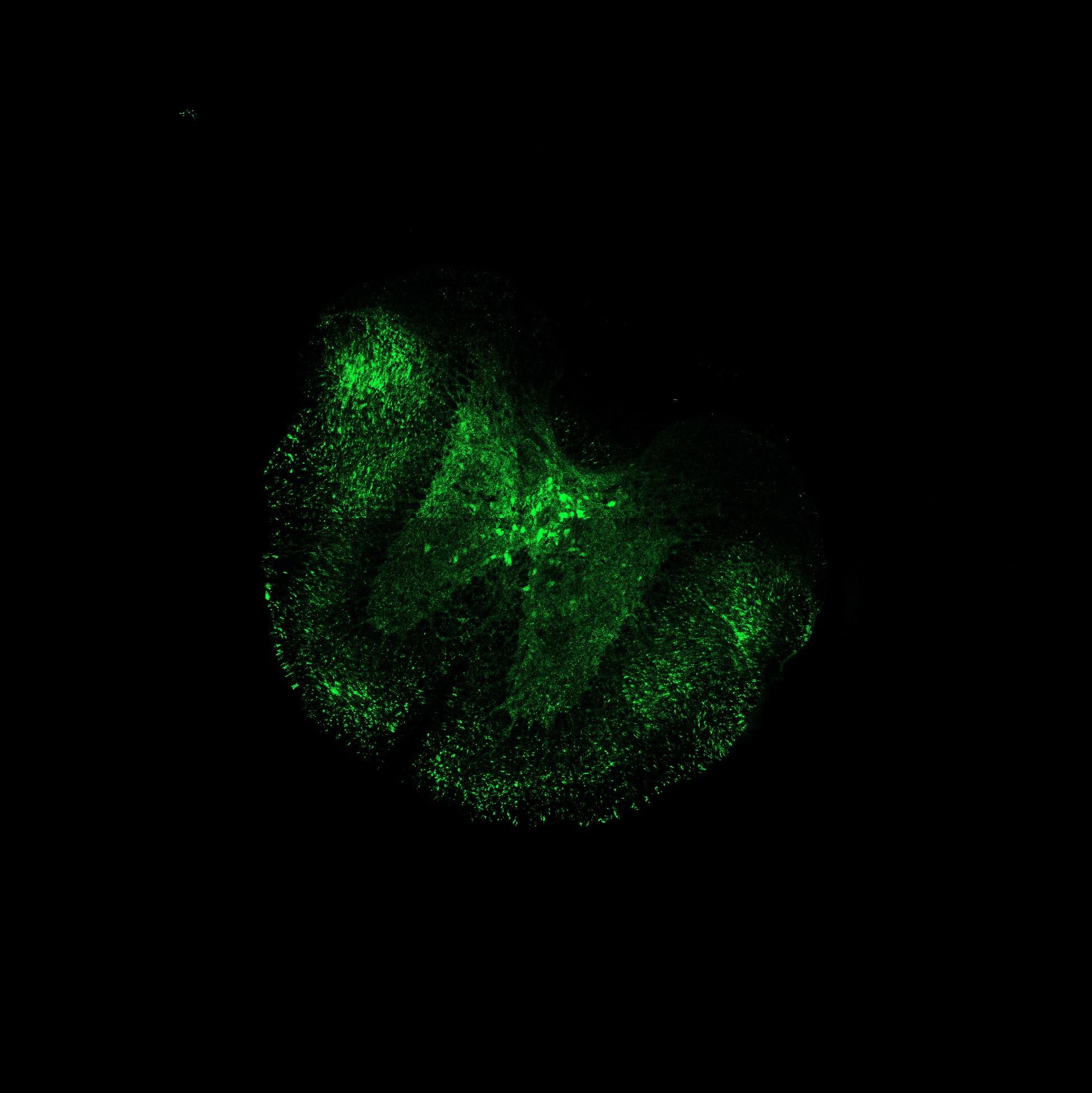

Supplement: Supplementary file 2 [file Data_Sheet_3.ZIP › Fig.2/The GFP expression in Thorax 4.jpg]

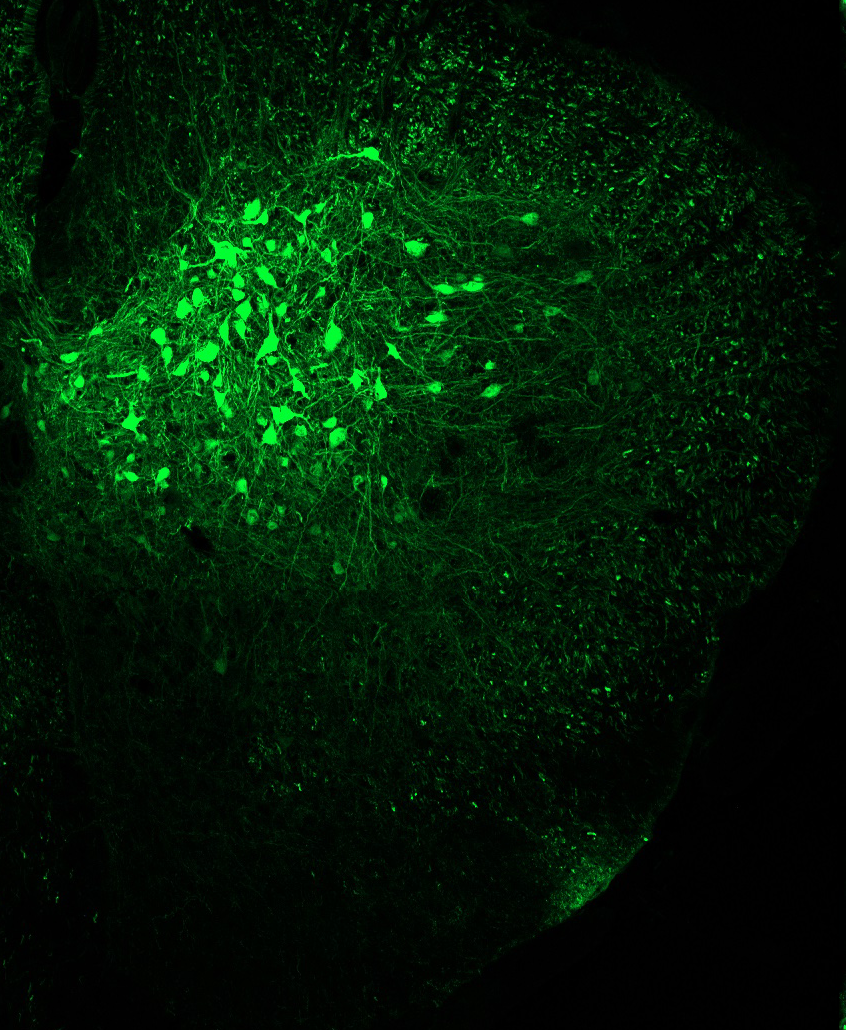

Supplement: Supplementary file 2 [file Data_Sheet_3.ZIP › Fig.2/The GFP expression in lumbar 2.tif]

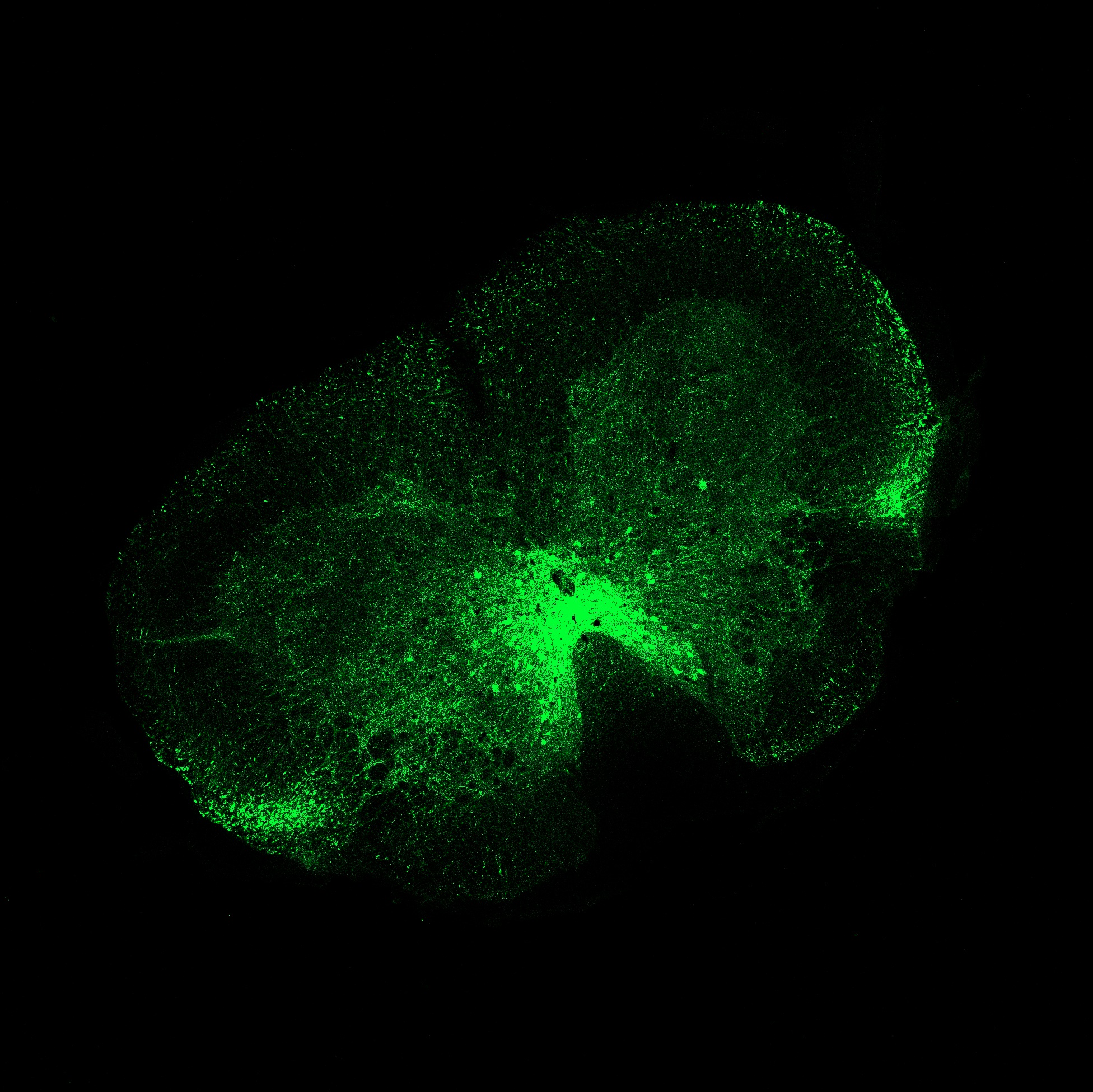

Supplement: Supplementary file 2 [file Data_Sheet_3.ZIP › Fig.2/The GFP expression in lumbar 4.jpg]

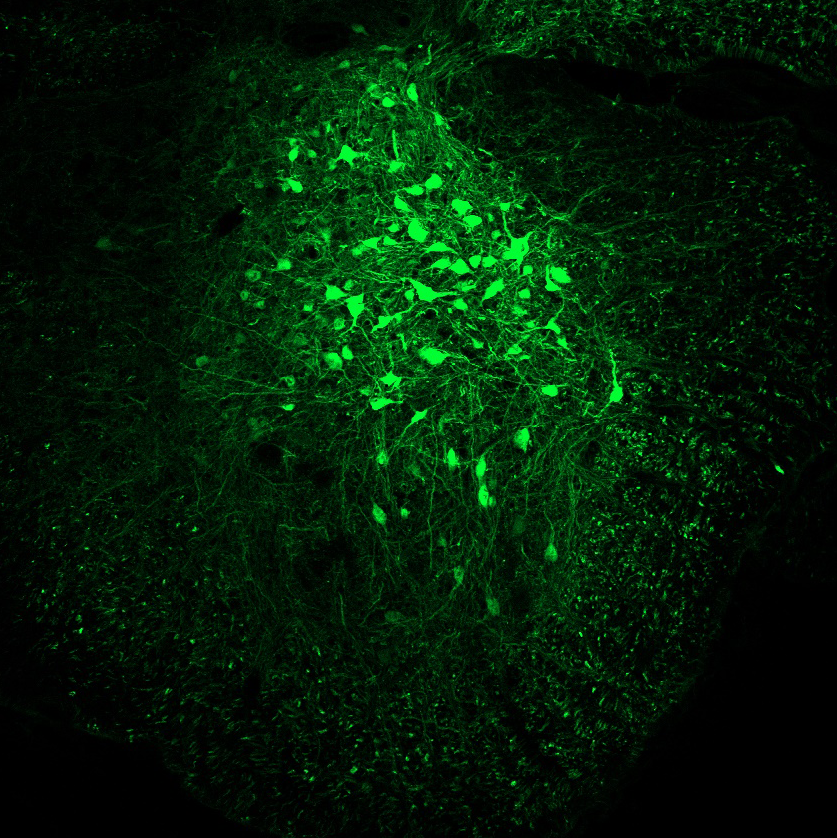

Supplement: Supplementary file 2 [file Data_Sheet_3.ZIP › Fig.2/The GFP expression in lumbar.tif]

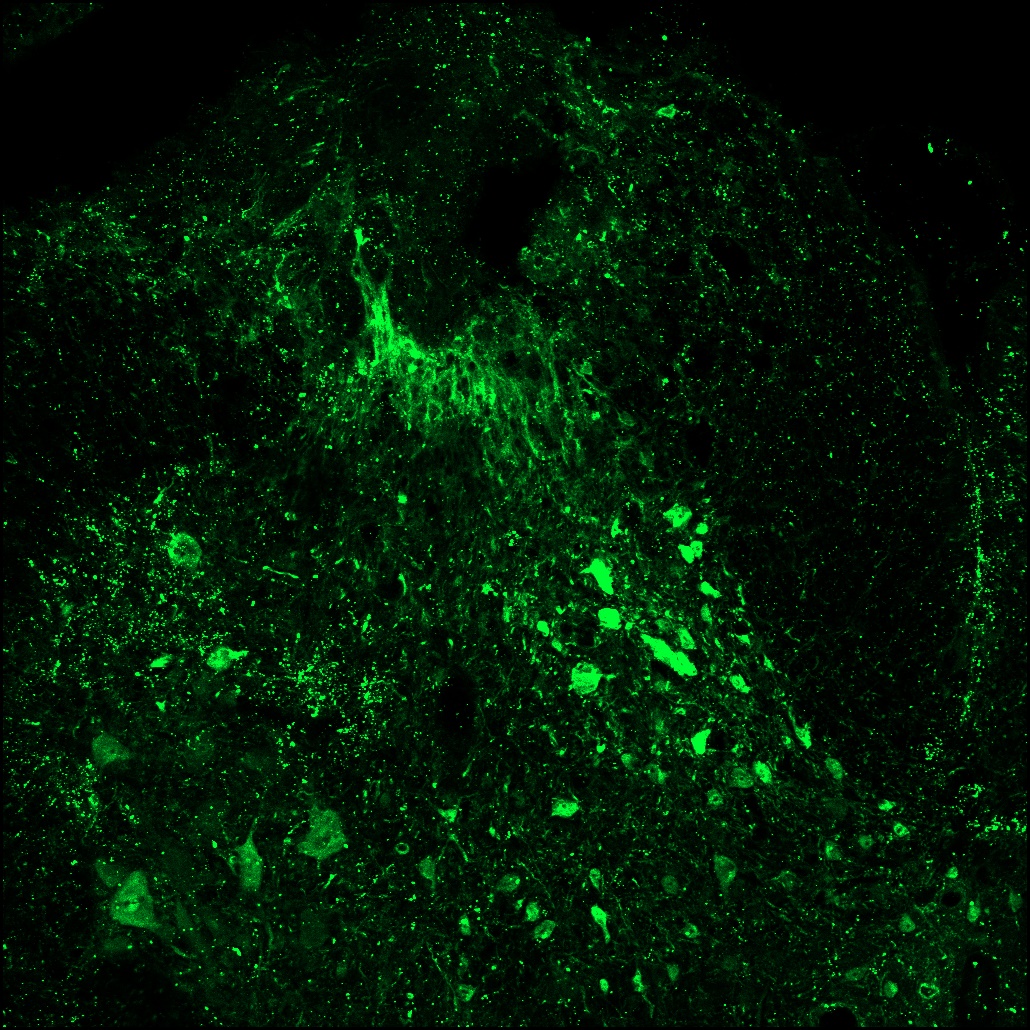

Supplement: Supplementary file 2 [file Data_Sheet_3.ZIP › Fig.2/The GFP expression in lumbar2.jpg]

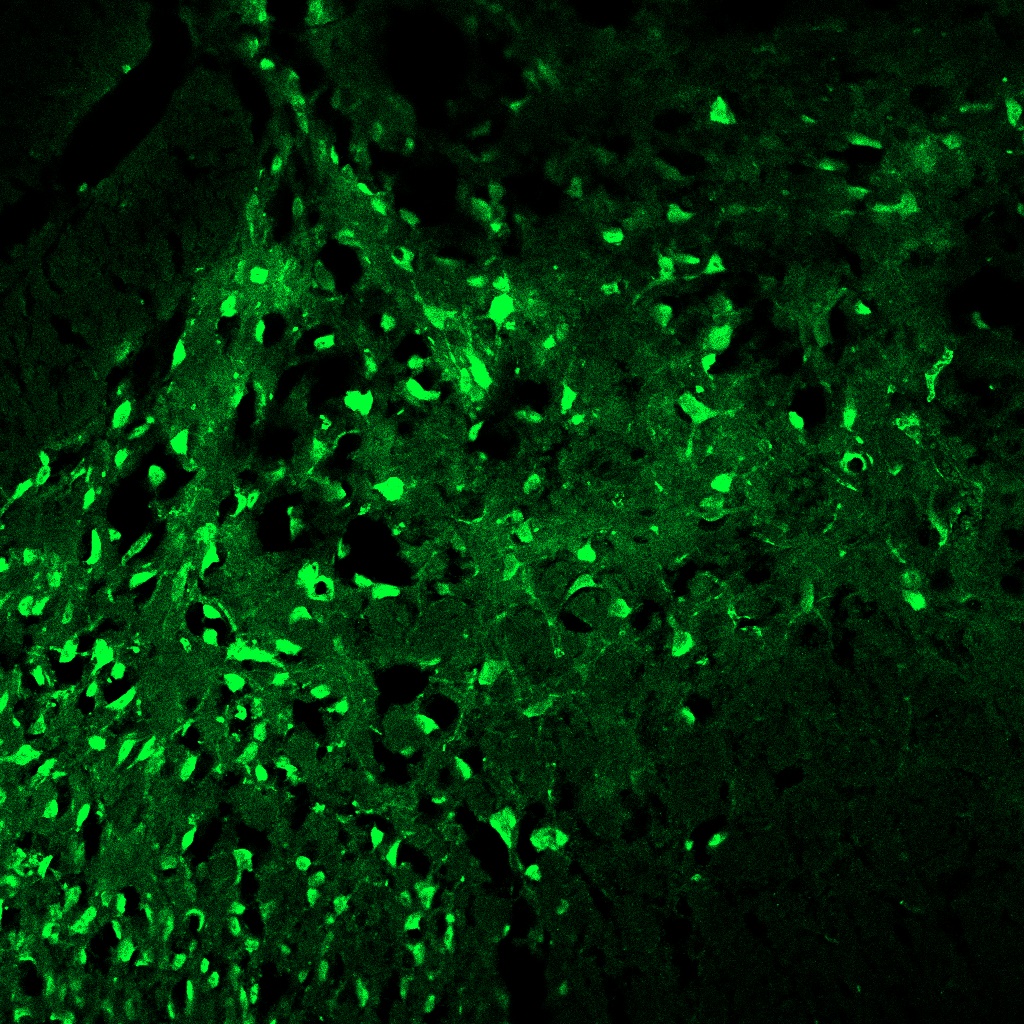

Supplement: Supplementary file 2 [file Data_Sheet_3.ZIP › Fig.2/The GFP expression in lumbar3.jpg]

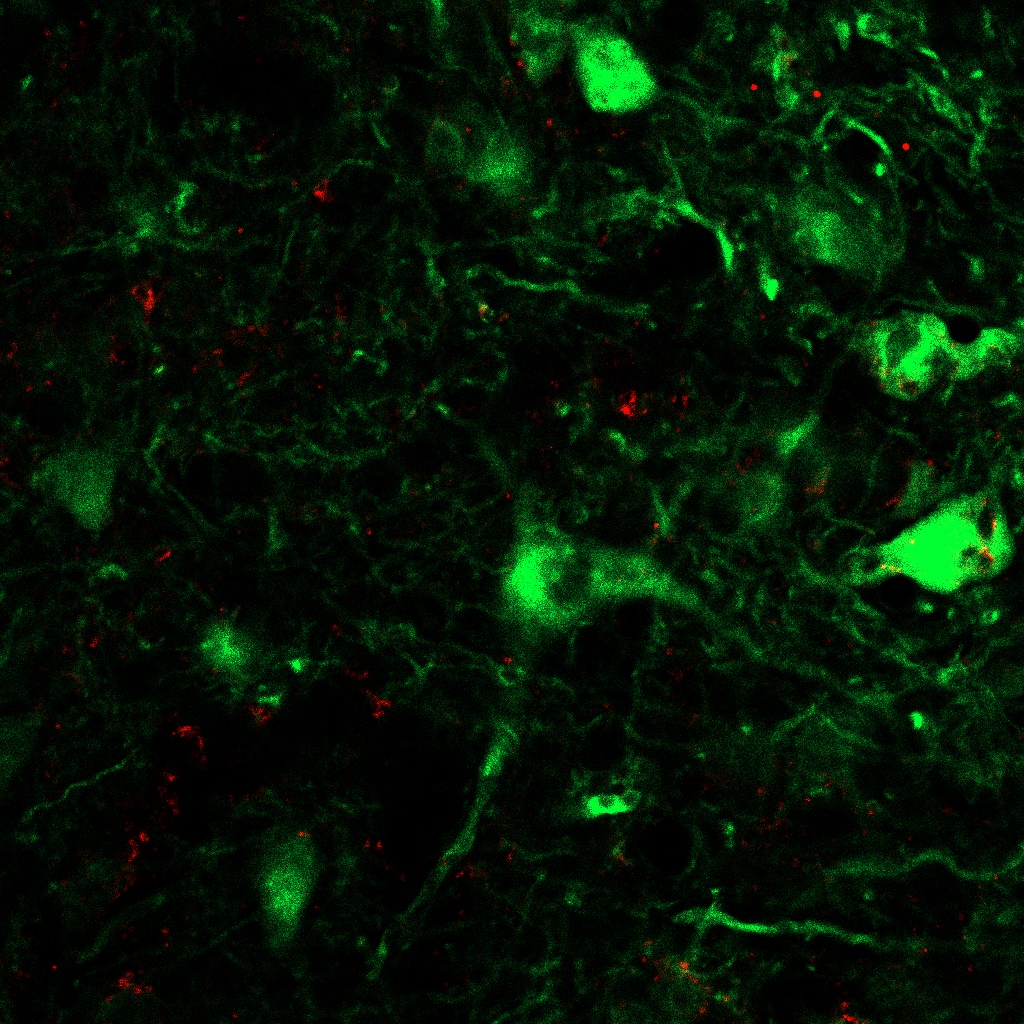

Supplement: Supplementary file 2 [file Data_Sheet_3.ZIP › Fig.2/The expression of RabGGTB in SDO1mice injected with AAV9-GFP+1.jpg]

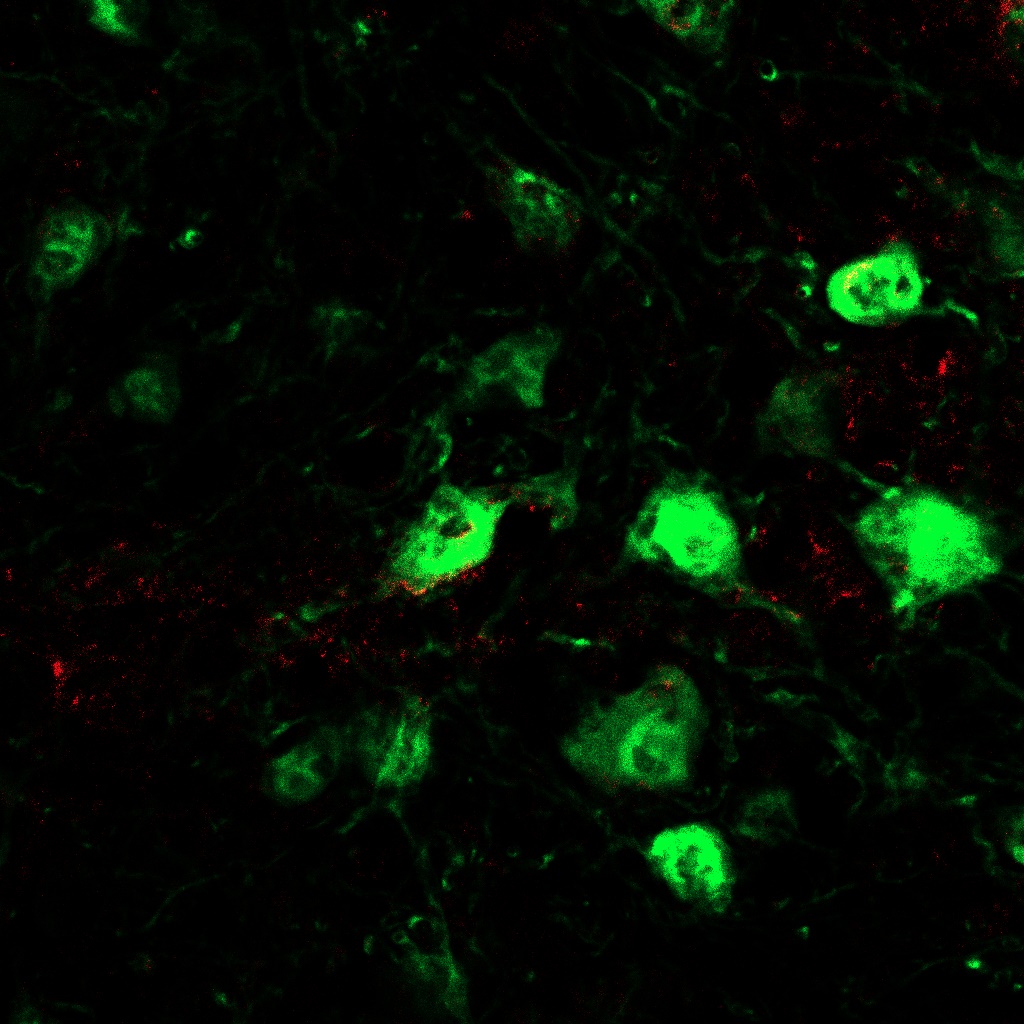

Supplement: Supplementary file 2 [file Data_Sheet_3.ZIP › Fig.2/The expression of RabGGTB in SDO1mice injected with AAV9-GFP+2.jpg]

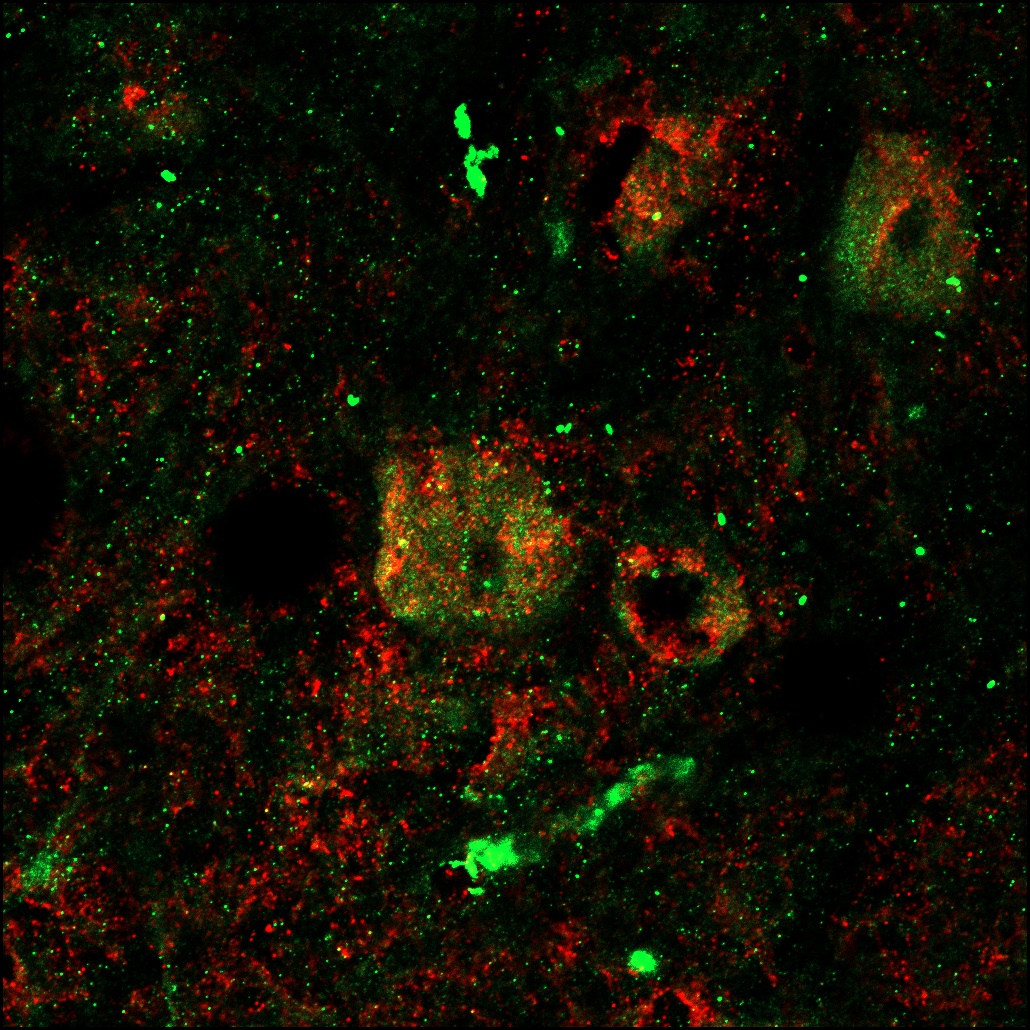

Supplement: Supplementary file 2 [file Data_Sheet_3.ZIP › Fig.2/The expression of RabGGTB in SDO1mice injected with AAV9-RabGGTB 1.jpg]

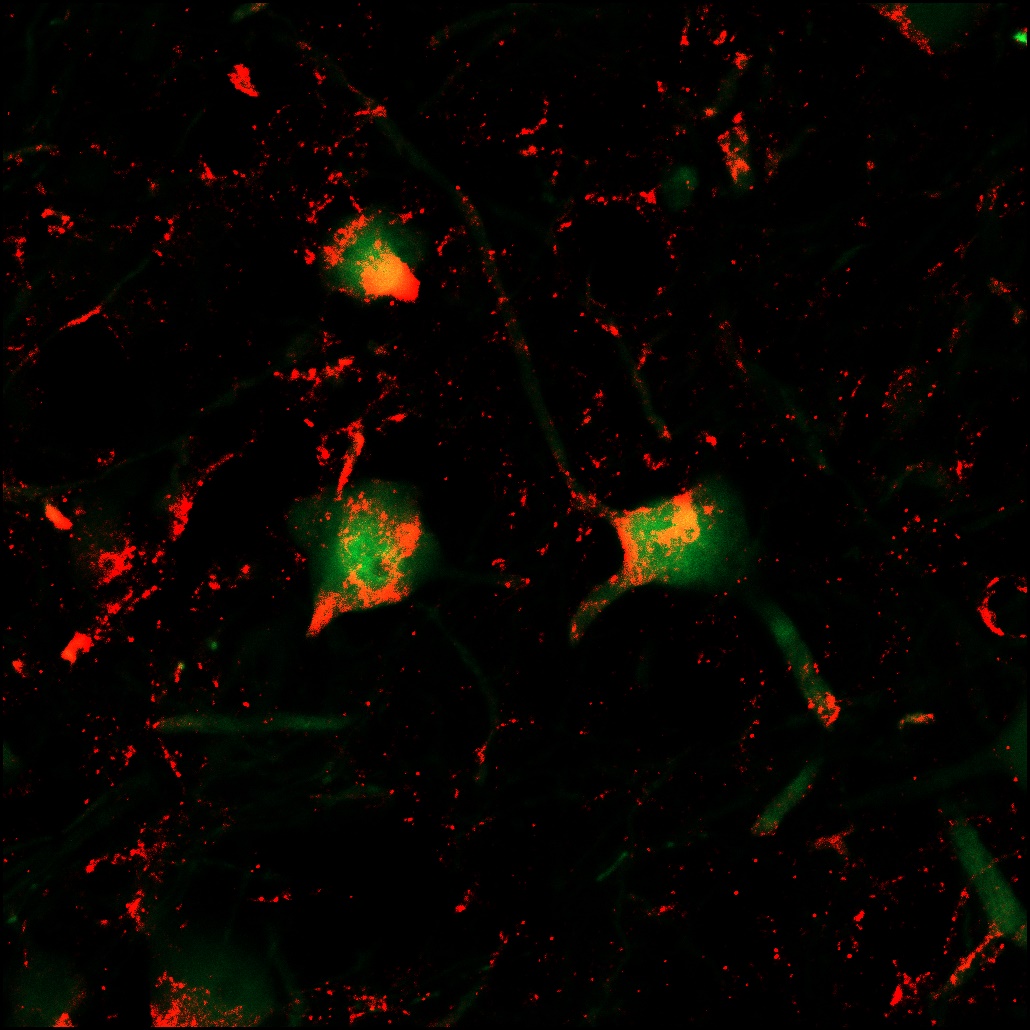

Supplement: Supplementary file 2 [file Data_Sheet_3.ZIP › Fig.2/The expression of RabGGTB in SDO1mice injected with AAV9-RabGGTB 2.jpg]

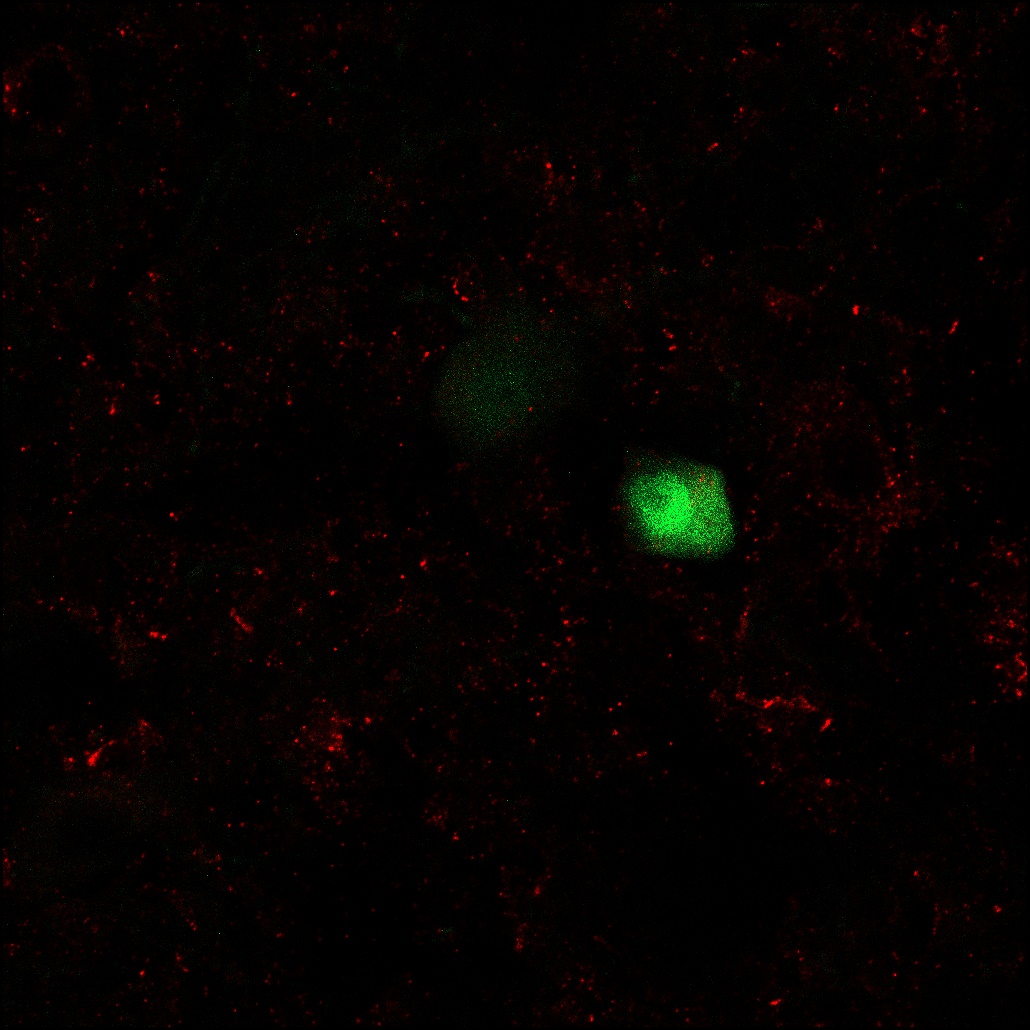

Supplement: Supplementary file 2 [file Data_Sheet_3.ZIP › Fig.2/The expression of RabGGTB in SDO1mice injected with AAV9-RabGGTB.jpg]

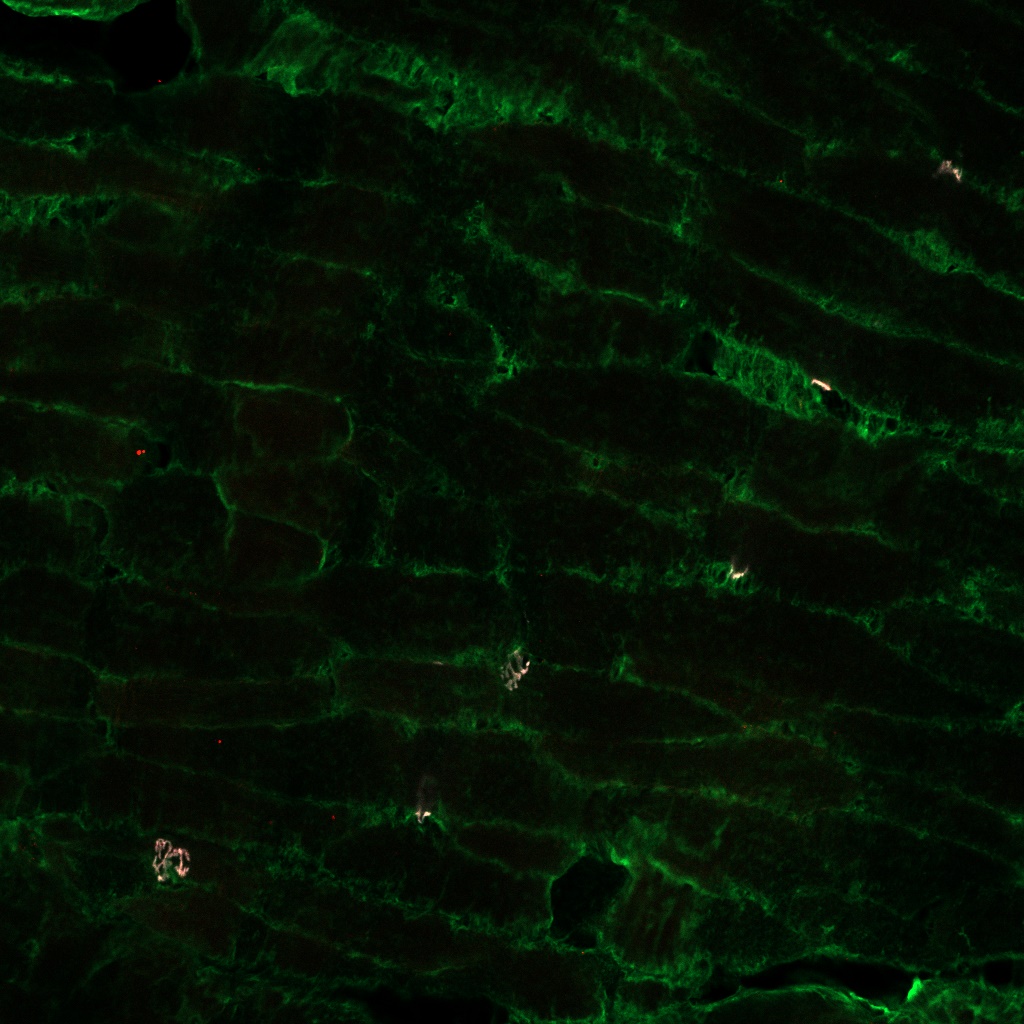

Supplement: Supplementary file 4 [file Data_Sheet_5.ZIP › Fig.5/NMJ-SOD1G93A mice injected with AAV9-GFP+1.jpg]

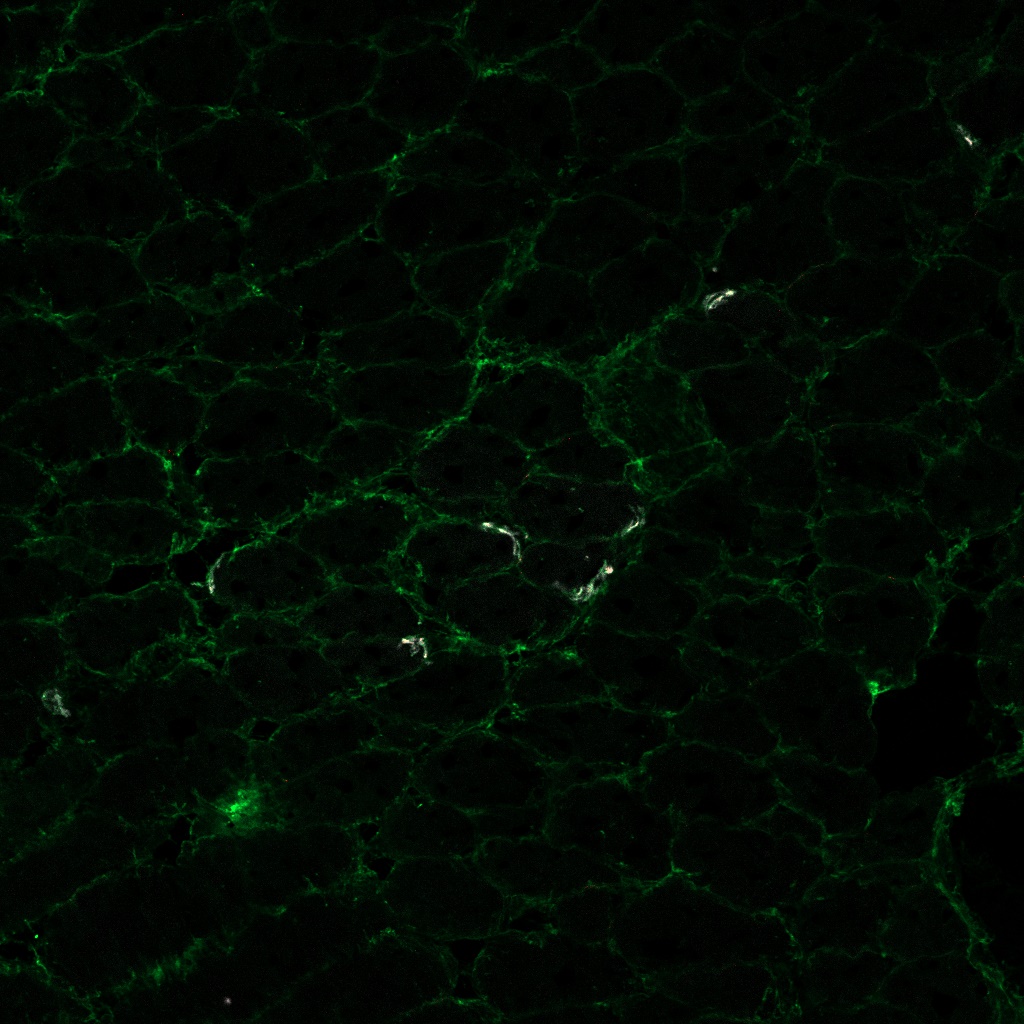

Supplement: Supplementary file 4 [file Data_Sheet_5.ZIP › Fig.5/NMJ-SOD1G93A mice injected with AAV9-GFP+3.jpg]

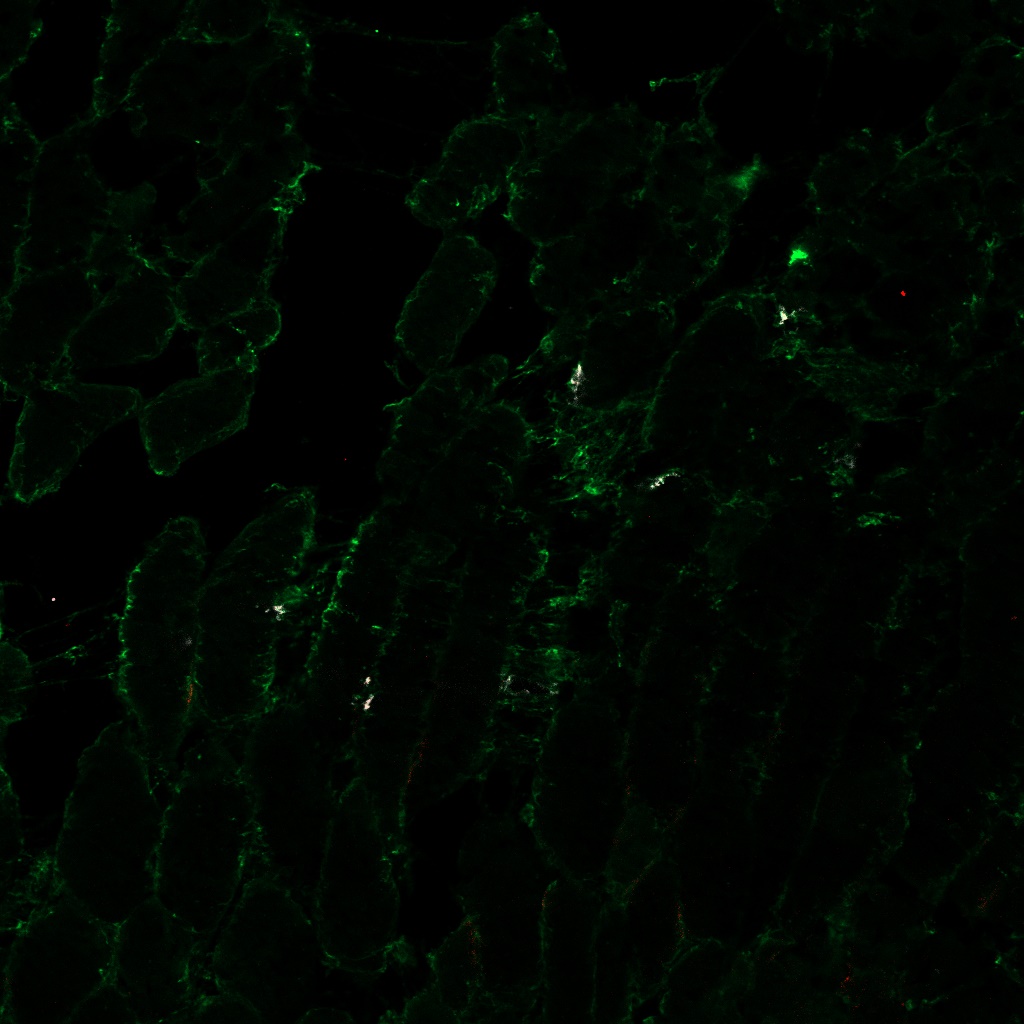

Supplement: Supplementary file 4 [file Data_Sheet_5.ZIP › Fig.5/NMJ-SOD1G93A mice injected with AAV9-GFP+4.jpg]

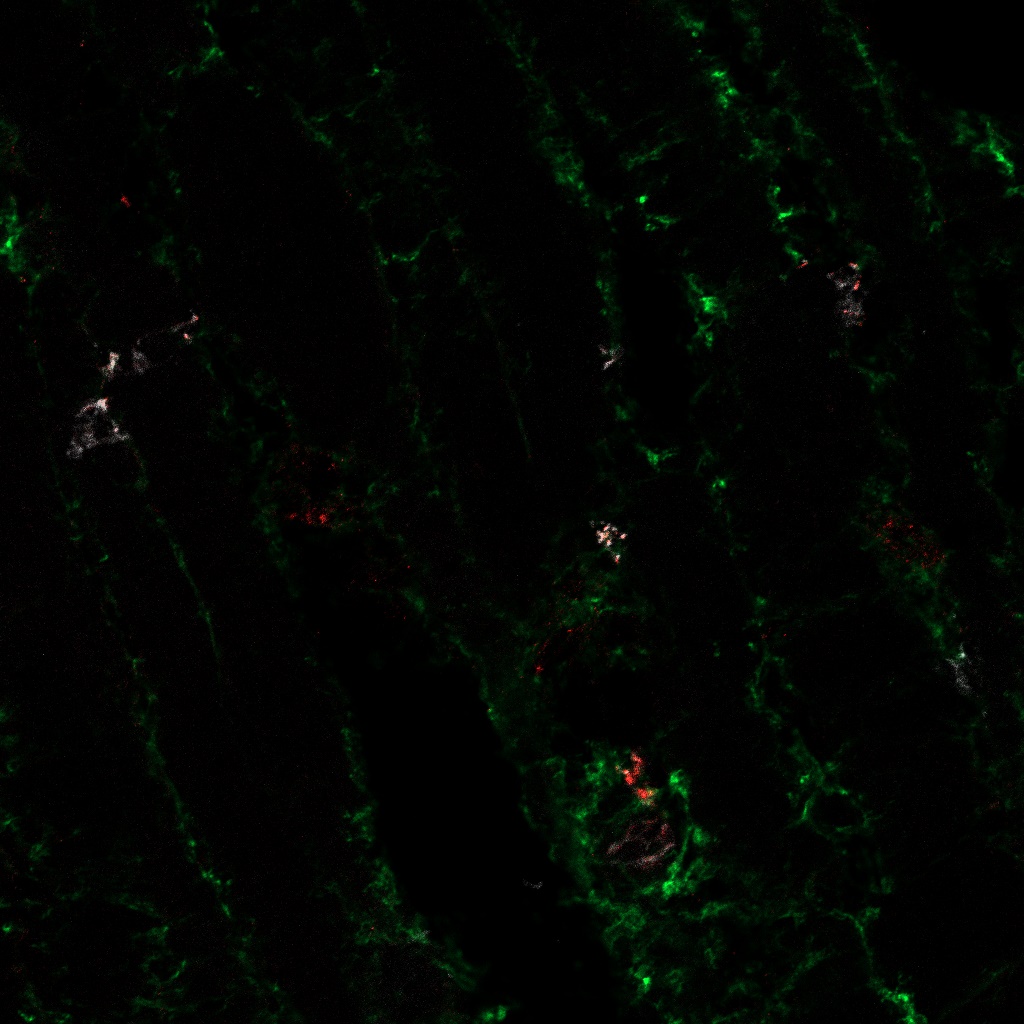

Supplement: Supplementary file 4 [file Data_Sheet_5.ZIP › Fig.5/NMJ-SOD1G93A mice injected with AAV9-GFP+6.jpg]

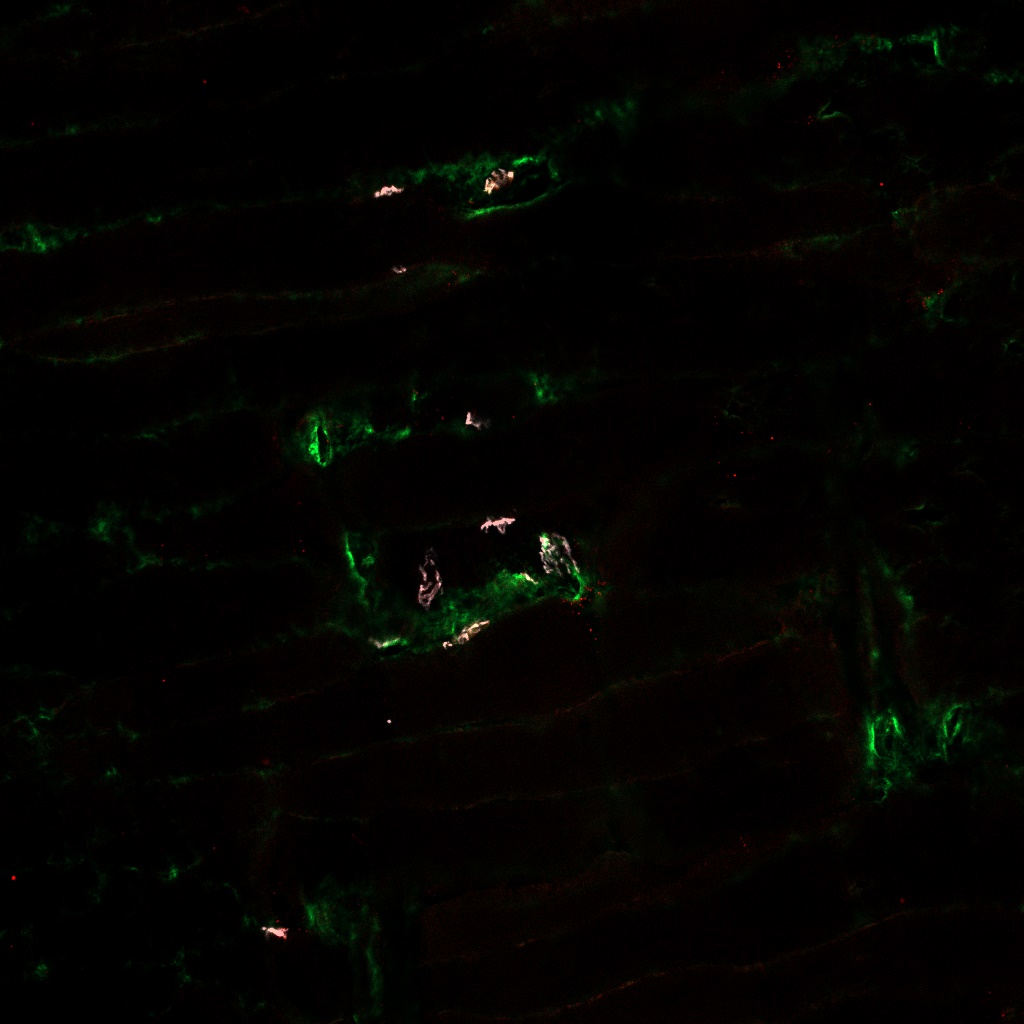

Supplement: Supplementary file 4 [file Data_Sheet_5.ZIP › Fig.5/NMJ-SOD1G93A mice injected with AAV9-RabGGTB-GFP+1.jpg]

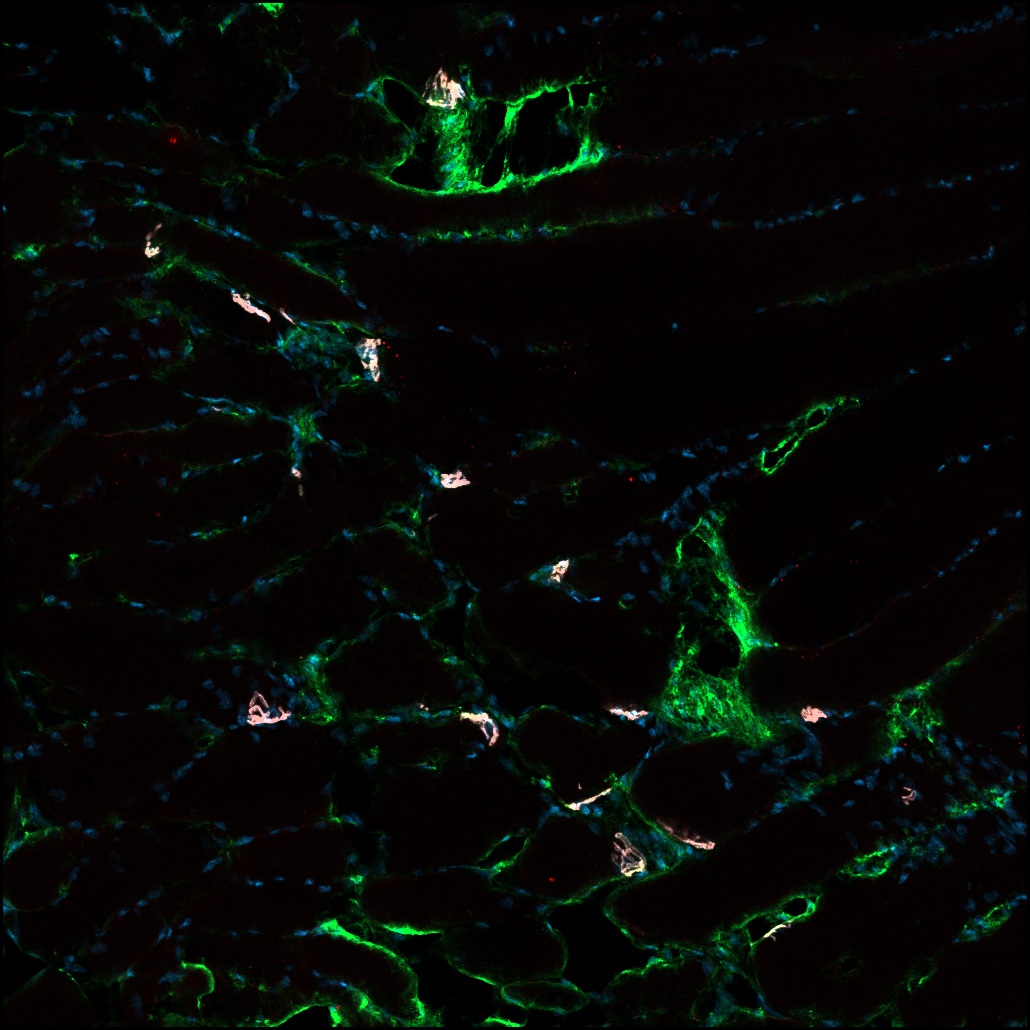

Supplement: Supplementary file 4 [file Data_Sheet_5.ZIP › Fig.5/NMJ-SOD1G93A mice injected with AAV9-RabGGTB-GFP+2.jpg]

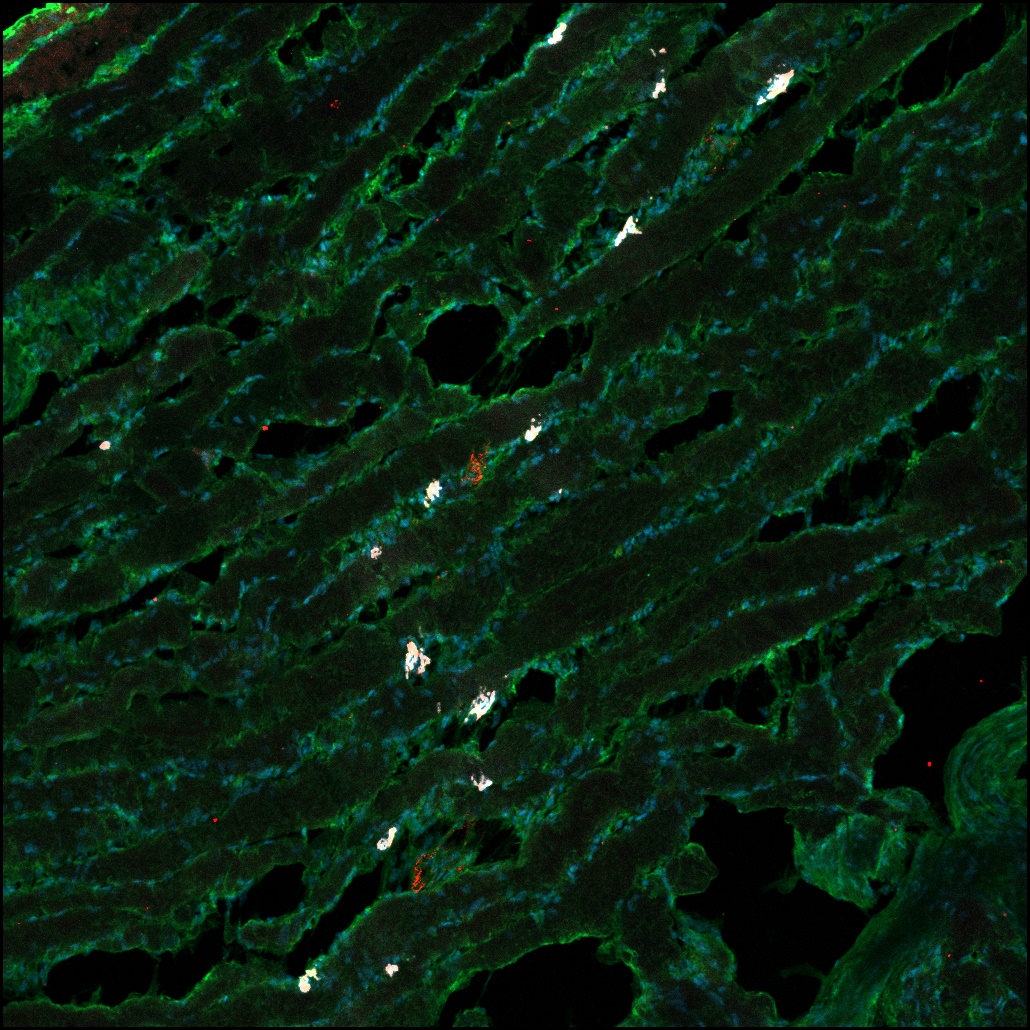

Supplement: Supplementary file 4 [file Data_Sheet_5.ZIP › Fig.5/NMJ-SOD1G93A mice injected with AAV9-RabGGTB-GFP+3.jpg]

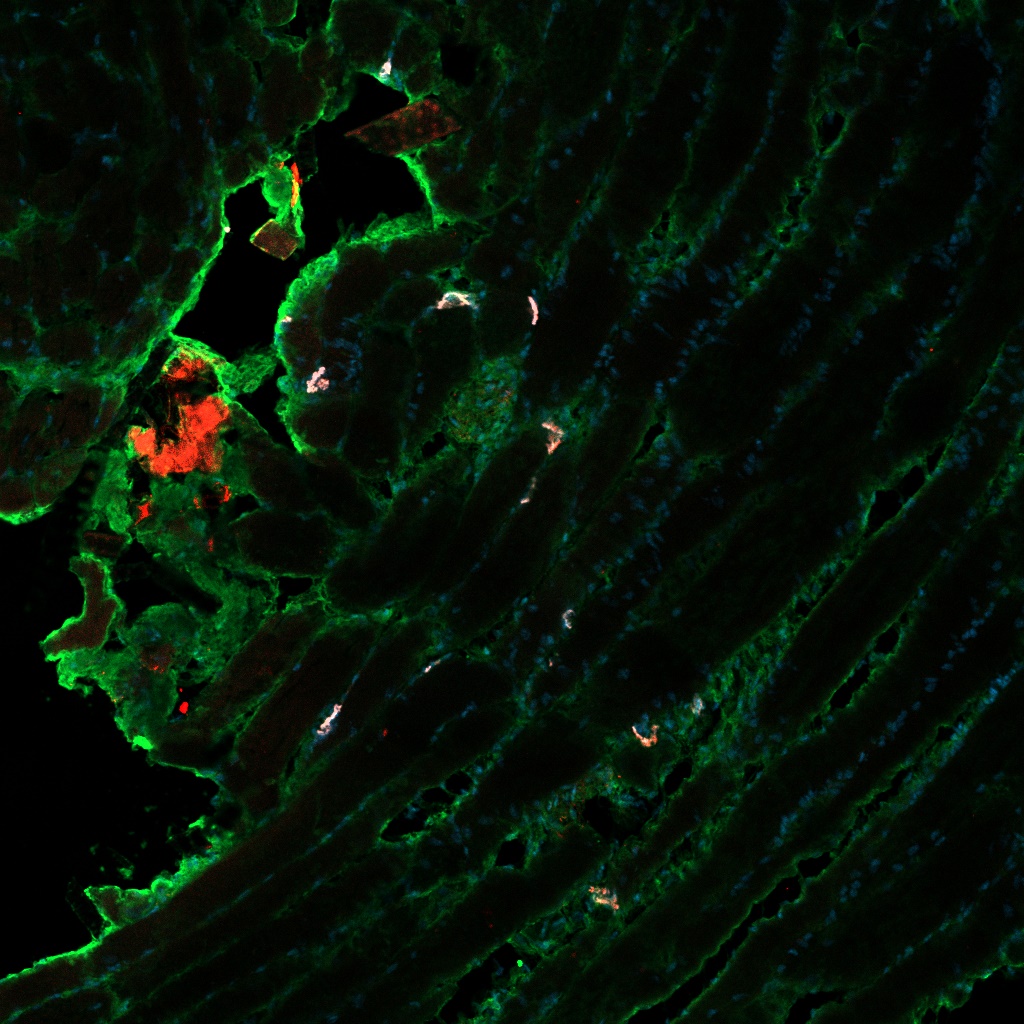

Supplement: Supplementary file 4 [file Data_Sheet_5.ZIP › Fig.5/NMJ-SOD1G93A mice injected with AAV9-RabGGTB-GFP+4.jpg]

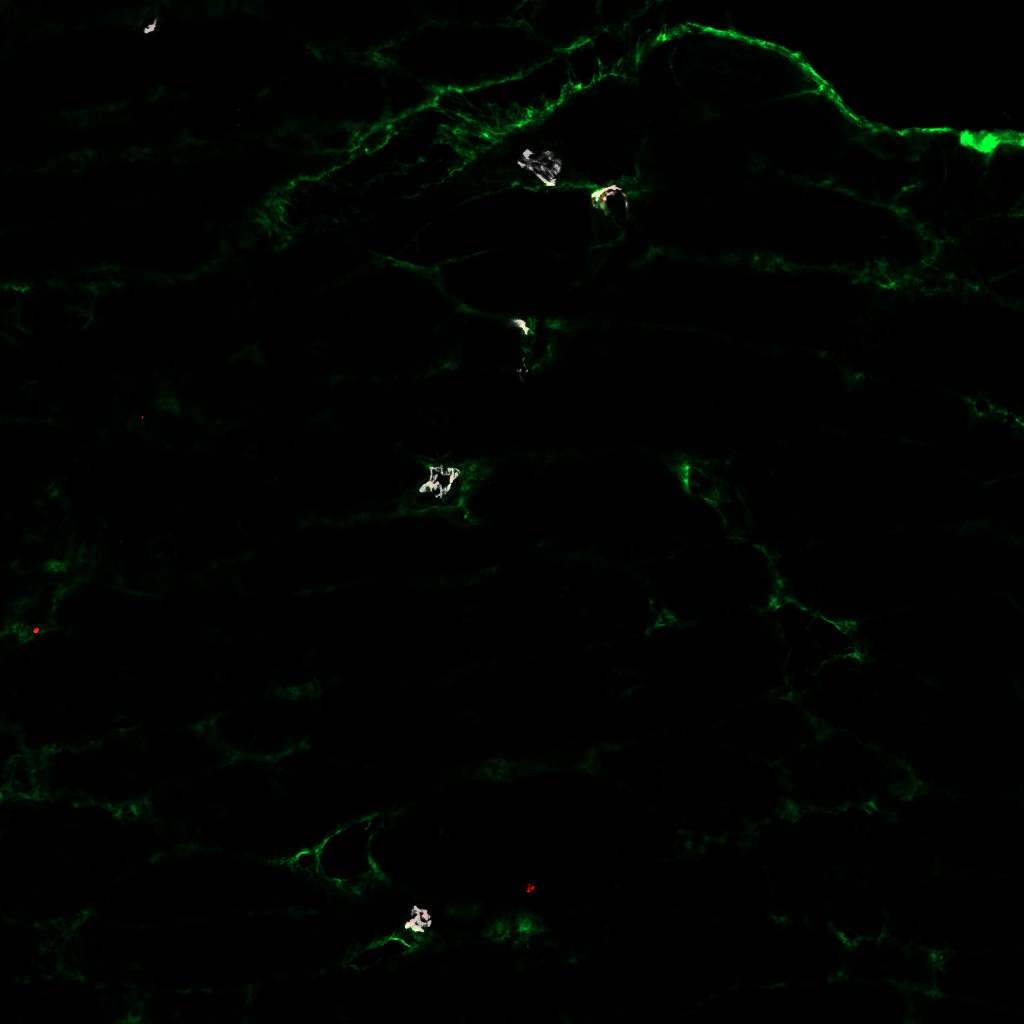

Supplement: Supplementary file 4 [file Data_Sheet_5.ZIP › Fig.5/NMJ-SOD1G93Amice injected with AAV9-GFP+2.jpg]

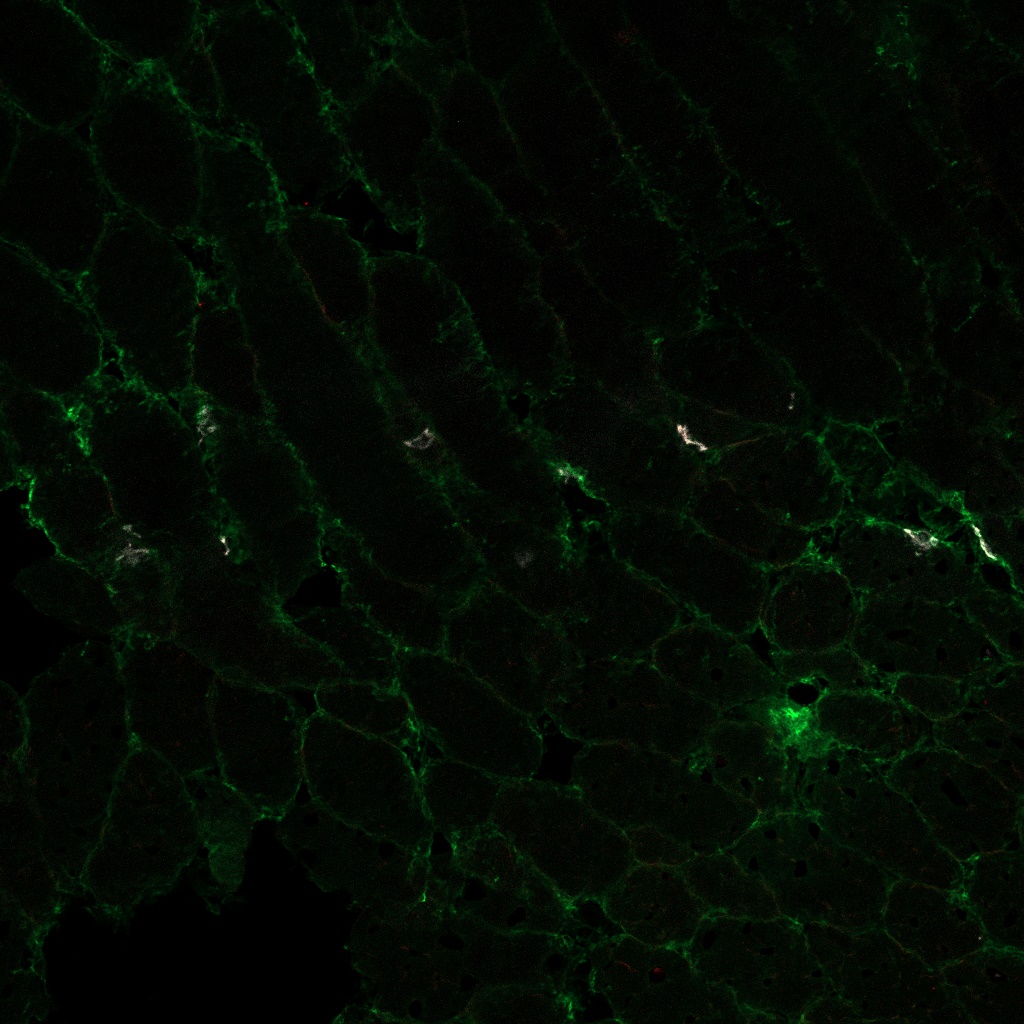

Supplement: Supplementary file 4 [file Data_Sheet_5.ZIP › Fig.5/NMJ-SOD1G93Amice injected with AAV9-GFP+5.jpg]

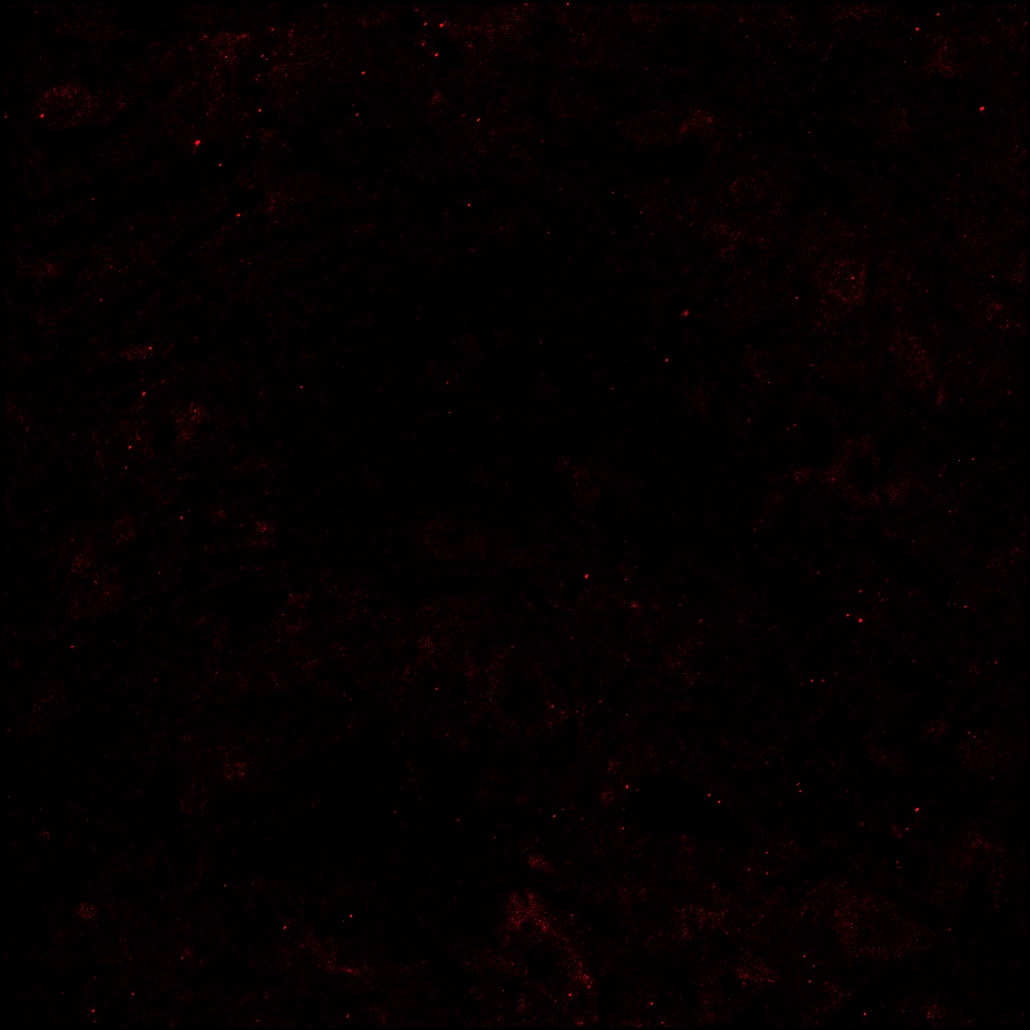

Supplement: Supplementary file 5 [file Data_Sheet_6.ZIP › Fig.6/SOD1 protein in WT group mice 1.jpg]

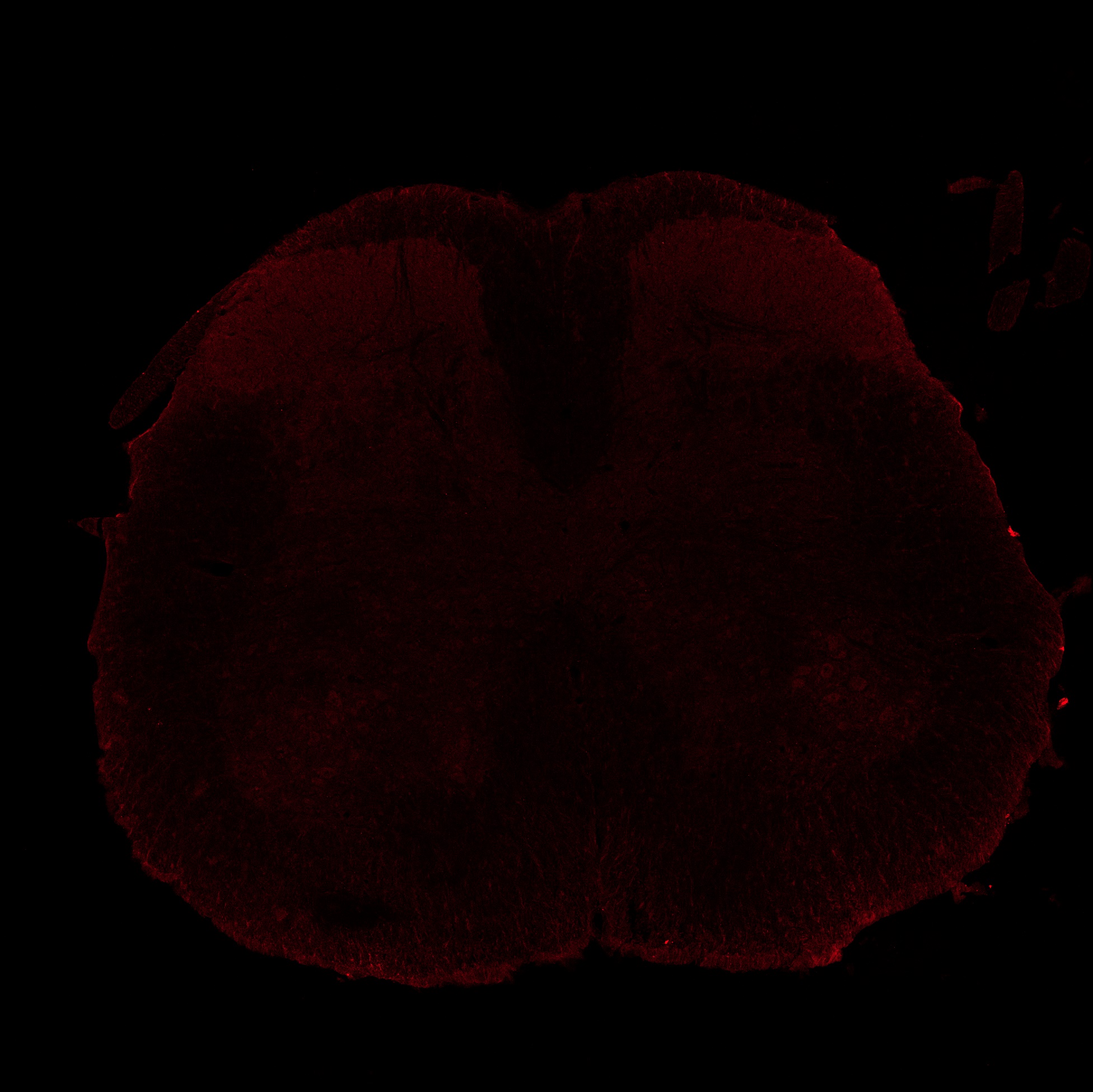

Supplement: Supplementary file 5 [file Data_Sheet_6.ZIP › Fig.6/SOD1 protein in WT group mice.jpg]

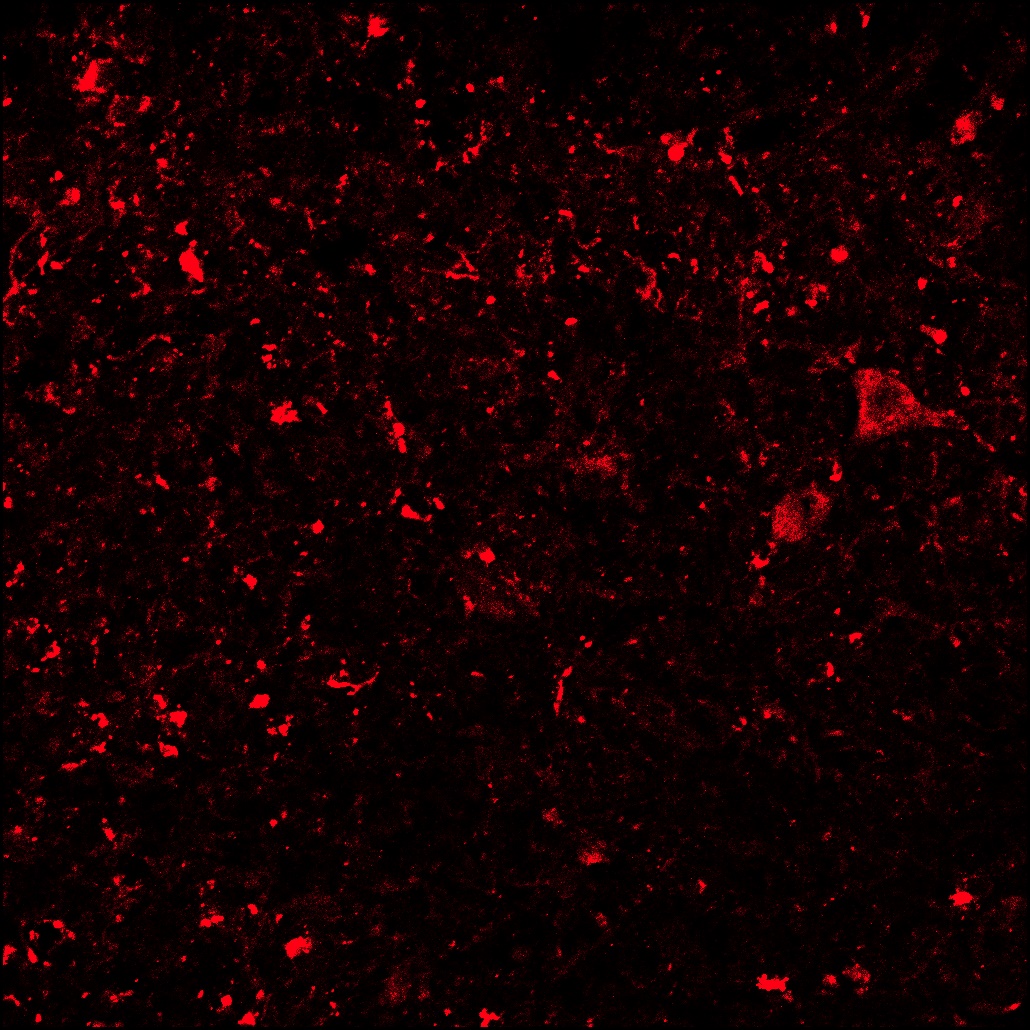

Supplement: Supplementary file 5 [file Data_Sheet_6.ZIP › Fig.6/SOD1 protein in control group mice 1.jpg]

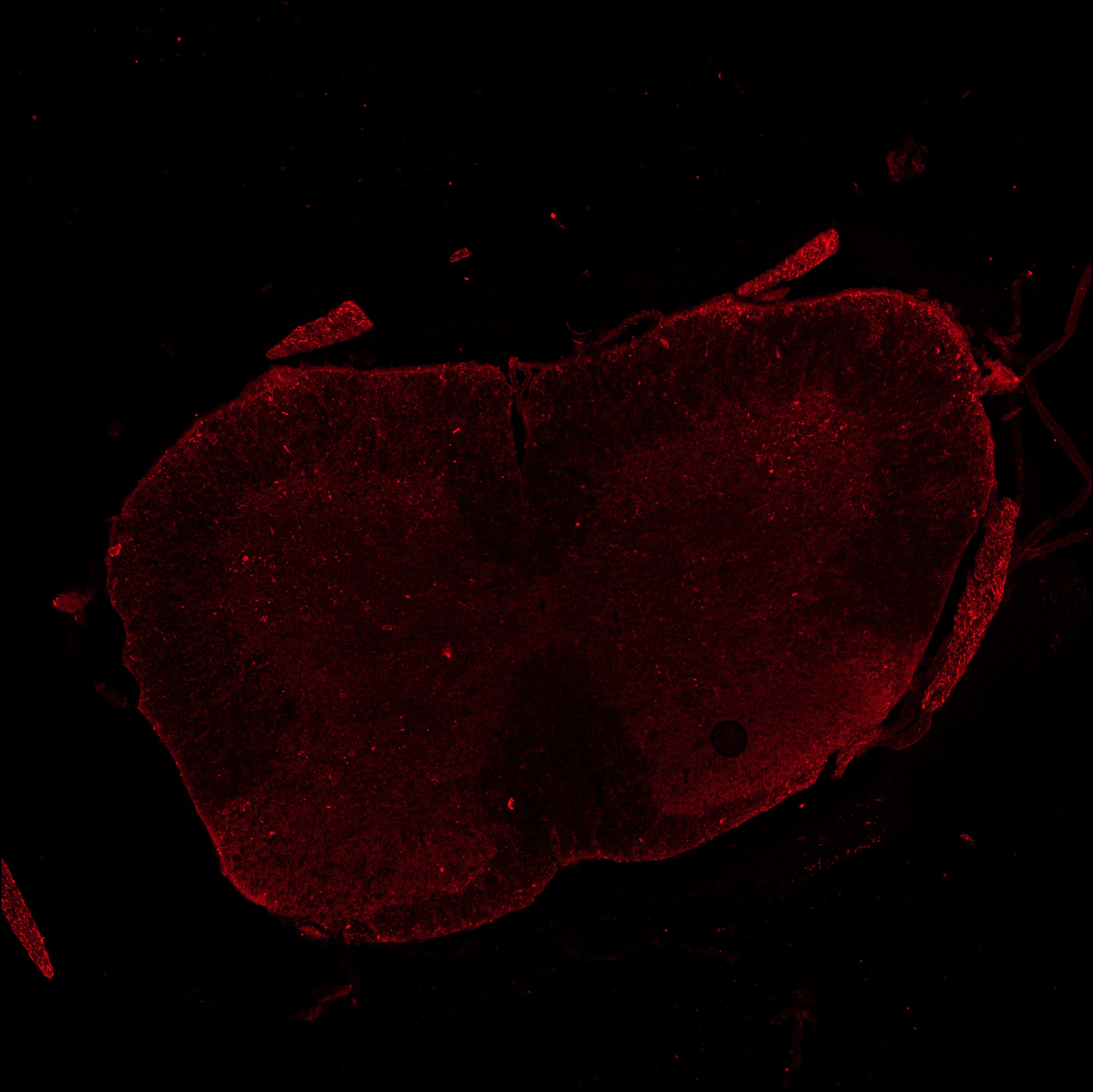

Supplement: Supplementary file 5 [file Data_Sheet_6.ZIP › Fig.6/SOD1 protein in control group mice.jpg]

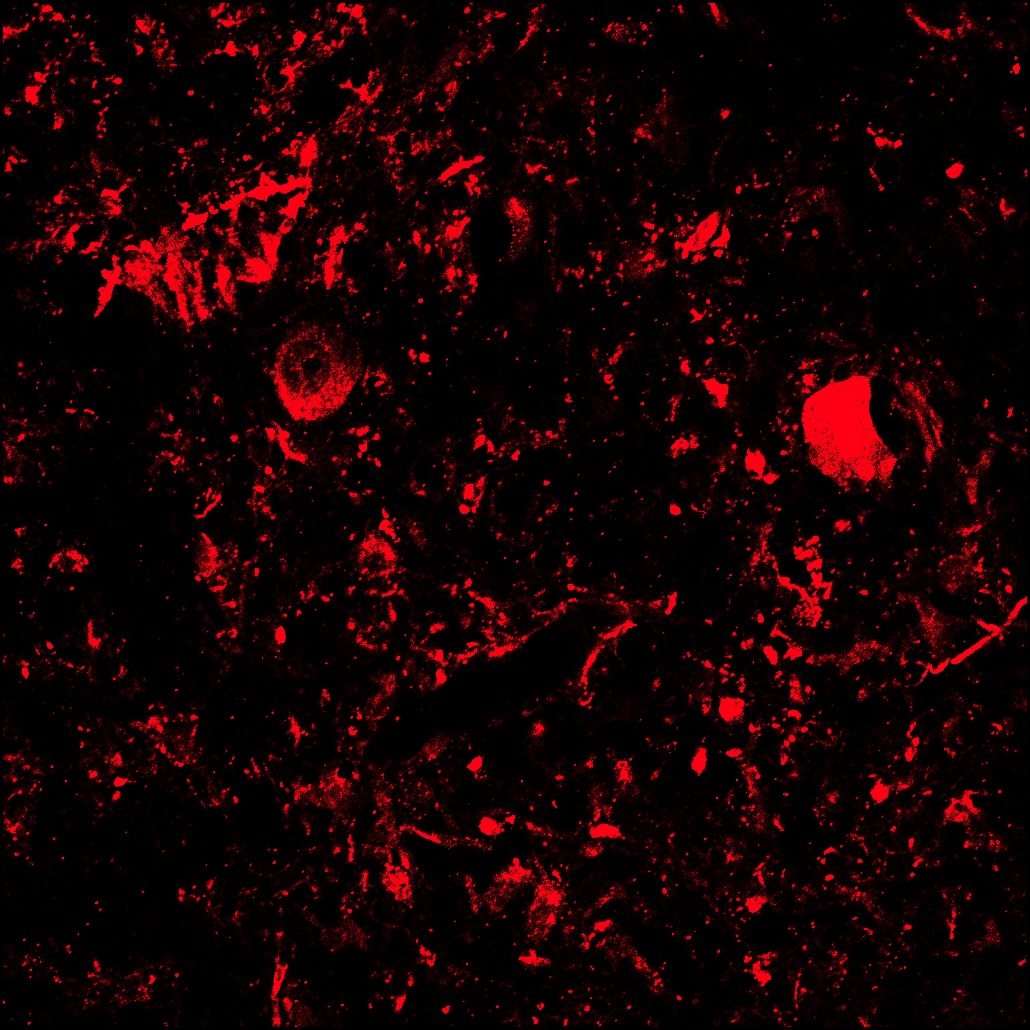

Supplement: Supplementary file 5 [file Data_Sheet_6.ZIP › Fig.6/SOD1 protein in non-injected group mice 1.jpg]

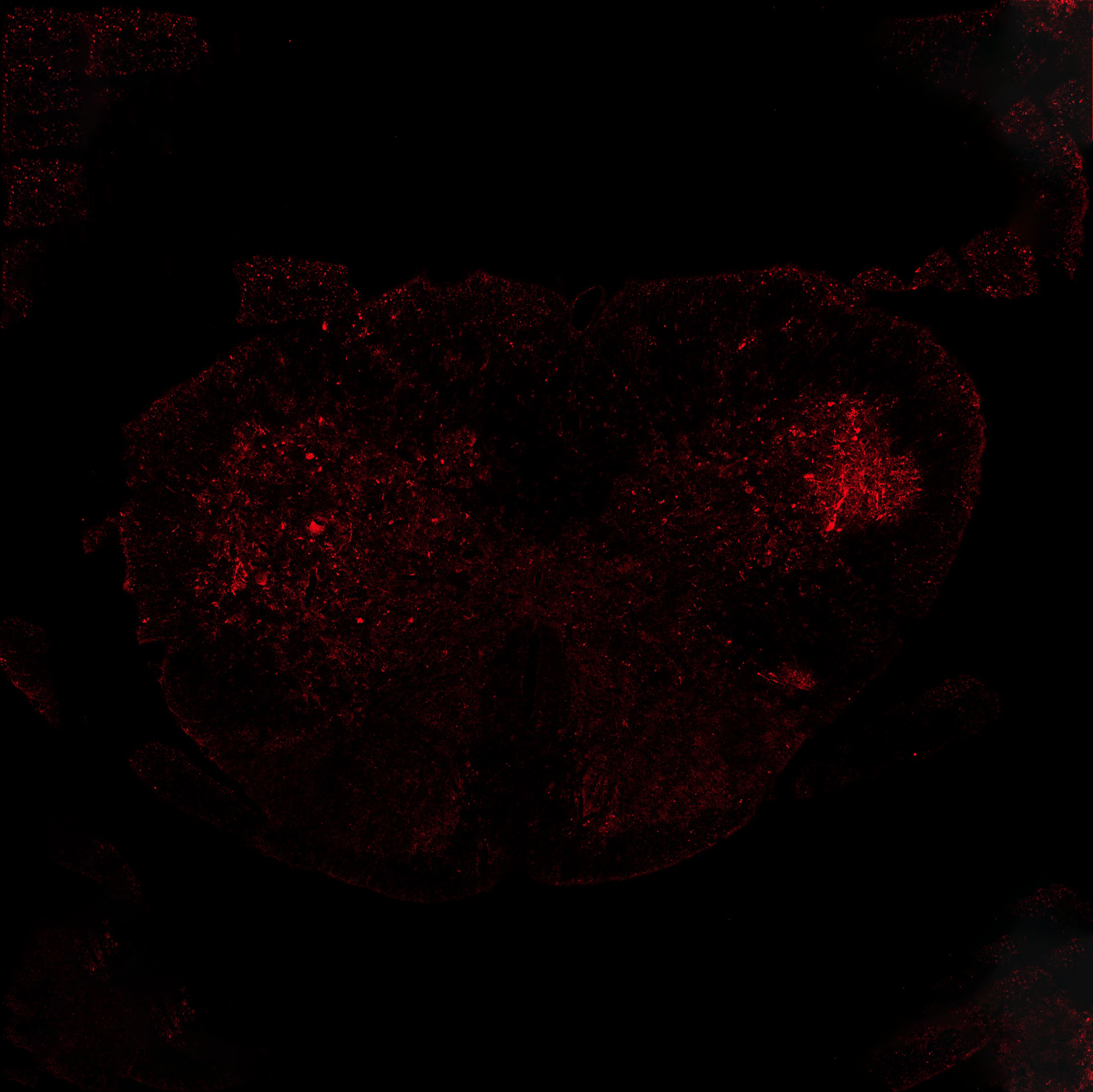

Supplement: Supplementary file 5 [file Data_Sheet_6.ZIP › Fig.6/SOD1 protein in non-injected group mice.tif]

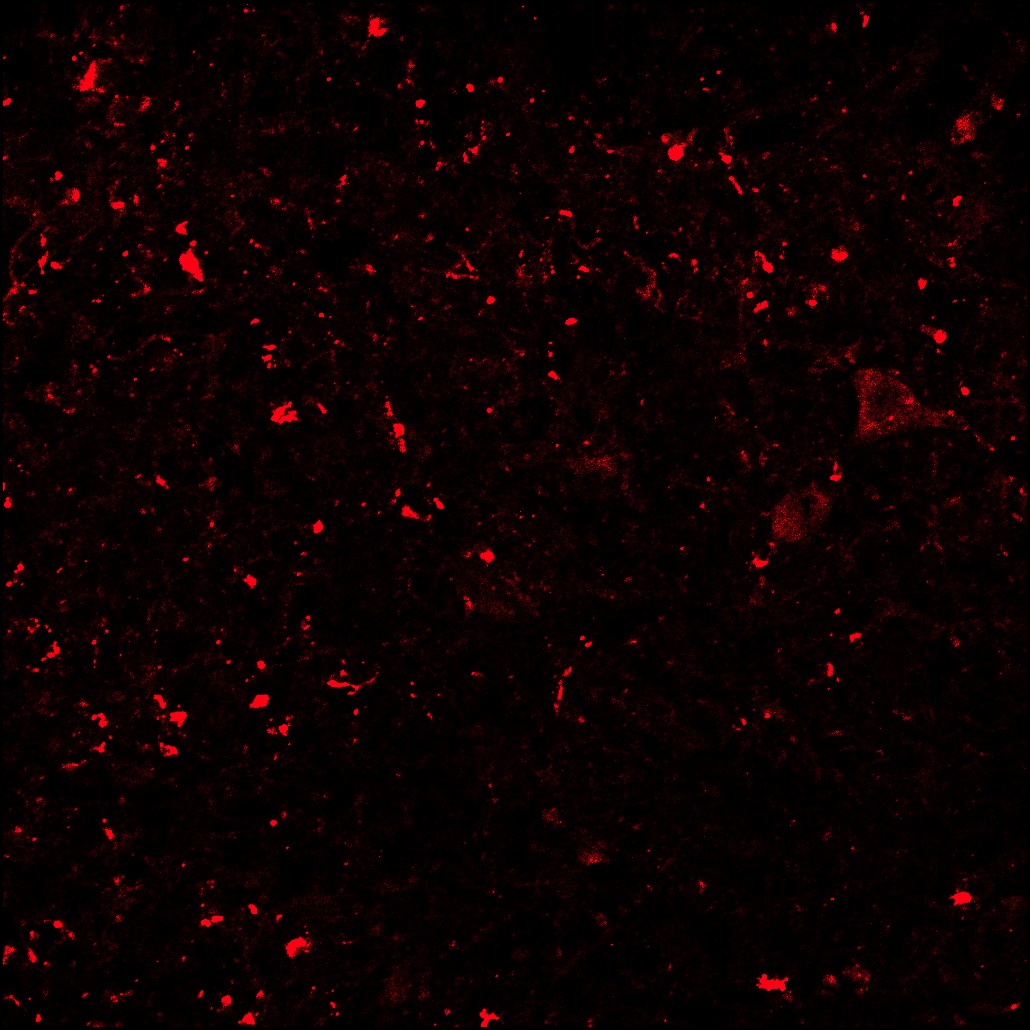

Supplement: Supplementary file 5 [file Data_Sheet_6.ZIP › Fig.6/SOD1 protein in treat group mice 2.jpg]

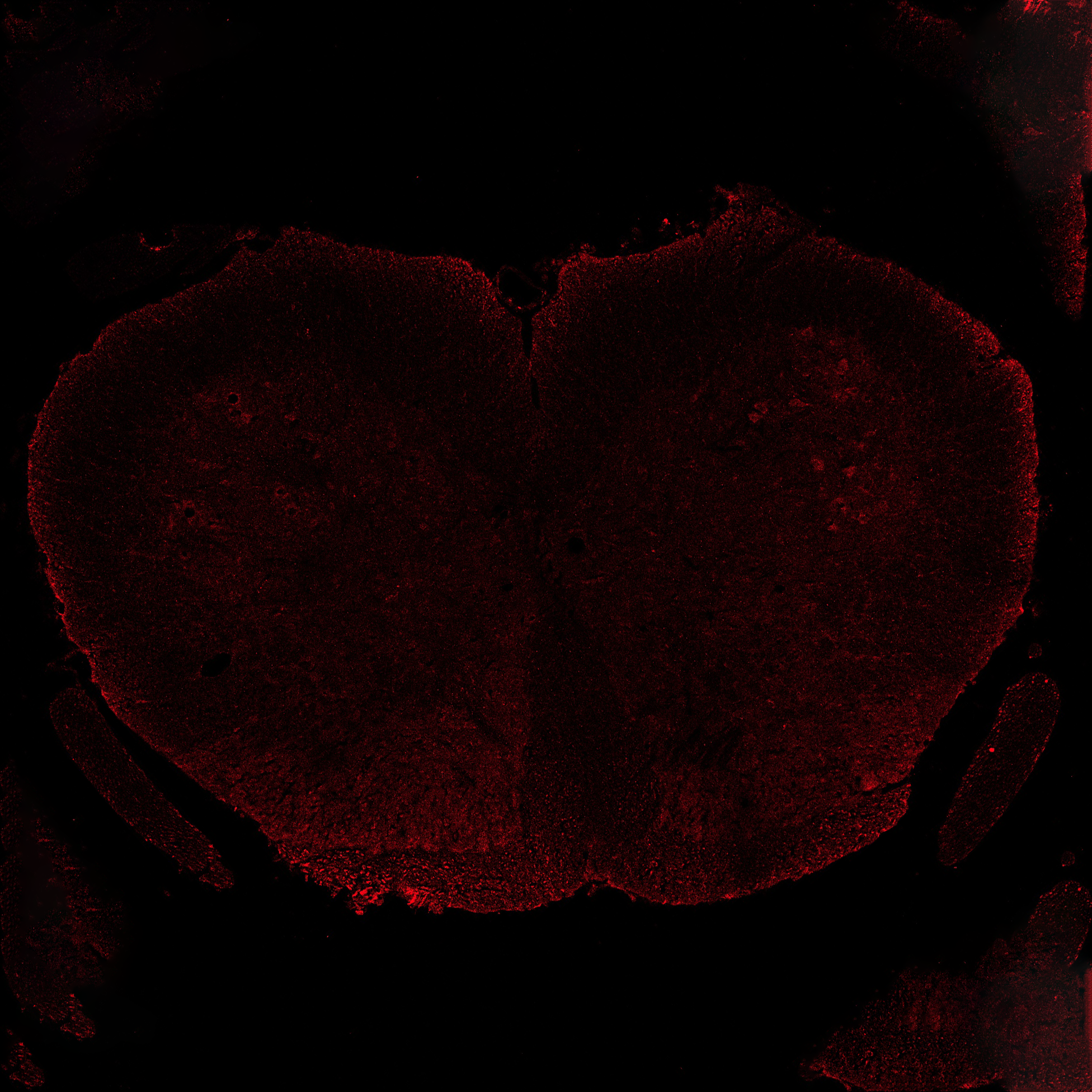

Supplement: Supplementary file 5 [file Data_Sheet_6.ZIP › Fig.6/SOD1 protein in treat group mice.tif]
